# Testing the adaptive radiation hypothesis for the lemurs of Madagascar

**DOI:** 10.1098/rsos.161014

**Published:** 2017-01-18

**Authors:** James P. Herrera

**Affiliations:** 1 Department of Mammalogy, Division of Vertebrate Zoology, American Museum of Natural History, New York, NY 10024, USA; 2 Division of Paleontology, American Museum of Natural History, New York, NY 10024, USA; 3 Richard Gilder Graduate School, American Museum of Natural History, New York, NY 10024, USA; 4 Interdepartmental Doctoral Program in Anthropological Sciences, Stony Brook University, Stony Brook, NY 11794, USA

**Keywords:** diversification, macroevolution, ecological divergence

## Abstract

Lemurs, the diverse, endemic primates of Madagascar, are thought to represent a classic example of adaptive radiation. Based on the most complete phylogeny of living and extinct lemurs yet assembled, I tested predictions of adaptive radiation theory by estimating rates of speciation, extinction and adaptive phenotypic evolution. As predicted, lemur speciation rate exceeded that of their sister clade by nearly twofold, indicating the diversification dynamics of lemurs and mainland relatives may have been decoupled. Lemur diversification rates did not decline over time, however, as predicted by adaptive radiation theory. Optimal body masses diverged among dietary and activity pattern niches as lineages diversified into unique multidimensional ecospace. Based on these results, lemurs only partially fulfil the predictions of adaptive radiation theory, with phenotypic evolution corresponding to an ‘early burst’ of adaptive differentiation. The results must be interpreted with caution, however, because over the long evolutionary history of lemurs (approx. 50 million years), the ‘early burst’ signal of adaptive radiation may have been eroded by extinction.

## 1. Introduction

The ecological limits hypothesis posits that species diversity is limited by resource availability, creating a diversity carrying capacity [1]. As diversity increases and reaches the carrying capacity, speciation rates decrease and/or extinction rates increase, leading to a diversity equilibrium [1]. Diversity equilibria can change over time, as exemplified by mass extinction events and adaptive radiations [1]. Adaptive radiations perturb equilibrium dynamics by changing the carrying capacity for species able to exploit unique ecological opportunities; e.g. invading a novel habitat and diversifying owing to open niches and competitive release [2,3]. Speciation and phenotypic evolutionary rates are predicted to be higher in lineages that exploit open resources or habitats than in sister clades without the same ecological opportunity [3,4].

In this paper, I define an adaptive radiation as having the following properties. Organisms exploiting new ecological opportunities are predicted to undergo rapid lineage and phenotypic diversification as they fill open adaptive zones [3–5]. The rates of speciation and phenotypic diversification should be highest at the onset of the radiation, when species are diverging to fill open niches [5,6]. As species accumulate and niches fill, the ecological limits hypothesis predicts that speciation and phenotypic diversification rates should decelerate as a new equilibrium is reached [1,5]. Finally, lineages evolving via adaptive radiation are predicted to exhibit adaptive phenotypic divergence in unique adaptive zones [3]. There are alternative explanations for apparently decelerating diversification rates other than adaptive radiation, however [7]. Decelerating speciation and phenotypic diversification rates are observed in some adaptive radiations [8,9], but factors such as climate change, biogeography and biotic interactions also shape the curves of diversification rates through time [10,11]. Testing alternative mechanisms in nature can clarify the roles of different evolutionary processes generating biodiversity.

**Figure 1.**
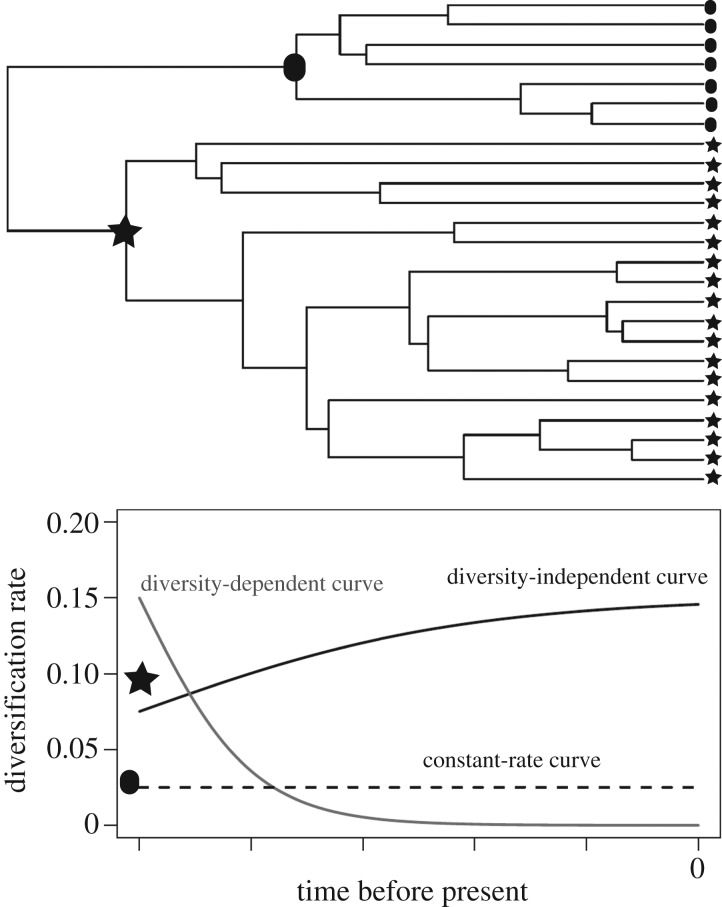
Example phylogeny and expected diversification rates (speciation–extinction) through time. Two clades differ in a trait or geography (circle versus star) that results in the star clade undergoing adaptive radiation. The star clade is predicted to have higher diversification rates (solid lines) than the circle clade (dashed line). The diversification rate of the star clade is predicted to slow towards the present related to niche-filling or other processes [7]. Alternatively, recent processes may have driven an increase in diversification rate of the star clade. The circle clade is predicted to have low and constant diversification over time.

Famous examples of adaptive radiations include *Anolis* lizards in the Caribbean [12], Darwin's finches of the Galapagos [13] and Lake Tanganyika cichlids [2]. These radiations are relatively recent in geological time (less than 40–3 million years ago, Ma) and concentrated in small geographical areas (islands or lakes). Madagascar, the fourth largest island in the world, has been isolated for 90 Ma, during which time clades colonized the island by dispersal [14], and many of the successful colonists exhibit signals of adaptive radiation. The vangas (Aves, Vangidae) had rapid early lineage diversification followed by a deceleration in diversification rate, concomitant with niche divergence followed by stasis [8]. The endemic primates, lemurs, have long been suggested to be an adaptive radiation [15,16]. Lemurs are taxonomically rich with approximately 100 extant species [17] in addition to 17 species that have gone extinct within the last 2000 years [18]. Living lemurs span a range of body sizes from the smallest living primates (30 g *Microcebus*) to around 10 kg, while some recently extinct lemurs were as large as the largest living primate (up to 160 kg). Species vary in their dietary preferences and activity patterns, and 10–14 species live in sympatry with discrete niches [19]. Lemurs belong to the primate clade Strepsirrhini, while the dominant primates in the rest of the world belong to Haplorhini (monkeys, apes and humans). The strepsirrhine sister clade to lemurs, the lorises and galagos of Africa and Asia (hereafter lorisiforms) are small, nocturnal, frugi-insectivores and have fewer species than lemurs [20]. These patterns led early researchers to suggest that a generalized, nocturnal strepsirrhine ancestor dispersed to Madagascar and diversified to fill open niches via adaptive radiation [15]. This hypothesis has only partially been tested empirically [21], however. The macro-evolutionary predictions of adaptive radiation theory for speciation and phenotypic evolution can be tested in the framework of the phylogeny [22–24] (figure 1). I inferred phylogenies and divergence times of nearly all lemurs (88%) and lorisiforms with a matrix of nuclear and mitochondrial loci combined with more than 400 discrete morphological characters, under the Bayesian fossilized birth–death process [25], calibrated using 14 recent and 20 ancient fossils in the tree [26]. In this study, I leveraged the most complete phylogeny of strepsirrhines and complete data on phenotypes and ecology to estimate diversification dynamics (speciation and extinction rates), phenotypic evolutionary rates and adaptive phenotypic divergence among unique ecological niches.

### 1.1. Testing the predictions of adaptive radiation theory

Based on the ecological theory of adaptive radiation [3,5] and the ecological limits hypothesis [1], I tested the following predictions.

(1) If lemurs evolved via an adaptive radiation, then I predict the lineage speciation rates would have increased rapidly following the colonization of Madagascar. Subsequently, as species diversity ‘carrying capacity’ (the total number of lineages that the environment can support) was approached, I predict that the speciation rate should have slowed to near zero. Alternatively, if environmental change or biotic interactions have affected diversification more recently, then I would instead expect that lemurs might show an increasing speciation rate towards the present, rather than the aforementioned pattern of ‘early burst’ followed by a slowdown in rate. Lastly, a mass extinction event may have perturbed diversification dynamics, with a spike in extinction eroding the signal of an ‘early burst’. For example, global cooling and drying during the end of the Eocene and beginning of the Oligocene epochs [27] have been associated with major extinction events in northern hemisphere primates [28]. This extinction event may also have affected lemurs.
(2) If the diversification of lemurs is an adaptive radiation, then I predict speciation rates would have been higher in lemurs than in their sister clade, the lorisiforms. By contrast, if lemurs and lorisiforms had similar ecological opportunity, then I predict speciation rates in lemurs would not have been higher than those in close relatives.
(3) If the diversification of lemurs is an adaptive radiation, then I predict that the rate of phenotypic evolution was initially high but would have declined through time towards the present. By contrast, if environmental or developmental constraints prevented phenotypic diversification, I predict a constant or gradual rate of phenotypic evolution.
(4) If lemurs evolved via adaptive radiation, then I predict their phenotypes diverged in relation to shifts in niches of diet and activity pattern (adaptive zones). I predict optimal phenotypes, rates of evolution and evolutionary constraints differ among niches. If lemurs exhibited adaptive phenotypic and niche divergence, then I predict lemurs should occupy a wider breadth of multidimensional ‘ecospace’ than lorisiforms. If lemur phenotypic evolution was constrained by other forces, e.g. genetic constraints, then I predict phenotypes would not differ markedly among adaptive zones and the ecospace occupied by lemurs should be no different from that of lorisiforms.

## 2. Methods

### 2.1. Phylogeny

To test the predictions of adaptive radiation theory, I used a phylogenetic comparative approach [29]. I leveraged recently inferred near-complete dated phylogenies of lemurs (*n* = 101) and lorisiforms (*n* = 19) with multiple primate outgroups [26]. The phylogenies were inferred based on a combined matrix of four nuclear and two mitochondrial genes (5767 base pairs) and 421 discrete morphological characters. The tree was dated using the fossilized birth–death model [25] implemented in MrBayes [30], with 20 ancient fossils and 14 recently extinct lemurs calibrating the birth–death process. I assessed the impact of changes in the topology and branch lengths on the results by conducting analyses with the following alternate trees: the maximum clade credibility (MCC) tree, 100 trees randomly selected from the posterior distribution, the MCC tree with an alternative placement of the subfossil genus *Megaladapis* following [31], the MCC tree of extant species only, the MCC tree comprised of only taxa accepted under a more conservative taxonomy, given some skepticism in the recent increase in species richness [32], and the strepsirrhine tree based on mitochondrial genomes with extinct lemurs [31] (electronic supplementary material, files S1–S5).

### 2.2. Diversification dynamics

I estimated the speciation and extinction rate dynamics on the tree using several recently developed methods. First, I tested for shifts in rates across the tree and through time using Bayesian analysis of macroevolutionary mixtures (BAMM [23]). Briefly, BAMM implements a birth–death model and tests for shifts in diversification dynamics across the tree and through time. A single diversification rate regime for the whole tree is assumed *a priori.* More complex models are explored by adding unique diversification regimes to the tree and comparing the probabilities of models with rate shifts to the single diversification model using a reversible-jump Markov chain Monte Carlo algorithm. Missing species were accommodated by specifying genus-level sampling fractions (electronic supplementary material, table S1). BAMM also uses the same search algorithm to test for shifts in the evolutionary rate of phenotypic traits, assuming a Brownian motion model of trait evolution. I used this phenotypic algorithm to estimate the evolutionary dynamics of body mass (see below for body mass details). The models implemented in BAMM assess multiple possible diversification rate shift scenarios, but assumes there are no diversification rate shifts on lineages that go extinct. This assumption causes error in parameter estimates in trees simulated to have rate shifts on extinct lineages, but not for trees simulated to have constant rates [33]. The effect of this assumption, common to all diversification rate shift analyses available currently, is suggested to be small in empirical trees [34]. Another concern is that the posterior distribution on the number of rate shifts inferred by BAMM may be overly sensitive to the prior probability on the number of diversification rate shifts. This concern has been addressed in the most recent versions of BAMM (v. 2.5) which was used in this analysis [35]. The results of my analyses are consistent with a strong prior on few rate shifts or a vague prior across many rate shifts (electronic supplementary material).

In addition to the speciation and extinction analysis in BAMM, I estimated diversification dynamics using an alternative approach: modeling evolutionary diversification using stepwise Akaike information criterion, MEDUSA [22] implemented in the *geiger* package [36] for the statistical environment R [37]. MEDUSA first assumes a single pure-birth or birth–death process and then tests for shifts in rates across the tree by iteratively adding breakpoints and re-estimating diversification model parameters for subclades. The model of diversification with multiple regimes improves the fit to the data based on second-order Akaike information criterion (AICc). MEDUSA assumes a constant rate of diversification within each regime. The genus-level species richness was specified by assigning missing species evenly to tips of each incompletely sampled genus. MEDUSA is prone to falsely identifying rate shifts on trees simulated to have a single diversification history [38]. To account for this, I simulated 100 trees to have a single diversification rate history (lambda = 0.15, mu = 0.05, based on results of BAMM analysis). I used the minimum ΔAICc value that correctly rejected rate shifts as the threshold for adding rate regimes to the empirical tree (electronic supplementary material).

Neither of the above methods tests the possibility of a dramatic shift in extinction, as would be predicted if a mass extinction event perturbed diversification dynamics. Such an extinction event may erode the signal of past diversification history, making it unlikely to detect the signature of an adaptive radiation. I tested the hypothesis that extinction rate increased markedly at the Eocene–Oligocene boundary (33.9 Ma) by comparing the probability of an extinction rate shift at that time period to the probability of no rate shift in a Bayesian framework (COMET algorithm, implemented in the R package *TESS*, [39]; electronic supplementary material).

Lastly, I tested the relative likelihoods of multiple diversification models including those with changing speciation and/or extinction rates through time and in relation to an independent variable [40,41] using the package Phylogenetic Analysis of Diversification, *RPANDA* [42] in R. The diversification models in *RPANDA* are implemented in a likelihood framework, enabling multimodel inference approaches [43]. As potential correlates of diversification dynamics, I tested the effects of two continuous independent variables on speciation rates: the estimated temperature throughout the Cenozoic [27], and the past diversity of Malagasy carnivores, estimated based on an extant lineage-through-time plot and the phylogeny from [44]. I compared the diversification models implemented in *RPANDA* for the whole strepsirrhine tree and for lemurs and lorisiforms separately.

To assess the adequacy of the diversification rate models to describe the empirical trees, I simulated three sets of 1000 trees based on the results of the diversification analyses: (i) a single time-constant diversification rate, (ii) two time-constant rates, and (iii) a single time-varying speciation rate and constant extinction rate. For each tree, I calculated summary tree statistics and compared the values from the simulated trees to the empirical tree to determine if the macro-evolutionary model parameters could generate trees similar to the empirical tree (electronic supplementary material).

### 2.3. Adaptive phenotypic evolution

To determine if phenotypic evolution was adaptive, I compiled a database on strepsirrhine body mass, diet and activity pattern (electronic supplementary material, table S2). I tested if variation in body mass could be explained by a combined diet and activity pattern niche variable using phylogenetic generalized least-squares (PGLS) regression in the *nlme* package [45] with functions from *ape* [46] for R. Four models of trait evolution were compared: Brownian motion, Pagel's lambda, Ornstein–Uhlenbeck (OU) and Blomberg's g. I considered the model with the lowest AICc scores and ΔAICc scores less than 3 the best models, and I averaged model parameters by summing the parameters across models weighting by the AICc weights [43]. I assessed the adequacy of the models by rescaling the tree based on the evolutionary model, simulating traits on the rescaled tree, and comparing test statistics between the observed and simulated traits [47] (electronic supplementary material). These model adequacy tests revealed that the empirical data deviated from the simulated data in statistics related to heterogeneity across the tree.

To accommodate this heterogeneity, I used a generalized OU model framework [48] in which the evolutionary history of the diet/activity pattern niche trait was estimated at internal nodes and the niche regimes mapped onto the tree. I then compared the fit of the following models: a single Brownian motion model for the entire tree, a single OU model for the tree, unique Brownian motion model parameters for each niche regime on the tree, multiple unique OU model parameters for each niche regime on the tree. To assess whether uncertainty in the trees or the ancestral state estimates affected the results, I also used stochastic mapping [49] as implemented in *phytools* [50] to simulate the evolution of the niche trait on each of the 100 trees from the posterior distribution 100 times and compare single and multiple evolutionary rate models across 100 random samples from the stochastic maps.

To characterize the multidimensional ecospace occupied by strepsirrhines and determine how that ecospace has changed as a result of the extinction of giant lemurs, I converted the trait data into a dissimilarity matrix based on the Gower coefficient [51] and accounted for phylogeny by extracting the residuals of a matrix regression of trait dissimilarity on phylogenetic distance. I projected the residual trait dissimilarity onto two axes using non-metric multidimensional scaling [52] and created convex hulls around extant and extinct species using R functions [37].

## 3. Results

The major findings are presented in line with the predictions in the Introduction.

**Figure 2.**
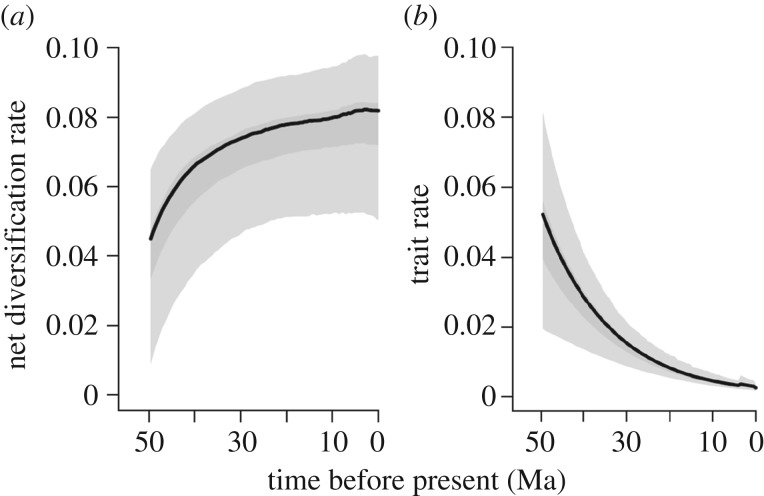
Rate-through-time plots. Graphs illustrate the mean instantaneous rate estimate for lemurs through time (black line), with 95% credible intervals (CIs) (grey shading). (*a*) The net diversification rate (speciation–extinction rate) increased towards the present and did not exhibit decreasing rates towards zero as expected if speciation was diversity-dependent and nearing equilibrium. (*b*) The rate of body mass evolution (*σ*^2^ Brownian motion variance) exhibits the predicted early burst of high evolutionary rates early and decreasing rates towards the present consistent with the hypothesis that lemurs diversified in body mass most early after colonizing Madagascar and subsequent body mass evolution was low.

(1) Estimates of diversification rate variation through time did not support the prediction of high lineage diversification rates early followed by a decline in rates towards the present. Instead, initial diversification rates were low and increased towards the present, with no evidence of a slowdown in rates (figure 2*a* and table 1). Time-varying and constant-rate models had similar probabilities, however, making discrimination of alternative diversification scenarios difficult (table 1; electronic supplementary material, figure S2, table S3 and Results). There was no evidence of a net diversification rate of 0 lineages My^−1^, nor decelerating diversification through time, and results were comparable using different phylogenies, assuming high or low species richness, assuming different models of temporal variation in speciation rates, and using a range of prior probabilities for the number of rate regimes (table 1; electronic supplementary material, table S3 and Results). The empirical trees had temporal structure suggesting increasing diversification rates through time (table 1; electronic supplementary material, figure S3 and Results). I tested if palaeoenvironmental change or the arrival and diversification of carnivores could explain the rate variation through time estimated by the *RPANDA* analyses. Models of diversification rate variation dependent on changes in global temperature during the Cenozoic [27] or dependent on past carnivore diversity did not fit the data better than birth–death models independent of climate or carnivores (table 1; electronic supplementary material, table S3). I tested for an increase in extinction rates at the Eocene–Oligocene boundary (33.9 Ma) that would correspond to a hypothesized mass extinction event and found no evidence for a shift in extinction rates (electronic supplementary material, figure S4 and Results). Given these results, it appears unlikely that a mass extinction event would have caused the patterns of branching times in the empirical phylogenies.
(2) Supporting the prediction from adaptive radiation theory, the speciation rate estimated for the crown lemurs (approx. 0.15 lineages My^−1^) was double the rate estimated for crown lorisiforms (approx. 0.08 lineages My^−1^, rates estimated using *RPANDA* [42]). Models in which speciation rate increased over time had slightly higher probabilities for lemurs than constant-rate models, while for lorisiforms a constant-rate pure-birth model had the highest probability (table 1; electronic supplementary material, table S3 and Results). Given the small size of the lorisiform tree (16 tips), the support for the model with the fewest parameters (pure-birth) may also be owing to low statistical power to detect changes in diversification rate-through time. These findings were supported when iterating the model comparison across 100 trees from the posterior distribution of trees, with further support for different diversification dynamics between lemurs and lorisiforms (lemur model-averaged mean speciation rate = 0.16 lineages My^−1^, s.d. = 0.07, 95% CI = 0.147–0.177, lorisiform model-averaged mean speciation rate = 0.08 lineages My^−1^, s.d. = 0.03, 95% CI = 0.073–0.084; electronic supplementary material, Results). These results suggest that diversification dynamics in lemurs and lorisiforms may have been decoupled. Analyses of the whole strepsirrhine tree using automated detection of diversification shifts did not support a significant shift between lemurs and lorisiforms, however, suggesting that the support for a shift in rates is equivocal (BAMM and MEDUSA analyses, electronic supplementary material Results and tables S4, S5). Instead, a significant rate shift was inferred for the lemur genus *Microcebus*, which exhibited higher speciation rates than expected compared with the background speciation rate of the tree (electronic supplementary material, Results and figure S1).
(3) Concordant with predictions of adaptive radiation theory, the rate of lemur body mass evolution was highest after the colonization of Madagascar and declined towards the present (figure 2*b*; electronic supplementary material, table S7 and Results). The ‘early burst’ pattern has been difficult to detect with real datasets [53]. There was no evidence for a shift in evolutionary rates between lemurs and lorisiforms, however (electronic supplementary material, Results, figure S5 and table S6). Comparison of the posterior probabilities of the time-varying and time-constant phenotypic evolutionary rate models suggests the two models have nearly identical probabilities, however (electronic supplementary material, figure S6).
(4) Testing if phenotypes diverged adaptively, phylogenetic regressions support the prediction that body mass differed among adaptive zones (electronic supplementary material, Results and figure S7); nocturnal folivores and nocturnal omnivores had lower body masses than the mean, and the best-fitting phenotypic evolution models were Blomberg's g (early burst) and OU (AICc weights = 0.48 and 0.38 respectively; electronic supplementary material, Results). These results support the early burst prediction that lineages co-evolved body mass with diet and activity niches, and that the rate of body mass evolution decelerated over time. Assessment of model adequacy, however, suggested there was rate heterogeneity across the tree (electronic supplementary material, table S8 and figure S8).

**Table 1.** Model-averaged speciation and extinction rate parameters (per lineage no. new lineages My^−1^). (Models of diversification dynamics in which rates were time-constant or time-variable [24] were compared for the entire strepsirrhine tree, and for the lemur and lorisiform crown clades separately. Model-averaging [43] was performed by summing the parameter values weighted by their Akaike information criterion weights. For complete results, see the electronic supplementary material, table S3.)

tree	best model(s)	speciation (lineages My^−1^)	extinction (lineages My^−1^)	time-varying function
strepsirrhine tree (*n* = 120)	linear or exponential increase in speciation, 0 extinction; constant birth–death	0.142	0.024	−0.008
lemur tree (*n* = 101)	linear or exponential increase in speciation, 0 extinction; constant birth–death	0.152	0.008	−0.01
lorisiform tree (*n* = 19)	constant pure birth	0.081	0.0003	−0.0005

To account for this heterogeneity in rates, I used generalized state-dependent OU models [48] to test if patterns of body mass evolution differed among adaptive zones. The results support adaptive constraint around body mass optima, which differed according to adaptive zones of diet and activity pattern (table 2; electronic supplementary material, figure S9 and table S9). The best model of body mass evolution specified unique adaptive optimal phenotypes and evolutionary rates for each adaptive zone, but with one adaptive constraint parameter across adaptive zones (table 2; electronic supplementary material, table S9), while the next-best model had independent OU models fit to each adaptive zone reconstructed on the tree (electronic supplementary material, table S9). The state-dependent OU model with unique constraint parameters for each state was favoured most often across 100 trees from the posterior distribution with niches mapped using stochastic character simulation to account for uncertainty in the topology, branch lengths and ancestral state estimation (electronic supplementary material, table S10). I recognize, however, that despite the sample size of more than 100 species, the number of independent evolutionary shifts into distinct niches is limited; niches are conserved for most family-level clades, which may limit the power of these comparative methods. The results lend support for the adaptive phenotypic differentiation of lineages, although they do not conclusively support adaptive divergence.

**Table 2.** Results of model comparison testing body mass evolutionary dynamics. (The following models were tested: a single Brownian motion model (BM), a single Ornstein–Uhlenbeck model (OU), a different BM model for each state of adaptive zones (state-dependent BM), and the following state-dependent generalized OU models: 1: unique optima for body mass in each adaptive zone; 2: unique optima and constraint parameters pulling the trait value towards the optima; 3: unique optima and evolutionary rate parameters; 4: unique optima, constraint parameters and evolutionary rates. The best models were the state-dependent OU 3 and OU 4 (in italics). ΔAICc: the difference in the second-order Akaike Information Criterion score from the best model; AICc w: AICc weights; *n* = 119 species.)

model	ΔAICc	AICc w
Brownian motion (BM)	42.60	2.96 × 10^−10^
Ornstein–Uhlenbeck (OU)	44.71	1.03 × 10^−10^
state-dependent BM	12.96	8.09 × 10^−4^
state-dependent OU 1	14.84	3.16 × 10^−4^
state-dependent OU 2	13.87	5.11 × 10^−4^
*state-dependent OU 3*	*0.00*	*0.53*
*state-dependent OU 4*	*0.22*	*0.47*

**Figure 3.**
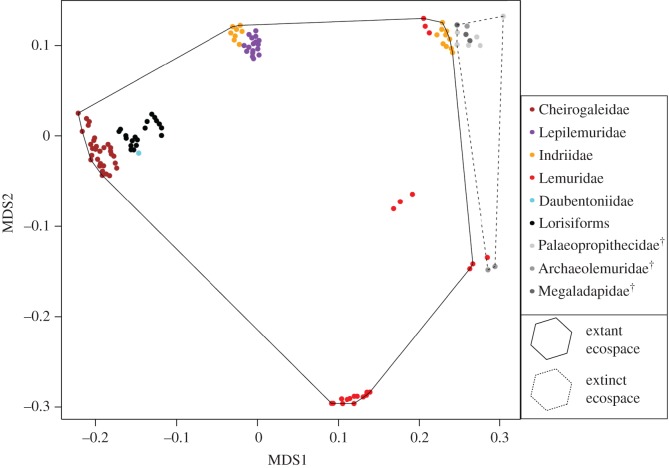
The multidimensional ecospace. The niche breadth occupied by lemurs far exceeds that of lorisiforms (black points), but the occupied ecospace has contracted substantially because of the extinction of the giant lemurs. Variation among species in (natural log) body mass, diet and activity pattern were summarized into two axes using multidimensional scaling (MDS1 and 2, approximately 90% of variation). Each lemur family (coloured points, extinct in grey with crosses in legend) occupies distinct ecospace, within which lorisiforms fill only a small proportion. The giant extinct lemurs filled unique ecospace (dashed convex hull) beyond that of extant species (solid convex hull).

As expected for an adaptive radiation, lemurs diversified to fill a wider breadth of multidimensional ecospace than lorisiforms (figure 3). Two families of lemurs, Cheirogaleidae and Daubentoniidae, have similar niche space to lorisiforms in that they are nocturnal and feed on insects as well as plant parts like fruit, which is most likely the shared primitive condition. Other lemur lineages then differentiated to occupy ecospace that far exceeds that of lorisiforms. The ecospace has contracted drastically since the extinction of 17 lemurs because of the loss of the largest species (figure 3).

## 4. Discussion

### 4.1. Are lemurs an adaptive radiation?

This study tested the long-held hypothesis that lemurs are an adaptive radiation and sheds light on the dynamics of lineage and phenotypic diversification. I found little evidence that lemur diversification dynamics fit the predictions of adaptive radiation theory. There was partial support for the prediction that lemur lineage diversification was higher than lorisiform rates—lemur speciation rates were twice that of lorisiforms, though a significant shift in diversification dynamics was not detected across the whole strepsirrhine tree. Contrary to predictions, there was no support for declining speciation or diversification rates, as would be expected if an equilibrium had been reached. Supporting the adaptive radiation hypothesis, lemur body mass evolution exhibited the ‘early burst’ pattern of high rates of change after colonizing Madagascar and declining rates towards the present, despite the fact that the ‘early burst’ pattern of phenotypic evolution is rarely observed in empirical datasets [53]. Lorisiforms, on the other hand, had comparatively constant, low rates of phenotypic evolution. Concordant with the adaptation predictions, body mass evolved at different rates and towards unique optima in adaptive zones related to diet and activity pattern. Lemurs differentiated in the ecospace they occupy, far exceeding the niche breadth of lorisiforms. From these results, I argue that lemur phenotypic evolution exhibited properties of an adaptive radiation early in their divergence from lorisiforms, but many factors have shaped diversification dynamics over their long evolutionary history.

For an evolutionary radiation to be considered adaptive, the phenotypic evolutionary dynamics must reflect adaptations to rapidly diverging niches [3,4]. Indeed phenotypes may preserve the signals of adaptive radiation better than lineage diversification dynamics because of the confounding factors obscuring patterns of diversification, as observed in cetaceans [54]. The results of this study provide support for an adaptive relationship between body mass and diet/activity pattern; diurnal folivorous and omnivorous lineages evolved towards high body mass optima, while nocturnal folivorous and omnivorous lineages evolved towards lower body mass optima. Body mass is strongly correlated to diet in mammals [55,56]; herbivores are larger than omnivores or insectivores because of the relative differences in energy demands and physiological adaptations to digestion. While many folivorous lemurs are large-bodied (including the giant extinct lemurs [57]), three clades of lemurs are small-bodied folivores (approx. 1 kg), two of which are nocturnal. Small-bodied folivores have unique anatomical, physiological and behavioural adaptations to their low-nutrient diets [19,58]. These patterns are consistent with the hypothesis that initial differentiation into adaptive zones was coupled with shifts in body size. There were few independent shifts in diet and activity pattern, however, limiting the power of these analyses, and future work with a sample of all primates may help to discern if adaptive divergence has occurred.

### 4.2. Why was there no decline in lineage diversification rates?

The ecological limits hypothesis predicts that diversification rates decrease with increasing species richness as speciation and extinction rates reach a diversity-dependent equilibrium [1]. I observed no decline in diversification rates in lemurs or lorisiforms, which may suggest that there are no ecological limits, or that lemurs have not reached a carrying capacity, even after 50 million years. There are other evolutionary mechanisms and methodological artefacts that can drive slowdowns in diversification rates [7], and our inferences of diversification rates are only samples of a continuum of changing speciation and extinction rates. Using simulations, it was shown that clades evolving under diversity-dependent speciation and constant extinction exhibited slowdowns upon reaching equilibria, but after longer time periods, extinction eroded the early burst signal and resulted in trees with nodes concentrated towards the tips [1]. If extinction rates were high in lemur evolutionary history, then the phylogenetic methods used in this study may not be able to detect the patterns of past diversification. For example, during the Cenozoic, the end of the Eocene and beginning of the Oligocene is marked by a dramatic shift in global temperatures [27], which has been associated with the extinction of strepsirrhine primates in the northern hemisphere [28], as well as many other taxa [59]. I tested if a mass extinction event may have occurred in lemur evolutionary history and found no evidence for an increase in extinction rates during the Cenozoic that could be interpreted as a mass extinction event. Without fossil evidence, however, discerning alternate diversification hypotheses for lemurs may always be limited. It is possible that other classic examples of adaptive radiations that do show diversification slowdowns (e.g. greater Antillean *Anolis* lizards [60]) may have had low extinction rates or may not be old enough for extinction to have pruned old lineages from the tree.

Less attention has been paid to mechanisms causing increasing diversification rates through time, possibly because increasing rates are much less commonly observed [61]. Evidence from palaeontology, phylogenetic models and community ecology refute equilibrium dynamics [62]. Some explanations for positive lineage diversification rates include a lack of steady-state between hosts and pathogens [11], predators and prey, and competition [63]. I tested one possible biotic correlate of increasing speciation rates: the arrival and diversification of carnivores. Without fossils, it is impossible to know if there were lemur predators on Madagascar before the arrival of carnivorans, but snakes, crocodilians and raptors may have been present or at least dispersal-advantaged during the early Cenozoic [14]. Based on molecular evidence from extant carnivorans, the arrival and diversification of mammalian carnivores on Madagascar is estimated at approximately 20–30 Ma [44]. I hypothesized that carnivorans may have triggered an evolutionary race between lemurs and their predators, but I found that estimated carnivore diversity did not have a significant effect on lemur speciation rate (electronic supplementary material, table S3). Alternatively, rather than cause decelerations in diversification, competition among lemur species for resources may have driven increasing diversification as lemurs partitioned niches along ever finer axes. For example, not only do co-occurring lemurs differ in coarse dietary categories (folivore or omnivore), but multiple sympatric folivores select different plant parts and/or leaves with different secondary compounds [19]. Further investigation of the possible phenotypic or ecological drivers of diversity and diversification rates through time are required to clarify the findings reported here.

Another factor that could explain prolonged positive diversification rates include biogeographic evolution. Geographical isolation and vicariance events lead to speciation [64], and vicariance owing to the fragmentation of suitable habitat driven by climate change can lead to spikes in diversification rates [65]. In some cases, vicariance may lead to a slowdown in diversification rates as the repeated subdivision of ranges affects fewer and fewer species [7]. By contrast, dispersal can lead to high speciation rates as lineages regularly encounter new ecological opportunities [10]. Lemurs most likely colonized Madagascar during a time when cooler, arid environments gradually transitioned to moist tropical conditions as Madagascar drifted north of the Tropic of Capricorn in the Eocene [66]. During this shift in climate, lemurs may have experienced increasing diversification rates as they adapted to new habitats. Global cooling and drying at the beginning of the Oligocene may have led to aridification of Madagascar and forest fragmentation with subsequent vicariance. I tested a model in which the globally decreasing temperatures of the Cenozoic were related to the speciation rate of lemurs, but found this model did not explain the data better than models with no environmental effects (electronic supplementary material table S3). As reconstructions of palaeoenvironment and biogeography specific to Madagascar become available [66], it may be possible to clarify if proximal environmental changes were related to the diversification dynamics of lemurs.

One methodological artefact that may drive the observed increasing diversification rates in lemurs is the taxonomic richness recognized. The latest taxonomic assessments suggest there are approximately 100 species [17], while 50 species were recognized only 20 years ago [67]. Some researchers have questioned the validity of the recent taxonomic explosion, suggesting the newly described species may be over-splitting [32]. Many of the newly named species were formerly subspecies or cryptic species that were elevated owing to their level of geographical, genetic and phenotypic differentiation (e.g. *Eulemur* [68], *Microcebus* [69]). Diversification analyses using a phylogeny representing a conservative taxonomy prior to the recent taxonomic revisions [67] suggested constant speciation rates that were half that of analyses with the recent taxonomy (electronic supplementary material, table S5). There is reason to favour the more recent taxonomy, however. While early taxonomic revisions have been criticized, especially for using mitochondrial data only, recent analyses using nuclear genomic level data have supported the deep divergences and genetic structuring among, for example, *Eulemur* [68] and *Microcebus* [70] species. In the lemur phylogenies used in this paper from [26], the mean age of species was 4.53 Ma (median = 2.94, interquartile range = 3.95), and the ages for *Microcebus* species were between 1.15 and 3.84 Ma (interquartile range), with only two species pairs with divergence times less than 1 Ma. The relatively deep divergences, genetic and phenotypic differentiation, and biogeographic separation of species support higher taxonomic richness than was recognized in the past, but the true number of lemurs is still debated.

## 5. Conclusion

Lemurs are a relatively old primate group, characterized by their endemism on Madagascar, their species richness, and the unique ecological niches they occupy. This study tested predictions from the ecological theory of adaptive radiation. Lemur speciation rates were higher than those of their sister group, but there was no evidence of a slowdown in diversification rates. Therefore, based only on diversification dynamics, lemurs may not be considered an adaptive radiation. During the long lemur evolutionary history, extinction may have dampened the signal of past diversification dynamics, and without fossils it is impossible to corroborate the results from phylogenies with hard evidence. Lemur body mass evolution exhibited the early burst pattern, with phenotypic differentiation among lineages that occupy different niches early followed by declining body mass evolutionary rates. From these results, I argue that lemurs fit the phenotypic predictions of an adaptive radiation, but diversification dynamics based on the phylogeny of extant and recently extinct species do not support the predictions of a coincident exceptional burst of speciation.

## Supplementary Material

Supporting methods, data, and results accompanying the manuscript "Testing the adaptive radiation theory for the lemurs of Madagascar"The supplementary materials contain raw data, phylogenetic trees, and further details and clarifications on the methodology and results presented in the main text

## Data accessibility

The raw trait data with references from the literature have been deposited in https://dx.doi.org/10.6084/m9.figshare.c.3655697.v1.

## References

[1] Rabosky DL, Hurlbert AH 2015 Species richness at continental scales is dominated by ecological limits. Am. Nat. 185, 572–583. (doi:10.1086/680850) 25905501 10.1086/680850

[2] Seehausen O 2006 African cichlid fish: a model system in adaptive radiation research. Proc. R. Soc. B 273, 1987–1998. (doi:10.1098/rspb.2006.3539) 10.1098/rspb.2006.3539 PMC1635482 16846905

[3] Schluter D 2000 The ecology of adaptive radiation. Oxford, UK: Oxford University Press.

[4] Glor RE 2010 Phylogenetic insights on adaptive radiation. Annu. Rev. Ecol. Evol. Syst. 41, 251–270. (doi:10.1146/annurev.ecolsys.39.110707.173447)

[5] Gavrilets S, Losos JB 2009 Adaptive radiation: contrasting theory with data. Science 323, 732–737. (doi:10.1126/science.1157966) 19197052 10.1126/science.1157966

[6] Simpson GG 1944 Tempo and mode in evolution. New York, NY: Columbia University Press.

[7] Moen D, Morlon H 2014 Why does diversification slow down? Trends Ecol. Evol. 29, 190–197. (doi:10.1016/j.tree.2014.01.010) 24612774 10.1016/j.tree.2014.01.010

[8] Reddy S, Driskell A, Rabosky D, Hackett S, Schulenberg T 2012 Diversification and the adaptive radiation of the vangas of Madagascar. Proc. R. Soc. B 282, 1–10. (doi:10.1098/rspb.2011.2380) 10.1098/rspb.2011.2380 PMC3311898 22217720

[9] McGuire JA, Witt CC, Remsen J, Corl A, Rabosky DL, Altshuler DL, Dudley R 2014 Molecular phylogenetics and the diversification of hummingbirds. Curr. Biol. 24, 910–916. (doi:10.1016/j.cub.2014.03.016) 24704078 10.1016/j.cub.2014.03.016

[10] Fritz SA, Jønsson KA, Fjeldså J, Rahbek C 2012 Diversification and biogeographic patterns in four island radiations of passerine birds. Evolution 66, 179–190. (doi:10.1111/j.1558-5646.2011.01430.x) 22220873 10.1111/j.1558-5646.2011.01430.x

[11] Ricklefs RE 2010 Evolutionary diversification, coevolution between populations and their antagonists, and the filling of niche space. Proc. Natl Acad. Sci. USA 107, 1265–1272. (doi:10.1073/pnas.0913626107) 20080597 10.1073/pnas.0913626107 PMC2824412

[12] Losos JB 2010 Adaptive radiation, ecological opportunity, and evolutionary determinism. Am. Nat. 175, 623–639. (doi:10.1086/652433) 20412015 10.1086/652433

[13] Grant PR, Grant BR 2006 Evolution of character displacement in Darwin's finches. Science 313, 224–226. (doi:10.1126/science.1128374) 16840700 10.1126/science.1128374

[14] Samonds KE, Godfrey LR, Ali JR, Goodman SM, Vences M, Sutherland MR, Irwin MT, Krause DW 2013 Imperfect isolation: factors and filters shaping Madagascar's extant vertebrate fauna. PLoS ONE 8, e62086 (doi:10.1371/journal.pone.0062086) 23626770 10.1371/journal.pone.0062086 PMC3633922

[15] Martin RD 1972 Adaptive radiation and behaviour of the Malagasy lemurs. Phil. Trans. R. Soc. Lond. B 264, 295–352. (doi:10.1098/rstb.1972.0013) 4403452 10.1098/rstb.1972.0013

[16] Tattersall I 1982 The primates of Madagascar. New York, NY: Columbia University Press.

[17] Mittermeier RA, et al. 2010 Lemurs of Madagascar, 3rd edn, 762 p. Washington, DC: Conservation International.

[18] Godfrey LR, Jungers WL, Burney DA 2010 Subfossil lemurs of Madagascar. In Cenozoic mammals of Africa (eds Werdelin L, Sanders W), pp. 351–367. Berkeley, CA: The University of California Press.

[19] Ganzhorn JU 1988 Food partitioning among Malagasy primates. Oecologia 75, 436–450. (doi:10.1007/bf00376949) 10.1007/BF00376949 28312694

[20] Pozzi L, Disotell TR, Masters JC 2014 A multilocus phylogeny reveals deep lineages within African galagids (Primates: Galagidae). BMC Evol. Biol. 14, 72 (doi:10.1186/1471-2148-14-72) 24694188 10.1186/1471-2148-14-72 PMC4021292

[21] Baab KL, Perry JM, Rohlf FJ, Jungers WL 2014 Phylogenetic, ecological, and allometric correlates of cranial shape in Malagasy lemuriforms. Evolution 68, 1450–1468. (doi:10.1111/evo.12361) 24451053 10.1111/evo.12361

[22] Alfaro ME, Santini F, Brock C, Alamillo H, Dornburg A, Rabosky DL, Carnevale G, Harmon LJ 2009 Nine exceptional radiations plus high turnover explain species diversity in jawed vertebrates. Proc. Natl Acad. Sci. USA 106, 13 410–13 414. (doi:10.1073/pnas.0811087106) 10.1073/pnas.0811087106 PMC2715324 19633192

[23] Rabosky DL 2014 Automatic detection of key innovations, rate shifts, and diversity-dependence on phylogenetic trees. PLoS ONE 9, e89543 (doi:10.1371/journal.pone.0089543) 24586858 10.1371/journal.pone.0089543 PMC3935878

[24] Morlon H 2014 Phylogenetic approaches for studying diversification. Ecol. Lett. 17, 508–525. (doi:10.1111/ele.12251) 24533923 10.1111/ele.12251

[25] Heath TA, Huelsenbeck JP, Stadler T 2014 The fossilized birth-death process for coherent calibration of divergence-time estimates. Proc. Natl Acad. Sci. USA 111, E2957–E2966. (doi:10.1073/pnas.1319091111) 25009181 10.1073/pnas.1319091111 PMC4115571

[26] Herrera JP, Dávalos L 2016 Phylogeny and divergence times of lemurs inferred with recent and ancient fossils in the tree. Syst. Biol. 65, 772–791. (doi:10.1093/sysbio/syw035) 27113475 10.1093/sysbio/syw035

[27] Zachos JC, Dickens GR, Zeebe RE 2008 An early Cenozoic perspective on greenhouse warming and carbon-cycle dynamics. Nature 451, 279–283. (doi:10.1038/nature06588) 18202643 10.1038/nature06588

[28] Seiffert ER 2007 Evolution and extinction of Afro-Arabian primates near the Eocene-Oligocene boundary. Folia Primatol. 78, 314–327. (doi:10.1159/000105147) 17855785 10.1159/000105147

[29] Nunn CL 2011 The comparative approach in evolutionary anthropology and biology. Chicago, IL: University of Chicago Press.

[30] Ronquist F et al. 2012 MrBayes 3.2: efficient Bayesian phylogenetic inference and model choice across a large model space. Syst. Biol. 61, 539–542. (doi:10.1093/sysbio/sys029) 22357727 10.1093/sysbio/sys029 PMC3329765

[31] Kistler L et al. 2015 Comparative and population mitogenomic analyses of Madagascar's extinct, giant ‘subfossil'lemurs. J. Hum. Evol. 79, 45–54. (doi:10.1016/j.jhevol.2014.06.016) 25523037 10.1016/j.jhevol.2014.06.016

[32] Tattersall I 2007 Madagascar's lemurs: cryptic diversity or taxonomic inflation? Evol. Anthropol. 16, 12–23. (doi:10.1002/evan.20126)

[33] Moore BR, Höhna S, May MR, Rannala B, Huelsenbeck JP 2016 Critically evaluating the theory and performance of Bayesian analysis of macroevolutionary mixtures. Proc. Natl Acad. Sci. USA 113, 9569–9574. (doi:10.1073/pnas.1518659113) 27512038 10.1073/pnas.1518659113 PMC5003228

[34] Rabosky DL 2016 Is the BAMM likelihood correct? See http://bamm-project.org/likelihoodmodel.html&hash;is-the-bamm-likelihood-correct. Accessed Aug. 11, 2016

[35] Mitchell JS, Rabosky DL In press. Bayesian model selection with BAMM: effects of the model prior on the inferred number of diversification shifts. Methods Ecol. Evol. (doi:10.1111/2041-210X.12626)

[36] Pennell MW, Eastman JM, Slater GJ, Brown JW, Uyeda JC, FitzJohn RG, Alfaro ME, Harmon LJ 2014 geiger v2. 0: an expanded suite of methods for fitting macroevolutionary models to phylogenetic trees. Bioinformatics 30, 2216–2218. 24728855 10.1093/bioinformatics/btu181

[37] R Core Team. 2014 R: a language and environment for statistical computing. Vienna, Austria: R Foundation for Statistical Computing See http://www.R-project.org/

[38] May MR, Moore BR 2014 How well can we detect shifts in rates of lineage diversification? A simulation study of sequential AIC methods. *BioRxiv*, 011452.

[39] Höhna S, May MR, Moore BR 2015 TESS: an R package for efficiently simulating phylogenetic trees and performing Bayesian inference of lineage diversification rates. Bioinformatics 32, 789–791. (doi:10.1093/bioinformatics/btv651) 26543171 10.1093/bioinformatics/btv651

[40] Morlon H, Potts MD, Plotkin JB 2010 Inferring the dynamics of diversification: a coalescent approach. PLoS Biol. 8, e1000493 (doi:10.1371/journal.pbio.1000493) 20927410 10.1371/journal.pbio.1000493 PMC2946937

[41] Condamine FL, Rolland J, Morlon H 2013 Macroevolutionary perspectives to environmental change. Ecol. Lett. 16, 72–85. (doi:10.1111/ele.12062) 10.1111/ele.12062 23331627

[42] Morlon H, Lewitus E, Condamine F, Manceau M, Clavel J, Drury J 2015 RPANDA: an R package for macroevolutionary analyses on phylogenetic trees. Methods Ecol. Evol. 7, 589–597. (doi:10.1111/2041-210X.12526)

[43] Burnham KP, Anderson DR 2002 Model selection and multimodel inference: a practical information-theoretic approach. New York, NY: Springer.

[44] Nyakatura K, Bininda-Emonds OR 2012 Updating the evolutionary history of Carnivora (Mammalia): a new species-level supertree complete with divergence time estimates. BMC Biol. 10, 12 (doi:10.1186/1741-7007-10-12) 22369503 10.1186/1741-7007-10-12 PMC3307490

[45] Pinheiro J, Bates D, DebRoy S, Sarkar D 2011 nlme: linear and nonlinear mixed effects models. R package version 3.1-97. Vienna, Austria: R Foundation for Statistical Computing (CRAN).

[46] Paradis E, Claude J, Strimmer K 2004 APE: analysis of phylogenetics and evolution in R language. Bioinformatics 20, 289–290. (doi:10.1093/bioinformatics/btg412) 14734327 10.1093/bioinformatics/btg412

[47] Pennell M, FitzJohn RG, Cornwell WK, Harmon LJ 2014 Model adequacy and the macroevolution of angiosperm functional traits. BioRxiv, 004002. 10.1086/682022 26655160

[48] Beaulieu JM, Jhwueng DC, Boettiger C, O'Meara BC 2012 Modeling stabilizing selection: expanding the Ornstein–Uhlenbeck model of adaptive evolution. Evolution 66, 2369–2383. (doi:10.1111/j.1558-5646.2012.01619.x) 22834738 10.1111/j.1558-5646.2012.01619.x

[49] Huelsenbeck JP, Nielsen R, Bollback JP 2003 Stochastic mapping of morphological characters. Syst. Biol. 52, 131–158. (doi:10.1080/10635150390192780) 12746144 10.1080/10635150390192780

[50] Revell L 2015 phytools v0.4-45: phylogenetic tools for comparative biology (and other things). (CRAN).

[51] Maechler M, Rousseeuw P, Struyf A, Hubert M, Hornik K, Studer M, Roudier P 2015 Cluster analysis extended Rousseeuw *et al*. (CRAN).

[52] Oksanen J et al. 2013 Package ‘vegan’. R Package ver 254, 20–28.

[53] Harmon LJ et al. 2010 Early bursts of body size and shape evolution are rare in comparative data. Evolution 64, 2385–2396. (doi:10.1111/j.1558-5646.2010.01025.x) 20455932 10.1111/j.1558-5646.2010.01025.x

[54] Slater GJ, Price SA, Santini F, Alfaro ME 2010 Diversity versus disparity and the radiation of modern cetaceans. Proc. R. Soc. B 277, 3097–3104. (doi:10.1098/rspb.2010.0408) 10.1098/rspb.2010.0408 PMC2982053 20484243

[55] Price SA, Hopkins SS 2015 The macroevolutionary relationship between diet and body mass across mammals. Biol. J. Linn. Soc. 115, 173–184. (doi:10.1111/bij.12495)

[56] Kay RF 1975 The functional adaptations of primate molar teeth. Am. J. Phys. Anthropol. 43, 195–215. (doi:10.1002/ajpa.1330430207) 810034 10.1002/ajpa.1330430207

[57] Godfrey LR, Winchester JM, King SJ, Boyer DM, Jernvall J 2012 Dental topography indicates ecological contraction of lemur communities. Am. J. Phys. Anthropol. 148, 215–227. (doi:10.1002/ajpa.21615) 22610897 10.1002/ajpa.21615

[58] Perrin MR 2013 The gastrointestinal anatomy of the lesser bamboo lemur, *Hapalemur griseus*, with comments on digestive function. S. Afr. J. Wildl. Res. 43, 79–83. (doi:10.3957/056.043.0112)

[59] Prothero DR 1994 The late Eocene-Oligocene extinctions. Annu. Rev. Earth Planet. Sci. 22, 145–165. (doi:10.1146/annurev.ea.22.050194.001045)

[60] Rabosky DL, Glor RE 2010 Equilibrium speciation dynamics in a model adaptive radiation of island lizards. Proc. Natl Acad. Sci. USA 107, 22 178–22 183. (doi:10.1073/pnas.1007606107) 21135239 10.1073/pnas.1007606107 PMC3009809

[61] Brock CD, Harmon LJ, Alfaro ME 2011 Testing for temporal variation in diversification rates when sampling is incomplete and nonrandom. Syst. Biol. 60, 410–419. (doi:10.1093/sysbio/syr007) 21378083 10.1093/sysbio/syr007

[62] Harmon LJ, Harrison S 2015 Species diversity is dynamic and unbounded at local and continental scales. Am. Nat. 185, 584–593. (doi:10.1086/680859) 25905502 10.1086/680859

[63] Liow LH, Van Valen L, Stenseth NC 2011 Red Queen: from populations to taxa and communities. Trends Ecol. Evol. 26, 349–358. (doi:10.1016/j.tree.2011.03.016) 21511358 10.1016/j.tree.2011.03.016

[64] Coyne JA, Orr HA 2004 *Speciation* Sunderland, MA: Sinauer Associates.

[65] Lovette IJ, Bermingham E 1999 Explosive speciation in the New World *Dendroica* warblers. Proc. R. Soc. Lond. B 266, 1629–1636. (doi:10.1098/rspb.1999.0825)

[66] Ohba M, Samonds KE, LaFleur M, Ali JR, Godfrey LR 2016 Madagascar's climate at the K/P boundary and its impact on the island's biotic suite. Palaeogeogr. Palaeoclimatol. Palaeoecol. 441, 688–695. (doi:10.1016/j.palaeo.2015.10.028)

[67] Ganzhorn JU, Goodman SM, Nash S, Thalmann U 2006 Lemur biogeography. In Primate biogeography: progress and perspectives (eds Fleagle J, Lehman SM), pp. 229--254 New York, NY: Springer.

[68] Markolf M, Rakotonirina H, Fichtel C, von Grumbkow P, Brameier M, Kappeler PM 2013 True lemurs … true species-species delimitation using multiple data sources in the brown lemur complex. BMC Evol. Biol. 13, 233 (doi:10.1186/1471-2148-13-233) 24159931 10.1186/1471-2148-13-233 PMC3819746

[69] Weisrock DW, Rasoloarison RM, Fiorentino I, Ralison JM, Goodman SM, Kappeler PM, Yoder AD 2010 Delimiting species without nuclear monophyly in Madagascar's mouse lemurs. PLoS ONE 5, e9883 (doi:10.1371/journal.pone.0009883) 20360988 10.1371/journal.pone.0009883 PMC2847600

[70] Yoder AD et al. 2016 Geogenetic patterns in mouse lemurs (genus *Microcebus*) reveal the ghosts of Madagascar's forests past. Proc. Natl Acad. Sci. USA 113, 8049–8056. (doi:10.1073/pnas.1601081113) 27432945 10.1073/pnas.1601081113 PMC4961119

