# Supplementary material for: Testing the adaptive radiation hypothesis for the lemurs of Madagascar
Source: R Soc Open Sci. 2017 Jan 18;4(1):161014. doi: 10.1098/rsos.161014 (PMC5319363; doi:10.1098/rsos.161014)
Supplement: Supporting methods, data, and results accompanying the manuscript "Testing the adaptive radiation theory for the lemurs of Madagascar"The supplementary materials contain raw data, phylogenetic trees, and further details and clarifications on the methodology and results presented in the main text [file rsos161014supp1.docx]

**Electronic Supplementary Materials**

**Materials and Methods**

**Testing for adaptive radiation:** To test if lemurs diversified via an adaptive radiation, my analyses aim to 1) estimate the lineage-specific speciation and extinction rates to quantify net diversification and test for a shift in diversification rates through time and compared to their sister clade, 2) estimate the rate of phenotypic evolution through time and test for shifts in phenotypic evolution through time and across the tree, 3) test the link between body mass (phenotypic trait), diet and activity pattern (adaptive zones) and test for different phenotypic optima in different niche regimes.

**Taxonomic sampling and phylogeny**: The taxonomy of lemurs has changed recently with the use of available molecular evidence to suggest many cryptic species that were previously subsumed as single species. I recently inferred a near-complete phylogeny that includes 87 living lemurs (87.9% out of 99 recognized taxa[1, 2]) and 14 extinct subfossils (82.4%[3]). I compared speciation and phenotypic evolutionary rates between lemurs and extant lorisiforms (lorises and galagos) of Africa and Asia (67.9%, 19 / 28 IUCN recognized species). I accounted for incomplete species sampling in lineage diversification analyses by specifying the proportion of sampled species for each genus (Electronic Supplementary Materials, SM, Table S1).

**Table S1.** Proportion of sampled species per genus of lemurs or per lineage of lorisiforms. Note that in the tree from ref. [8], not all genera were represented in the tree, and sampling fractions for missing lineages were assigned to the next closest clade (see footnotes).

| Genus | Proportion of total species in tree ref. [3] | Proportion of species in tree ref. [8] |
| --- | --- | --- |
| *Propithecus* | 1 | 0.33 |
| *Avahi* | 0.89 | 0.11 |
| *Palaeopropithecus* | 0.67 | 0.25^1^ |
| *Archaeoindris* | 1 | 0^1^ |
| *Mesopropithecus* | 0.67 | 0^1^ |
| *Babakotia* | 1 | 0^1^ |
| *Indri* | 1 | 1 |
| *Archaeolemur* | 1 | 0^2^ |
| *Hadropithecus* | 1 | 0.33^2^ |
| *Eulemur* | 1 | 0.17 |
| *Hapalemur* | 1 | 0^3^ |
| *Lemur* | 1 | 0.14^3^ |
| *Varecia* | 1 | 0.5 |
| *Pachylemur* | 0.5 | 0.5 |
| *Microcebus* | 1 | 0^4^ |
| *Mirza* | 1 | 0.03^4^ |
| *Allocebus* | 1 | 0^4^ |
| *Cheirogaleus* | 1 | 0^4^ |
| *Phaner* | 0.25 | 0^4^ |
| *Lepilemur* | 0.58 | 0.08 |
| *Megaladapis* | 1 | 0.33 |
| *Daubentonia* | 1 | 0.5 |
| Galagids | 0.53 | 0.3 |
| Lorisids | 0.57 | 0.3 |

1. *Palaeopropithecus* represents Palaeopropithecidae, 2. *Hadropithecus* represents Archaeolemuridae, 3. *Lemur* represents *Lemur* + *Hapalemur*, 4. *Mirza* represents Cheirogaleidae.

**Phylogenies:** In the lineage diversification rate analyses described below (Bayesian analysis of macroevolutionary mixtures[4], TESS [5], RPANDA [6], MEDUSA [7]), I used the maximum clade credibility (MCC) phylogeny inferred from total evidence analysis under the fossilized birth-death process model [3] (File S1), as well as four alternative trees, including three variants of the MCC tree, to evaluate if the results are robust to changes in the phylogenetic relationships. 1) An alternate topological hypothesis with the genus *Megaladapis* sister to the family Lemuridae, as supported by recent studies of ancient mitochondrial DNA [8]. I extracted and grafted *Megaladapis* with the placement and the estimated divergence times proposed from previous studies [8] (File S2). 2) The MCC tree of only extant lemurs to evaluate the effects of observed extinctions on the estimates of diversification dynamics (File S3). 3) To ascertain the effects of the recent taxonomic splitting of lemur species on inferences of diversification dynamics, I also inferred speciation, extinction, and trait evolutionary rates using a tree pruned to reflect the taxonomy before 2006 [9], which recognized 51 lemurs including extinct subfossil taxa and 10 lorisiforms (File S4). 4) The phylogeny of lemurs based on mitochondrial genomes, including several of the extinct genera [8] (File S5). Further, to evaluate the effects of variation in topology and branch lengths, I ran RPANDA and MEDUSA diversification analyses, and phenotypic evolution analyses over a subset of 100 trees from the posterior distribution of trees from the total evidence analysis.

**Trait data:** In testing for adaptive patterns of niche evolution, it is necessary to identify ecologically relevant traits[10]. I chose body mass as a phenotypic trait related to discrete adaptive zones of diet and activity pattern. Body mass is well known to be related to many aspects of a species’ physiology, life history, and ecology, thus encompassing much of the biology of the organism[11]. Body mass is tightly correlated to diet, but there are many adaptations to diets beyond changes in body mass (e.g., gut morphology, resource extraction strategy) that lead to deviations from the correlation of diet and body mass. Diet and activity pattern are important variables defining a species’ niche, with many morphological and physiological correlates, and which influence interspecies competition.

To gather the phenotypic and niche data, I searched primary literature sources. I obtained body mass estimates for 119 species, and despite the species sampling being incomplete, body mass data were obtained for species representing the full range of variation for lemurs (smallest: *Microcebus berthae* = 30 g, largest extant: *Indri indri* = 6500 g, largest extinct: *Archaeoindris fontoynontii* = 161200 g) and lorisiforms (smallest: *Galagoides demidoff* = 61 g, largest: *Otolemur crassicaudatus* 1150 g). The body mass data with references are provided in Table S2. Body mass was natural log transformed for further analyses. All taxa were grouped into two dietary categories: folivore or omnivore. Folivores were species whose primary dietary adaptations are for consuming leaves, with leaves typically making up >50% of the diet (e.g., species with sharp and sheering crests on molars, enlarged caecum for hind-gut fermentation). Omnivores were species whose primary diet included a combination of fruit, insects, exudates, and/or nectar. These coarse categories do not reflect the proportions of food types in the diet; some species eat a considerable amount of fruit or seeds (*Propithecus* species), but their primary dietary adaptations and highest proportion of dietary intake is folivory; thus they were coded as folivores. These data were taken from the literature and grouped based on interpretations of the dietary niches of the organisms. For extinct taxa, the morphology of the dentition has been the primary source of dietary inference[12]. I categorized species as either diurnal or nocturnal based on their primary activity period. I grouped cathemeral species into the diurnal category because within lemurs, cathemerality has only evolved in one clade, Lemuridae, documented primarily in *Eulemur* and *Lemur catta*, with anecdotal evidence of some nocturnal activity in other lemurids[13, 14]. This limits the power of comparative analyses to test patterns related to cathemerality[15]. Further, while some lemurs are known to be cathemeral with frequent bouts of activity at night, their predominant activity is diurnal, and for many species their cathemeral activities are seasonal, related to moonlight, or contentious[16]. That being said, in a broader phylogenetic context (all primates), a relationship was shown between activity pattern (including cathemerality), and diversification rates[17]. Finally, each taxon was assigned a categorical niche state: nocturnal omnivore, diurnal omnivore, nocturnal folivore, diurnal folivore.

**SI Table S2.** Body mass and niche data taken from the literature with references.

| ***Binomial*** | **Activity** | **Diet** | **Mass (grams)** | **Mass reference** | **Diet reference** | **Activity reference** |
| --- | --- | --- | --- | --- | --- | --- |
| *Allocebus trichotis* | nocturnal | omnivore | 77.50 | Biebouw 2009 | Biebouw 2009 | Donati and Borgognini-Tarli 2006 |
| *Archaeoindris fontoynontii* | diurnal | folivore | 161200.00 | Jungers et al. 2008 | Godfrey et al. 2012 | Godfrey et al. 2010 |
| *Archaeolemur edwardsi* | diurnal | frugivore | 26500.00 | Jungers et al. 2008 | Godfrey et al. 2012 | Godfrey et al. 2010 |
| *Archaeolemur majori* | diurnal | frugivore | 18200.00 | Jungers et al. 2008 | Godfrey et al. 2012 | Godfrey et al. 2010 |
| *Avahi betsileo* | nocturnal | folivore | 1050.00 | Andriantompohavana et al. 2007 | Andriantompohavana et al. 2007 | Mittermeier et al. 2010 |
| *Avahi cleesei* | nocturnal | folivore | 1160.66 | Thalman and Geissmann 2005; Zaramody et al. 2006 | Mittermeier et al. 2010 | Donati and Borgognini-Tarli 2006 |
| *Avahi laniger* | nocturnal | folivore | 1161.65 | Zaramody et al. 2006 | Harcourt 1991; Faulkner and Lehman 2006; Ganzhorn 1985 | Donati and Borgognini-Tarli 2006 |
| *Avahi meridionalis* | nocturnal | folivore | 806.73 | Zaramody et al. 2007 | Norscia et al. 2012 | Donati and Borgognini-Tarli 2006 |
| *Avahi mooreorum* | nocturnal | folivore | 924.00 | Andriantompohavana et al. 2007 | Mittermeier et al. 2010 | Mittermeier et al. 2010 |
| *Avahi occidentalis* | nocturnal | folivore | 1038.07 | Thalman and Geissmann 2000 | Thalmann 2001 | Donati and Borgognini-Tarli 2006 |
| *Avahi peyrierasi* | nocturnal | folivore | 993.50 | Andriantompohavana et al. 2007 | Andriantompohavana et al. 2007 | Mittermeier et al. 2010 |
| *Avahi ramanantsoavanai* | nocturnal | folivore | 1050.00 | Zaramody et al. 2007 | Mittermeier et al. 2010 | Donati and Borgognini-Tarli 2006 |
| *Avahi unicolor* | nocturnal | folivore | 840.00 | Andriantompohavana et al. 2007 | Mittermeier et al. 2010 | Mittermeier et al. 2010 |
| *Babakotia radafolia* | diurnal | folivore | 20700.00 | Jungers et al. 2008 | Godfrey et al. 2012 | Godfrey et al. 2010 |
| *Cheirogaleus crossleyi* | nocturnal | omnivore | 395.45 | Blanco et al. 2009; this study | Wright and Martin 1995 | Donati and Borgognini-Tarli 2006 |
| *Cheirogaleus lavasoaensis* | nocturnal | omnivore | 297.00 | Thiele et al. 2013 | Thiele et al. 2013 | Thiele et al. 2013 |
| *Cheirogaleus major* | nocturnal | omnivore | 317.00 | Fietz, 2003 | Mittermeier et al. 2010 | Donati and Borgognini-Tarli 2006 |
| *Cheirogaleus medius* | nocturnal | omnivore | 241.17 | Fietz and Ganzhorn 1999 | Fietz 2003 | Donati and Borgognini-Tarli 2006 |
| *Cheirogaleus sibreei* | nocturnal | omnivore | 243.94 | Blanco et al. 2009; this study | pers. Obs. JPH | Donati and Borgognini-Tarli 2006 |
| *Daubentonia madagascariensis* | nocturnal | omnivore | 2675.00 | Sterling 1993 | Sterling 1994 | Donati and Borgognini-Tarli 2006 |
| *Daubentonia robustus* |  | omnivore | 14200.00 | Jungers et al. 2008 | Godfrey et al. 2004 | Donati and Borgognini-Tarli 2006 |
| *Eulemur albifrons* | diurnal | frugivore | 2310.00 | Junge et al. 2009 | Johnson 2007 | Curtis 2006 |
| *Eulemur cinereiceps* | diurnal | frugivore | 2250.00 | Johnson et al. 2005; Mittermeier et al. 2010 | Johnson et al. 2005; Mittermeier et al. 2010 | Donati and Borgognini-Tarli 2006 |
| *Eulemur collaris* | diurnal | frugivore | 2180.00 | Donati et al., 2007 | Johnson 2007 | Donati and Borgognini-Tarli 2006 |
| *Eulemur coronatus* | diurnal | frugivore | 1200.00 | Terranova and Coffman, 1997 | Johnson 2007 | Donati and Borgognini-Tarli 2006 |
| *Eulemur flavifrons* | diurnal | frugivore | 1900.00 | Terranova and Coffman, 1997 | Johnson 2007 | Schwitzer et al. 2007 |
| *Eulemur fulvus* | diurnal | frugivore | 1820.00 | Rassmussen 1999 | Johnson 2007 | Donati and Borgognini-Tarli 2006 |
| *Eulemur macaco* | diurnal | frugivore | 1820.00 | Colquhoun 1997 | Johnson 2007 | Donati and Borgognini-Tarli 2006 |
| *Eulemur mongoz* | diurnal | frugivore | 1043.00 | Rasmussen 1999 | Johnson 2007 | Donati and Borgognini-Tarli 2006 |
| *Eulemur rubriventer* | diurnal | frugivore | 2013.50 | Glander et al. 1992 | Johnson 2007 | Donati and Borgognini-Tarli 2006 |
| *Eulemur rufifrons* | diurnal | frugivore | 2188.84 | Glander et al. 1992 | Johnson 2007 | Donati and Borgognini-Tarli 2006 |
| *Eulemur rufus* | diurnal | frugivore | 1828.98 | Junge et al. 2005 | Johnson 2007 | Donati and Borgognini-Tarli 2006 |
| *Eulemur sanfordi* | diurnal | frugivore | 1850.00 | Terranova and Coffman, 1997 | Johnson 2007 | Donati and Borgognini-Tarli 2006 |
| *Hadropithecus stenognathus* | diurnal | folivore | 35400.00 | Jungers et al. 2008 | Godfrey et al. 2012 | Godfrey et al. 2010 |
| *Hapalemur alaotrensis* | diurnal | folivore | 1530.41 | Mittermeier et al., 2010 | Tan 2000 | Mutschler et al. 1998 |
| *Hapalemur aureus* | diurnal | folivore | 1548.00 | Glander et al. 1992 | Tan 2000 | Tan 2000 |
| *Hapalemur griseus* | diurnal | folivore | 935.00 | Glander et al. 1992 | Tan 2000 | Tan 2000 |
| *Hapalemur meridionalis* | diurnal | folivore | 855.00 | Mittermeier et al., 2010 | Tan 2000 | Tan 2006 |
| *Hapalemur occidentalis* | diurnal | folivore | 900.00 | Mittermeier et al., 2010 | Tan 2000 | Tan 2006 |
| *Hapalemur simus* | diurnal | folivore | 2450.00 | Tan 1999 | Tan 2000 | Tan 2000 |
| *Indri indri* | diurnal | folivore | 6500.00 | Glander and Powzyk 1998 | Godfrey et al. 2012 | Donati and Borgognini-Tarli 2006 |
| *Lemur catta* | diurnal | frugivore | 2245.00 | Koyama 2008 | Godfrey et al. 2012 | Donati et al. 2013 |
| *Lepilemur aeeclis* | nocturnal | folivore | 988.64 | Louis et al. 2006 | Mittermeier et al. 2010 | Donati and Borgognini-Tarli 2006 |
| *Lepilemur ahmansonorum* | nocturnal | folivore | 610.00 | Louis et al. 2006 | Mittermeier et al. 2010 | Mittermeier et al. 2010 |
| *Lepilemur ankaranensis* | nocturnal | folivore | 608.01 | Louis et al. 2006 | Mittermeier et al. 2010 | Donati and Borgognini-Tarli 2006 |
| *Lepilemur betsileo* | nocturnal | folivore | 1150.00 | Louis et al. 2006 | Mittermeier et al. 2010 | Mittermeier et al. 2010 |
| *Lepilemur dorsalis* | nocturnal | folivore | 1087.50 | Louis et al. 2006 | Mittermeier et al. 2010 | Donati and Borgognini-Tarli 2006 |
| *Lepilemur edwardsi* | nocturnal | folivore | 978.75 | Louis et al. 2006 | Thalmann 2001 | Donati and Borgognini-Tarli 2006 |
| *Lepilemur fleuretae* | nocturnal | folivore | 980.00 | Louis et al. 2006 | Mittermeier et al. 2010 | Mittermeier et al. 2010 |
| *Lepilemur grewcockorum* | nocturnal | folivore | 780.00 | Louis et al. 2006 | Mittermeier et al. 2010 | Mittermeier et al. 2010 |
| *Lepilemur hollandorum* | nocturnal | folivore | 1000.00 | Louis et al. 2006 | Mittermeier et al. 2010 | Mittermeier et al. 2010 |
| *Lepilemur hubbardorum* | nocturnal | folivore | 771.14 | Louis et al. 2006 | Mittermeier et al. 2010 | Donati and Borgognini-Tarli 2006 |
| *Lepilemur jamesorum* | nocturnal | folivore | 780.00 | Louis et al. 2006 | Mittermeier et al. 2010 | Mittermeier et al. 2010 |
| *Lepilemur leucopus* | nocturnal | folivore | 771.14 | Louis et al. 2006 | Mittermeier et al. 2010 | Donati and Borgognini-Tarli 2006 |
| *Lepilemur microdon* | nocturnal | folivore | 721.71 | Louis et al. 2006 | Mittermeier et al. 2010 | Donati and Borgognini-Tarli 2006 |
| *Lepilemur milanoii* | nocturnal | folivore | 720.00 | Louis et al. 2006 | Mittermeier et al. 2010 | Mittermeier et al. 2010 |
| *Lepilemur mittermeieri* | nocturnal | folivore | 988.64 | Louis et al. 2006 | Mittermeier et al. 2010 | Donati and Borgognini-Tarli 2006 |
| *Lepilemur mustelinus* | nocturnal | folivore | 852.70 | Louis et al. 2006 | Mittermeier et al. 2010 | Donati and Borgognini-Tarli 2006 |
| *Lepilemur otto* | nocturnal | folivore | 779.05 | Louis et al. 2006 | Mittermeier et al. 2010 | Donati and Borgognini-Tarli 2006 |
| *Lepilemur petteri* | nocturnal | folivore | 630.00 | Louis et al. 2006 | Mittermeier et al. 2010 | Mittermeier et al. 2010 |
| *Lepilemur randrianasoloi* | nocturnal | folivore | 920.00 | Louis et al. 2006 | Mittermeier et al. 2010 | Mittermeier et al. 2010 |
| *Lepilemur ruficaudatus* | nocturnal | folivore | 692.05 | Louis et al. 2006 | Ganzhorn 2002 | Donati and Borgognini-Tarli 2006 |
| *Lepilemur sahamalazensis* | nocturnal | folivore | 741.48 | Louis et al. 2006 | Mittermeier et al. 2010 | Donati and Borgognini-Tarli 2006 |
| *Lepilemur scottorum* | nocturnal | folivore | 876.00 | Louis et al. 2006 | Mittermeier et al. 2010 | Mittermeier et al. 2010 |
| *Lepilemur seali* | nocturnal | folivore | 961.00 | Lei et al. 2007 | Mittermeier et al. 2010 | Mittermeier et al. 2010 |
| *Lepilemur septentrionalis* | nocturnal | folivore | 580.00 | Louis et al. 2006 | Mittermeier et al. 2010 | Donati and Borgognini-Tarli 2006 |
| *Lepilemur tymerlachsoni* | nocturnal | folivore | 875.00 | Louis et al. 2006 | Mittermeier et al. 2010 | Mittermeier et al. 2010 |
| *Lepilemur wrightae* | nocturnal | folivore | 1150.00 | Louis et al. 2006 | Mittermeier et al. 2010 | Mittermeier et al. 2010 |
| *Megaladapis edwardsi* | diurnal | folivore | 85100.00 | Jungers et al. 2008 | Godfrey et al. 2012 | Godfrey et al. 2010 |
| *Megaladapis grandidieri* | diurnal | folivore | 74300.00 | Jungers et al. 2008 | Godfrey et al. 2004 | Godfrey et al. 2010 |
| *Megaladapis madagascariensis* | diurnal | folivore | 46500.00 | Jungers et al. 2008 | Godfrey et al. 2004 | Godfrey et al. 2010 |
| *Mesopropithecus dolichobrachion* | diurnal | folivore | 13700.00 | Jungers et al. 2009 | Godfrey et al. 2012 | Godfrey et al. 2010 |
| *Mesopropithecus pithecoides* | diurnal | folivore | 11300.00 | Jungers et al. 2010 | Godfrey et al. 2012 | Godfrey et al. 2010 |
| *Microcebus arnholdi* | nocturnal | omnivore | 50.00 | Louis et al. 2008 | Mittermeier et al. 2010 | Donati and Borgognini-Tarli 2006 |
| *Microcebus berthae* | nocturnal | omnivore | 30.60 | Louis et al. 2006 | Mittermeier et al. 2010 | Donati and Borgognini-Tarli 2006 |
| *Microcebus bongolavensis* | nocturnal | omnivore | 55.00 | Mittermeier et al., 2010 | Mittermeier et al. 2010 | Donati and Borgognini-Tarli 2006 |
| *Microcebus danfossi* | nocturnal | omnivore | 63.00 | Olivieri et al. 2007 | Mittermeier et al. 2010 | Donati and Borgognini-Tarli 2006 |
| *Microcebus gerpi* | nocturnal | omnivore | 70.00 | Radespiel et al. 2012 | Mittermeier et al. 2010 | Donati and Borgognini-Tarli 2006 |
| *Microcebus griseorufus* | nocturnal | omnivore | 62.60 | Yoder et al. 2000 | Mittermeier et al. 2010 | Donati and Borgognini-Tarli 2006 |
| *Microcebus jollyae* | nocturnal | omnivore | 61.00 | Louis et al. 2006 | Mittermeier et al. 2010 | Mittermeier et al. 2010 |
| *Microcebus lehilahytsara* | nocturnal | omnivore | 39.00 | Rasoloarison et al. 2013 | Mittermeier et al. 2010 | Donati and Borgognini-Tarli 2006 |
| *Microcebus macarthurii* | nocturnal | omnivore | 53.70 | Radespiel et al. 2012 | Mittermeier et al. 2010 | Donati and Borgognini-Tarli 2006 |
| *Microcebus mamiratra* | nocturnal | omnivore | 57.80 | Radespiel et al. 2012 | Mittermeier et al. 2010 | Donati and Borgognini-Tarli 2006 |
| *Microcebus margotmarshae* | nocturnal | omnivore | 41.00 | Louis et al. 2008 | Mittermeier et al. 2010 | Mittermeier et al. 2010 |
| *Microcebus marohita* | nocturnal | omnivore | 55.76 | Rasoloarison et al. 2013 | Mittermeier et al. 2010 | Donati and Borgognini-Tarli 2006 |
| *Microcebus mittermeieri* | nocturnal | omnivore | 59.32 | Louis et al. 2006 | Mittermeier et al. 2010 | Donati and Borgognini-Tarli 2006 |
| *Microcebus murinus* | nocturnal | omnivore | 48.44 | Schmid and Kappeler 1998 | Schmid and Kappeler 1998 | Donati and Borgognini-Tarli 2006 |
| *Microcebus myoxinus* | nocturnal | omnivore | 58.33 | Yoder et al. 2000 | Mittermeier et al. 2010 | Donati and Borgognini-Tarli 2006 |
| *Microcebus ravelobensis* | nocturnal | omnivore | 42.51 | Randrianambinina et al. 2003 | Mittermeier et al. 2010 | Donati and Borgognini-Tarli 2006 |
| *Microcebus rufus* | nocturnal | omnivore | 43.60 | Louis et al. 2006 | Atsalis 2007 | Donati and Borgognini-Tarli 2006 |
| *Microcebus sambiranensis* | nocturnal | omnivore | 59.91 | Yoder et al. 2000 | Mittermeier et al. 2010 | Donati and Borgognini-Tarli 2006 |
| *Microcebus simmonsi* | nocturnal | omnivore | 50.91 | Louis et al. 2006 | Mittermeier et al. 2010 | Donati and Borgognini-Tarli 2006 |
| *Microcebus tavaratra* | nocturnal | omnivore | 61.10 | Yoder et al. 2000 | Mittermeier et al. 2010 | Donati and Borgognini-Tarli 2006 |
| *Mirza coquereli* | nocturnal | omnivore | 289.67 | Ganzhorn and Kappeler 1996 | Ganzhorn and Kappeler 1996 | Donati and Borgognini-Tarli 2006 |
| *Mirza zaza* | nocturnal | omnivore | 300.00 | Kappeler et al. 2005 | Kappeler et al. 2005 | Donati and Borgognini-Tarli 2006 |
| *Pachylemur jullyi* | diurnal | frugivore | 11500.00 | Godfrey et al. 2010 | Godfrey et al. 2010 | Godfrey et al. 2010 |
| *Palaeopropithecus ingens* | diurnal | folivore | 41500.00 | Jungers et al. 2008 | Godfrey et al. 2010 | Godfrey et al. 2010 |
| *Palaeopropithecus maximus* | diurnal | folivore | 45800.00 | Jungers et al. 2008 | Godfrey et al. 2010 | Godfrey et al. 2010 |
| *Phaner electromontis* | nocturnal | omnivore | 387.00 | Mittermeier et al., 2010 | Mittermeier et al. 2010 | Mittermeier et al., 2010 |
| *Phaner furcifer* | nocturnal | omnivore | 450.00 | Ganzhorn and Kappeler 1996 | Ganzhorn and Kappeler 1996 | Donati and Borgognini-Tarli 2006 |
| *Phaner pallescens* | nocturnal | omnivore | 327.00 | Mittermeier et al., 2010 | Mittermeier et al., 2010 | Mittermeier et al., 2010 |
| *Phaner parienti* | nocturnal | omnivore | 360.00 | Mittermeier et al., 2010 | Mittermeier et al., 2010 | Mittermeier et al., 2010 |
| *Propithecus candidus* | diurnal | folivore | 5500.00 | Lehman et al. 2005 | Lehman et al. 2005 | Donati and Borgognini-Tarli 2006 |
| *Propithecus coquereli* | diurnal | folivore | 3700.00 | Kappeler 1991 | Irwin 2007 | Donati and Borgognini-Tarli 2006 |
| *Propithecus coronatus* | diurnal | folivore | 3900.00 | Mittermeier et al., 2010 | Irwin 2007 | Donati and Borgognini-Tarli 2006 |
| *Propithecus deckenii* | diurnal | folivore | 2810.00 | Junge et al. 2005 | Irwin 2007 | Donati and Borgognini-Tarli 2006 |
| *Propithecus diadema* | diurnal | folivore | 5190.00 | Irwin 2006 | Irwin 2006 | Donati and Borgognini-Tarli 2006 |
| *Propithecus edwardsi* | diurnal | folivore | 5742.50 | Glander et al. 1992 | Hemingway 1998 | Donati and Borgognini-Tarli 2006 |
| *Propithecus perrieri* | diurnal | folivore | 4332.50 | Lehman et al. 2005 | Lehman et al. 2005 | Donati and Borgognini-Tarli 2006 |
| *Propithecus tattersalli* | diurnal | folivore | 3500.00 | Meyers and Wright 1993 | Irwin 2007 | Donati and Borgognini-Tarli 2006 |
| *Propithecus verreauxi* | diurnal | folivore | 3190.00 | Lewis and Kappeler 2005 | Irwin 2007 | Donati and Borgognini-Tarli 2006 |
| *Varecia rubra* | diurnal | frugivore | 3470.00 | Vasey 2005 | Vasey 2005 | Donati and Borgognini-Tarli 2006 |
| *Varecia variegata* | diurnal | frugivore | 3665.00 | Baden et al. 2008 | Balko and Underwood 2005 | Donati and Borgognini-Tarli 2006 |
| *Galago moholi* | nocturnal | omnivore | 200.00 | Nekaris and Bearder 2011 | Nekaris and Bearder 2011 | Nekaris and Bearder 2011 |
| *Galago senegalensis* | nocturnal | omnivore | 247.75 | Nekaris and Bearder 2011 | Nekaris and Bearder 2005 | Nekaris and Bearder 2011 |
| *Arctocebus calabarensis* | nocturnal | omnivore | 309.00 | Nekaris and Bearder 2011 | Nekaris and Bearder 2006 | Nekaris and Bearder 2011 |
| *Euoticus elegantulus* | nocturnal | omnivore | 274.00 | Nekaris and Bearder 2011 | Nekaris and Bearder 2007 | Nekaris and Bearder 2011 |
| *Galagoides demidoff* | nocturnal | omnivore | 61.50 | Nekaris and Bearder 2011 | Nekaris and Bearder 2008 | Nekaris and Bearder 2011 |
| *Otolemur crassicaudatus* | nocturnal | omnivore | 1150.00 | Nekaris and Bearder 2011 | Nekaris and Bearder 2009 | Nekaris and Bearder 2011 |
| *Otolemur garnetti* | nocturnal | omnivore | 764.00 | Nekaris and Bearder 2011 | Nekaris and Bearder 2010 | Nekaris and Bearder 2011 |
| *Perodicticus potto* | nocturnal | omnivore | 1000.00 | Nekaris and Bearder 2011 | Nekaris and Bearder 2011 | Nekaris and Bearder 2011 |
| *Nycticebus coucang* | nocturnal | omnivore | 652.50 | Nekaris and Bearder 2011 | Nekaris and Bearder 2012 | Nekaris and Bearder 2011 |
| *Nycticebus bengalensis* | nocturnal | omnivore | 1270.00 | Nekaris and Bearder 2011 | Nekaris and Bearder 2011 | Nekaris and Bearder 2011 |
| *Nycticebus pygmaeus* | nocturnal | omnivore | 307.00 | Nekaris and Bearder 2011 | Nekaris and Bearder 2012 | Nekaris and Bearder 2011 |
| *Nycticebus javanicus* | nocturnal | omnivore | 700.00 | Nekaris et al. 2014 | Nekaris et al. 2014 | Nekaris et al. 2014 |
| *Nycticebus menagensis* | nocturnal | omnivore | 532.50 | Nekaris and Bearder 2011 | Nekaris and Bearder 2011 | Nekaris and Bearder 2011 |
| *Loris tardigradus* | nocturnal | omnivore | 229.50 | Nekaris and Bearder 2011 | Nekaris and Bearder 2012 | Nekaris and Bearder 2011 |
| *Galago thomasi* | nocturnal | omnivore | 78.00 | Nekaris and Bearder 2011 | Nekaris and Bearder 2011 | Nekaris and Bearder 2011 |
| *Galago alleni* | nocturnal | omnivore | 439.33 | Nekaris and Bearder 2011 | Nekaris and Bearder 2011 | Nekaris and Bearder 2011 |
| *Galagoides zanzibaricus* | nocturnal | omnivore | 149.00 | Nekaris and Bearder 2011 | Nekaris and Bearder 2011 | Nekaris and Bearder 2011 |
| *Galagoides cocos* | nocturnal | omnivore | 150.00 | Nekaris and Bearder 2011 | Nekaris and Bearder 2011 | Nekaris and Bearder 2011 |
| *Galago matschiei* | nocturnal | omnivore | 210.50 | Nekaris and Bearder 2011 | Nekaris and Bearder 2011 | Nekaris and Bearder 2011 |

**Trait dataset references**

Andriaholinirina N, Fausser JL, Roos C, Zinner D, Thalmann U, Rabarivola C, Ravoarimanana I, Ganzhorn JU, Meier B, and Hilgartner R. 2006. Molecular phylogeny and taxonomic revision of the sportive lemurs (*Lepilemur*, Primates). BMC Evolutionary Biology 6(1):17.

Atsalis S. 2007. A Natural History of the Brown Mouse Lemur. New Jersey: Pearson Prentice Hall.

Baden AL, Brenneman RA, and Louis EE. 2008. Morphometrics of wild black‐and‐white ruffed lemurs [*Varecia variegata*; Kerr, 1792]. American journal of primatology 70(10):913-926.

Balko EA, and Underwood H. 2005. Effects of forest structure and composition on food availability for *Varecia variegata* at Ranomafana National Park, Madagascar. American Journal of Primatology 66(1):45-70.

Biebouw K. 2009. Home range size and use in *Allocebus trichotis* in Analamazaotra special reserve, central eastern Madagascar. International journal of primatology 30(2):367-386.

Colquhoun IC. 1997. A predictive socioecological study of the black lemur (*Eulemur macaco macaco*) in northwestern Madagascar: Washington University, Department of Anthropology.

Craul M, Zimmermann E, Rasoloharijaona S, Randrianambinina B, and Radespiel U. 2007. Unexpected species diversity of Malagasy primates (*Lepilemur* spp.) in the same biogeographical zone: a morphological and molecular approach with the description of two new species. BMC Evolutionary Biology 7(1):83.

Curtis DJ. 2006. Diurnality in lemurs. In: Gould L, and Sauther ML, editors. Lemurs: Ecology and Adaptation. New York: Springer. p 133-157.

Donati G, Bollen A, Borgognini-Tarli S, and Ganzhorn J. 2007. Feeding over the 24-h cycle: dietary flexibility of diurnal collared lemurs (*Eulemur collaris*). Behavioral Ecology and Sociobiology 61(8):1237-1251.

Donati G, and Borgognini-Tarli SM. 2006. From darkness to daylight: diurnal activity in primates. Journal of Anthropological Sciences 84(1):7-32.

Donati G, Santini L, Razafindramanana J, Boitani L, and Borgognini‐Tarli S. 2013. (Un‐) expected nocturnal activity in “Diurnal” *Lemur catta* supports diurnality as one of the key adaptations of the lemurid radiation. American journal of physical anthropology 150(1):99-106.

Faulkner A, and Lehman S. 2006. Feeding patterns in a small-bodied nocturnal folivore (*Avahi laniger*) and the influence of leaf chemistry: a preliminary study. Folia Primatologica 77(3):218-227.

Fietz J. 2003. Primates: *Cheirogaleus*, dwarf lemurs or fat-tailed lemurs. The Natural History of Madagascar:1307-1309.

Fietz J, and Ganzhorn JU. 1999. Feeding ecology of the hibernating primate *Cheirogaleus medius*: how does it get so fat? Oecologia 121(2):157-164.

Freed B. 1996. Co-occurrence among crowned lemurs (*Lemur coronatus*) and Sanford's lemurs (*Lemur fulvus sanfordi*) of Madagascar. St. Louis, MO: Washington University.

Ganzhorn J, Abraham J, and Razanahoera-Rakotomalala M. 1985. Some aspects of the natural history and food selection of *Avahi laniger*. Primates 26(4):452-463.

Ganzhorn JU. 2002. Distribution of a folivorous lemur in relation to seasonally varying food resources: integrating quantitative and qualitative aspects of food characteristics. Oecologia 131(3):427-435.

Glander K, and Powzyk J. 1998. Morphometrics of wild *Indri indri* and Propithecus diadema diadema. Folia Primatologica 69(Suppl 1):399.

Glander KE, Wright PC, Daniels PS, and Merenlender AM. 1992. Morphometrics and testicle size of rain forest lemur species from southeastern Madagascar. Journal of Human Evolution 22(1):1-17.

Godfrey LR, Semprebon GM, Jungers WL, Sutherland MR, Simons EL, and Solounias N. 2004. Dental use wear in extinct lemurs: evidence of diet and niche differentiation. Journal of Human Evolution 47(3):145-169.

Godfrey LR, Winchester JM, King SJ, Boyer DM, and Jernvall J. 2012. Dental topography indicates ecological contraction of lemur communities. American Journal of Physical Anthropology 148(2):215-227.

Gunnell GF, and Silcox MT. 2010. Primate origins: the early Cenozoic fossil record. A companion to biological anthropology:275-294.

Heesy CP, and Ross CF. 2001. Evolution of activity patterns and chromatic vision in primates: morphometrics, genetics and cladistics. Journal of Human Evolution 40(2):111-149.

Hemingway CA. 1998. Selectivity and variability in the diet of Milne-Edwards' sifakas (*Propithecus diadema edwardsi*): implications for folivory and seed-eating. International Journal of Primatology 19(2):355-377.

Irwin MT. 2006. Ecological impacts of forest fragmentation on diademed sifakas (*Propithecus diadema*) at Tsinjoarivo, eastern Madagascar: implications for conservation in fragmented landscapes. PhD Dissertation, Stony Brook University, Stony Brook, NY.

Irwin MT. 2007. Ecologically enigmatic lemurs: the sifakas of the eastern forests (*Propithecus candidus, P. diadema, P. edwardsi, P. perrieri*, and *P. tattersalli*). In: Gould L, and Sauther ML, editors. Lemurs: Ecology and Adaptation. New York: Springer. p 305-326.

Johnson S, Gordon A, Stumpf R, Overdorff D, and Wright P. 2005. Morphological Variation in Populations of *Eulemur albocollaris* and *E. fulvus rufus*. International Journal of Primatology 26(6):1399-1416.

Johnson, S. E. 2007. Evolutionary divergence in the brown lemur species complex. Pp. 187-210. Lemurs. Springer.

Junge RE, Dutton CJ, Knightly F, Williams CV, Rasambainarivo FT, and Louis EE. 2009. Comparison of Biomedical Evaluation for White-Fronted Brown Lemurs (*Eulemur fulvus albifrons*) from Four Sites in Madagascar. Journal of Zoo and Wildlife Medicine 39(4):567-575.

Junge RE, and Louis Jr EE. 2005. Biomedical evaluation of two sympatric lemur species (*Propithecus verreauxi deckeni* and *Eulemur fulvus rufus*) in Tsiombokibo Classified Forest, Madagascar. Journal of Zoo and Wildlife Medicine 36(4):581-589.

Jungers WL, Demes B, and Godfrey LR. 2008. How big were the “giant” extinct lemurs of Madagascar? In: Fleagle JG, and Gilbert CC, editors. Elwyn Simons: A search for origins. New York: Springer. p 343-360.

Kappeler P, Rasoloarison R, Razafimanantsoa L, Walter L, and Roos C. 2005. Morphology, behaviour and molecular evolution of giant mouse lemurs (*Mirza* spp.) Gray, 1870, with description of a new species. Primate Reports 71:3-26.

Kappeler PM. 1991. Patterns of sexual dimorphism in body weight among prosimian primates. Folia Primatologica 57(3):132-146.

Kawamichi T, and Kawamichi M. 1979. Spatial organization and territory of tree shrews. Animal Behaviour 27(2):381-393.

Kay RF, and Kirk EC. 2000. Osteological evidence for the evolution of activity pattern and visual acuity in primates. American Journal of Physical Anthropology 113(2):235-262.

Koyama N, Aimi M, Kawamoto Y, Hirai H, Go Y, Ichino S, and Takahata Y. 2008. Body mass of wild ring-tailed lemurs in Berenty Reserve, Madagascar, with reference to tick infestation: a preliminary analysis. Primates 49(1):9-15.

Lewis RJ, and Kappeler P. 2005. Seasonality, body condition, and timing of reproduction in *Propithecus verreauxi verreauxi* in the Kirindy Forest. American Journal of Primatology 67(3):347-364.

Louis EE. 2006. Molecular and morphological analyses of the sportive lemurs (Family Megaladapidae: Genus *Lepilemur*) reveals 11 previously unrecognized species: Museum of Texas Tech University.

Louis EE, Coles MS, Andriantompohavana R, Sommer JA, Engberg SE, Zaonarivelo JR, Mayor MI, and Brenneman RA. 2006. Revision of the mouse lemurs (Microcebus) of eastern Madagascar. International Journal of Primatology 27(2):347-389.

Louis Jr EE, Engberg SE, McGuire SM, McCormick MJ, Randriamampionona R, Ranaivoarisoa JF, Bailey CA, Mittermeier RA, and Lei R. 2008. Revision of the mouse lemurs, *Microcebus* (Primates, Lemuriformes), of northern and northwestern Madagascar with descriptions of two new species at Montagne d'Ambre National Park and Antafondro Classified Forest. Primate Conservation 23:19-38.

Meyers DM, and Wright PC. 1993. Resource tracking: food availability and *Propithecus* seasonal reproduction. In: Sterling E, Kappeler P, and Ganzhorn JU, editors. Lemur social systems and their ecological basis. New York: Springer. p 179-192.

Mittermeier RA, Louis EE, Richardson M, Schwitzer C, Langrand O, Rylands AB, Hawkins F, Rajaobelina S, Ratsimbazafy J, Rasoloarison R et al. . 2010. Lemurs of Madagascar, 3rd Ed. Washinton D.C.: Conservation International. 762 p.

Mutschler T. 2002. Alaotran gentle lemur: some aspects of its behavioral ecology. Evolutionary Anthropology: Issues, News, and Reviews 11(S1):101-104.

Mutschler T, Feistner AT, and Nievergelt CM. 1998. Preliminary field data on group size, diet and activity in the Alaotran gentle lemur *Hapalemur griseus alaotrensis*. Folia Primatologica 69(5):325-330.

Nekaris KA-I, Pambudi JAA, Susanto D, Ahmad RD, and Nijman V. 2014. Densities, distribution and detectability of a small nocturnal primate (Javan slow loris *Nycticebus javanicus*) in a montane rainforest. Endangered Species Research 24:95-103.

Nekaris KAI, and Bearder SK. 2011. The Lorisiform Primates of Asia and Mainland Africa: Diversity Shrouded in Darkness. In: Campbell CJ, Fuentes A, MacKinnon KC, Bearder SK, and Stumpf R, editors. Primates in Perspective. New York: Oxford University Press.

Norscia I, Ramanamanjato JB, and Ganzhorn JU. 2012. Feeding patterns and dietary profile of nocturnal southern woolly lemurs (*Avahi meridionalis*) in southeast Madagascar. International Journal of Primatology 33(1):150-167.

Nowak RM. 1999. Walker's Mammals of the World, 6th edition. Baltimore: JHU Press.

Olivieri G, Zimmermann E, Randrianambinina B, Rasoloharijaona S, Rakotondravony D, Guschanski K, and Radespiel U. 2007. The ever-increasing diversity in mouse lemurs: three new species in north and northwestern Madagascar. Molecular phylogenetics and evolution 43(1):309-327.

Radespiel U, Ratsimbazafy JH, Rasoloharijaona S, Raveloson H, Andriaholinirina N, Rakotondravony R, Randrianarison RM, and Randrianambinina B. 2012. First indications of a highland specialist among mouse lemurs (*Microcebus* spp.) and evidence for a new mouse lemur species from eastern Madagascar. Primates 53(2):157-170.

Randrianambinina B, Rakotondravony D, Radespiel U, and Zimmermann E. 2003. Seasonal changes in general activity, body mass and reproduction of two small nocturnal primates: a comparison of the golden brown mouse lemur (Microcebus ravelobensis) in Northwestern Madagascar and the brown mouse lemur (*Microcebus rufus*) in Eastern Madagascar. Primates 44(4):321-331.

Rasmussen MA. 1999. Ecological influences on activity cycle in two diurnal primates, the mongoose lemur (*Eulemur mongoz*) and the common brown lemur (*Eulemur fulvus fulvus*): Duke University.

Rasoloarison RM, Weisrock DW, Yoder AD, Rakotondravony D, and Kappeler PM. 2013. Two new species of mouse lemurs (Cheirogaleidae: *Microcebus*) from eastern Madagascar. International Journal of Primatology 34(3):455-469.

Schmid J, and Kappeler PM. 1998. Fluctuating sexual dimorphism and differential hibernation by sex in a primate, the gray mouse lemur ( *Microcebus murinus* ). Behavioral Ecology and Sociobiology 43(2):125-132.

Schwitzer N, Kaumanns W, Seitz PC, and Schwitzer C. 2007. Diurnal activity patterns of the blue-eyed black lemur Eulemur macaco flavifrons in intact and degraded forest fragments. Endangered Species Research 3(2):239-247.

Smith RJ, and Jungers WL. 1997. Body mass in comparative primatology. Journal of Human evolution 32(6):523-559.

Sterling EJ. 1993. Behavioral ecology of the aye-aye (*Daubentonia madagascariensis*) on Nosy Mangabe, Madagascar: Yale University.

Terranova CJ, and Coffman BS. 1997. Body weights of wild and captive lemurs. Zoo Biology 16(1):17-30.

Thalmann U. 2001. Food resource characteristics in two nocturnal lemurs with different social behavior: Avahi occidentalis and Lepilemur edwardsi. International Journal of Primatology 22(2):287-324.

Thalmann U, and Geissmann T. 2005. New species of woolly lemur *Avahi* (Primates: Lemuriformes) in Bemaraha (central western Madagascar). American Journal of Primatology 67(3):371-376.

Vasey N. 2000. Niche separation in *Varecia variegata rubra* and *Eulemur fulvus albifrons*: I. Interspecific patterns. American Journal of Physical Anthropology 112(3):411-431.

Vasey N. 2005. Activity budgets and activity rhythms in red ruffed lemurs (*Varecia rubra*) on the Masoala Peninsula, Madagascar: seasonality and reproductive energetics. American Journal of Primatology 66(1):23-44.

Yoder AD, Rasoloarison RM, Goodman SM, Irwin JA, Atsalis S, Ravosa MJ, and Ganzhorn JU. 2000. Remarkable species diversity in Malagasy mouse lemurs (Primates, *Microcebus*). Proceedings of the National Academy of Sciences 97(21):11325-11330.

Zaramody A, Fausser J-L, Roos C, Zinner D, Andriaholinirina N, Rabarivola C, Norscia I, Tattersall I, and Rumpler Y. 2006. Molecular phylogeny and taxonomic revision of the eastern woolly lemurs (*Avahi laniger*). Primate Reports 74:9-20

**Lineage and phenotypic diversification rates:** Many alternative models of diversification and techniques for estimating those model parameters from phylogenetic trees exist[18]. To measure lineage diversification rates through time, I quantified the diversification dynamics of strepsirrhines using four methods, two Bayesian inference approaches (Bayesian Analysis of Macroevolutionary Mixtures, BAMM v2.5[4], TESS [19]), and two maximum likelihood approaches (Modeling Evolutionary Diversification Using Stepwise Akaike Information Criterion, MEDUSA[7], and Phylogenetic Analysis of Diversification, RPANDA[6]). The BAMM analysis uses reversible-jump Markov Chain Monte Carlo (rjMCMC) approach to sample models of lineage and phenotypic diversification with heterogeneous rates through time and among lineages. BAMM assumes a single time-varying diversification scenario to start and samples models in which one or more shifts in diversification rates occur across the tree in proportion to the model’s probability. Speciation rates can vary through time in each diversification regime, while extinction is assumed to be constant[4]. The speciation rates can also be constrained to be constant through time but heterogeneous across the tree, allowing a direct comparison of the time-varying and time-constant models. Similarly to BAMM, TESS uses rjMCMC to sample alternate diversification histories, given prior parameters on the speciation and extinction rates as well as the hypothesized timing of mass extinction events (here set to 34 million years ago to reflect the Eocene-Oligocene boundary). MEDUSA uses a step-wise information criterion approach to test for multiple rate regimes on the tree. First a single rate model is fit to the whole tree, and then the tree is broken into smaller subtrees iteratively, with new models fit to each subtree. The single-rate model is compared to more complex models using the second-order Akaike Information Criterion (AICc) and the algorithm stops searching for more complex models when the there is no improvement in AICc. A backward procedure then removes rate shifts from the most complex model to test if model fit is reduced. Because MEDUSA has been shown to have high false positive rates [20], I first simulated 1000 trees under a constant birth-death process with speciation rate = 0.15 and extinction = 0.05 (based on BAMM results), the max number of tips = 120 based on the empirical phylogeny, and the tree depth = 50 using the *rphylo* function in the R package ape [21] and ran MEDUSA over the 1000 trees to find the delta AICc value that correctly rejects models with multiple diversification regimes. This delta AICc value was used in the analyses of the empirical trees. In contrast to the automatic-detection approaches described thus far, RPANDA implements the maximum likelihood equations derived by Morlon and colleagues[22, 23], allowing the user to compare models of pure birth (speciation only) in which speciation rates are constant or change linearly or exponentially through time, and birth-death models with time-constant and time-varying speciation and/or extinction rates. Further, an environmental-dependent model estimates speciation or extinction rates varying through time in relation to environmental variables[22]. RPANDA does not, however, detect shifts in diversification across the tree automatically; instead, the user can compare models for the full tree as well as subtrees of interest for which rate shifts are hypothesized *a priori*. The diversification methods used here, like most phylogeny-based speciation and extinction models, assume all observed tips survive to the present and therefore can only handle ultrametric trees[24]. To retain the subfossil lemurs, which are so important to test predictions about lineage and phenotypic evolutionary rates, I added the time since extinction to their branch lengths to bring those tips to the present. This is not expected to bias the analyses because the amount of evolutionary change expected within the last 2000 years since extinction is negligible compared to ~30-50 million years of evolutionary history.

BAMM analyses

To account for non-uniform incomplete species sampling, I specified the proportion of sampled species per genus (Table S1). To set prior parameter probabilities for the BAMM analyses, for speciation and extinction rate analyses I used a rate shift Poisson parameter prior of 1, which places the highest probability on fewer rate shifts and is appropriate for small trees[4]. Other priors were adjusted empirically based on the trees using the *BAMMtools* package v2.0.6[25] in the R statistical environment[26]. For the speciation and extinction rate analyses, these were lambda (speciation rate) = 2.7 – 2.8, lambda shift prior (probability of a shift in speciation rates) = 0.02, extinction prior = 2.7 – 2.8. For the phenotypic analysis of the body mass dataset, I used default priors with the trait range prior based on a uniform distribution from the range of the observed data, Poisson rate prior = 1.0, and beta shift prior (probability of a shift in trait rate) = 0.05. The prior values in the lineage and phenotypic analyses place the highest probability on fewer rate shifts; in fact, the null model is no shift. I also ran a separate analysis with the Poisson rate prior on the number of diversification rate shifts = 10, which places equal low probability on 0 – 10 rate shifts. The advantage of the BAMM analysis is that, because samples of rate shift models are taken using a rjMCMC algorithm, the probability of the model (marginal likelihood) with one or more rate shifts can be compared to the probability of the model with no rate shifts using Bayes factors (marginal likelihood of a model with a shift / likelihood of a model with no shift). This technique enables comparisons of the probability and location of rate shifts in the tree based on the prior probability of a shift, which is determined by branch lengths. Further, the branch-specific posterior probability of a shift can be compared to the branch-specific prior probability because the prior is based on the branch length. High species richness in a clade with relatively short branches would be inferred to have a greater probability of a rate shift than expected under the prior. Bayes factors > 3 suggested support for the alternative model[27]. To constrain the BAMM model to be time-invariant, I specified the *updateRateLambdaShift* and *lambdaIsTimeVariablePrior* priors to 0.

I ran the BAMM analysis for 100 million generations, recording parameters every 5000 generations and determined convergence by plotting the model likelihoods by generations and calculating the effective sample size values in the *coda* package[28] for R. Effective sample size (ESS) values were > 200 for all parameters. Histograms of the posterior distribution of diversification rate estimates at the lemur and loris nodes were compared to determine if diversification rates differed between the two lineages. Branch-specific shift probabilities were evaluated using Bayes factors of the posterior probability of rate shifts on each branch compared to the prior probability of a shift expected given the branch lengths. I obtained and plotted estimates of lineage and phenotypic diversification rates through time using the *getRateThroughTimeMatrix* function in *BAMMtools*.

To compare the results from BAMM across alternative tree topologies (the MCC tree and the alternate position of *Megaladapis*), I ran the BAMM analysis as described above for the alternate tree and extracted the mean net diversification rates at the tips from both analyses (*getTipRates* function in *BAMMtools*). I then conducted regressions of these tip rates to determine if they were strongly correlated, or if the rates were different between the two trees.

BAMM can also be used to model the rate(s) of trait evolution, assuming a single Brownian Motion model to start and allowing the rate parameter of the Brownian Motion model to change through time and among lineages, sampling based on the posterior probabilities using the same rjMCMC procedure. To supplement the results from BAMM on the evolution of body mass, I also used maximum likelihood algorithms to test which of the following models of trait evolution best fit the body mass dataset: Brownian Motion, delta, drift, early burst, kappa, lambda, Ornstein-Uhlenbeck, trend, and white noise using the *fitContinuous* function in the R package geiger [29]. Each of these models is associated with different macroevolutionary processes, with the goal of determining via AICc comparison which model best fits the data [30, 31]. I iterated the *fitContinuous* function over 100 trees from the posterior distribution of trees for all strepsirrhines and for lemurs only and present the means and standard deviations of AICc and delta AICc scores for each model.

MEDUSA analysis

For the MEDUSA analysis, I accounted for nonrandom incomplete sampling by supplying the missing species richness per genus. The delta AICc threshold for retaining a more complex model over a simpler model was 5.19 by default, based on the number of tips in the tree. I increased the AICc threshold to > 7 based on the results of simulations presented in the SM Results section. I ran the MEDUSA algorithm with the following settings: specifying to use the second-order AIC (AICc, for small sample sizes), setting the maximum number of partitions (rate regimes) to 5 to minimize the number of models examined, the models allowed to be mixed (both pure birth and birth-death), the cut point allowed to be either at the stem or node, stepBack procedure set to true to allow backwards model removal, and the initial diversification rate (r) = 0.07 and the initial extinction fraction (extinction/speciation) set to 0.4 (based on the BAMM results).

RPANDA analyses

RPANDA implements the maximum likelihood diversification models described by Morlon and colleagues[22, 23]. I specifically fit the following seven models to the whole strepsirrhine tree, the lemur tree and the lorisiform tree: 1) pure birth constant speciation, 2) pure birth exponential speciation, 3) pure birth linear speciation, 4) constant rate birth-death, 5) birth-death with exponential speciation and constant extinction, 6) birth-death with constant speciation and exponential extinction, and 7) birth-death with exponential speciation and extinction. For lemurs, I tested an additional two models to determine if the patterns for lemurs can be explained by abiotic and biotic interactions. These models fit a time-varying speciation rate with a dependence on an independent variable and constant extinction rates[22]. I fit one model in which the independent variable was the mean global temperature throughout the Cenozoic era estimated from the proportions of oxygen isotopes[32]. In the second model, the independent variable was the past diversity of carnivores. Past carnivore diversity was estimated from the lineage-through-time plot of the eight-species Malagasy carnivore time tree extracted from a complete carnivore time-calibrated phylogeny[33] using the R package *ape*[21]. Initial parameter values were set based on the results of the BAMM analyses (b=0.15, e=0.05) and runs with higher and lower initial parameters returned almost identical values. In all analyses, the sampling fraction was specified (strepsirrhine tree: 116/145 species, lemur tree: 101/117, lorisiform tree: 19/28).

I compared model fit using the second-order Akaike Information Criterion (AICc) and the AICc weights to determine the relative probability of each model given the candidate set of models. Speciation and extinction rates were averaged across the set of candidate models, weighted by their AICc weights. I used posterior predictive modelling to assess model adequacy. For the RPANDA analyses of 100 trees randomly selected from the posterior distribution of the total evidence analysis, I recorded the delta AICc values comparing the constant-rate and exponential time-varying pure-birth model, and the constant-rate and exponential time-varying birth-death model, as well as the model-averaged speciation and time-varying parameters.

**Posterior predictive model checking of lineage diversification dynamics:**

The results of the lineage diversification analyses suggested that it was difficult to distinguish a constant-rate birth-death process from a time-varying pure-birth process. In addition, while BAMM and MEDUSA analyses suggest no support for a diversification rate shift between lemurs and lorisiforms, the results from RPANDA suggested that the diversification dynamics of lemurs may in fact be different from those of lorisiforms. To further investigate if the patterns predicted from the lineage diversification model parameters fit the data at hand, I used a posterior predictive checks framework[34]. In this posterior predictive model check, the estimated speciation, extinction, and time-varying parameters on the observed phylogeny were used to simulate phylogenies. Summary statistics of tree characteristics were then compared between the simulated phylogenies and the observed phylogeny to determine if the simulations recover similar tree characteristics to those observed empirically. I chose the following summary statistics for comparison of the simulated and observed trees: the number of tips (natural log transformed), the gamma statistic, which captures the tree shape as a function of height above the root [35] (using *gammaStat* function in the Geiger package for R[36]), the beta statistic, which captures the balance of the branching structure[37] (using the *maxlik.betasplit* function in the apTreeshape package for R[38]), and the skewness and kurtosis of the phylogenetic branching times (*skewness* and *kurtosis* functions in the R package *moments*[39] and the *branching.times* function in the *ape* package[21]).

*Time constant one- and two-rate simulations*

One rate: I simulated 1000 trees under a time constant model with a single birth-death rate taken from the results of the RPANDA birth-death analysis on the full strepsirrhine tree (b=0.16, d=0.08, time=53Ma, the length of the total strepsirrhine tree). These parameter estimates were similar to those estimated by BAMM. Two rate: I simulated 1000 trees under a time constant model with two rates – one birth-death rate with parameters from the lemur tree (b=0.15, d=0.07; parameters were a compromise between the results of RPANDA and BAMM, time=48Ma), and one pure-birth rate with parameters from the lorisiform tree (b=0.08, d=0 from RPANDA results, time=34Ma). These trees were simulated separately, and a root edge was added to the trees (lemurs=5.34Ma, lorisiforms=19.39Ma, from the empirical tree) before binding them into a single two-rate tree.

*Time-varying rate simulations*

I simulated 1000 trees under a time-varying speciation rate where speciation was an exponential function of time, according to the following function: 0.15/(1 + exp(-0.012*t)), where t=time, 0.15 is the estimated birth rate parameter at the present time, and -0.012 is the time-varying parameter, based on the RPANDA results. This set of parameter values resulted in trees with increasing speciation rates through time based on the results of the time-varying BAMM analysis and the results of RPANDA time-variable models.

**Adaptive phenotypic evolution:**

To test the adaptive relationship between phenotype and niche divergence, I used two approaches: a phylogenetic generalized least squares (PGLS) approach and a generalized Ornstein-Uhlenbeck (OU) model approach. First, I tested the relationship between body mass (ln transformed) and the combined diet and activity niches (treated as factors), specifying the phylogenetic distances as the correlation structure for the residuals and modifying the correlation structure according to the following evolutionary models and associated parameters: Brownian motion (sigma squared), Pagel’s lambda (lambda), OU (theta, sigma squared and alpha), and Blomberg’s g (accelerating/decelerating evolution). I ran a separate generalized linear model for each evolutionary model using the *gls* function in the R package *nlme*[40] and the correlation matrices for the evolutionary models were calculated using functions from the *ape* package. I compared the fit of each PGLS model against a null model with only the intercept to test if the model explained significant variation in the data using analysis of variance. I then compared the fit of the models to the data using AICc scores, and used model averaging to compute the coefficients and standard errors, given the candidate set of models and their AICc weights. I assessed the model adequacy using the posterior predictive checks approach implemented in the *arbutus*[41] R package. In this package, I simulated traits on trees rescaled to the fitted evolutionary model parameters for each model (BM, Pagel’s lambda, OU, Blomberg’s g) and then compared the observed values of six test statistics based on phylogenetic independent contrasts of observed traits to the contrasts of the simulated data. The test statistics were: the mean of the squared contrasts (M_sig_) which indicates if rates are over- or underestimated; the coefficient of variation of absolute contrasts (C_var_) which assesses rate heterogeneity across the tree; the slope of the absolute contrasts against expected variance (S_var_), where a negative slope indicates more evolution per unit time on short than long branches, and a positive relationship may reflect more evolution on long branches than expected; the slope of the linear model fitted to the absolute contrasts against the weighted average value for each node based on evolutionary distance (S_ASR_); the slope of linear model of the absolute contrasts and the node height (node-height test, S_HGT_) which is a test to detect early bursts of trait evolution akin to adaptive radiation theory where positive relationships indicate that greater evolutionary changes occurred at younger nodes than older nodes, and a negative relationship represents greater change in older nodes than younger nodes; the D statistic of the Kolmolgorov-Smirnov test (D_CDF_) to test if the distribution of the contrasts deviates from a normal distribution of mean 0 and standard deviation equal to the root of the mean of squared contrasts, which represents departures of contrasts from normality as seen in jump-diffusion evolution where bursts of rapid evolution occurred heterogeneously across the tree. Significant deviations of the observed from the simulated values indicates that the data do not fit the model adequately.

I then tested the hypothesis that adaptive zones have different phenotypic optima and evolutionary rates (generalized OU models[42]). I first compared the fit of the discrete trait evolution inferences under equal, symmetrical, and asymmetrical rates Mk models using the *fitDiscrete* function in g*eiger*[36] for R and the best model was the equal-rates model, based on the lowest AICc score. I then inferred the ancestral states of combined diet and activity adaptive zones on the nodes of the phylogeny using a maximum-likelihood method and the equal-rates model, which implements the subtree-pruning algorithm to estimate the marginal likelihood of ancestral states (*rerootingMethod* function in the *phytools* package[43] in R). Ancestral states could be inferred with strong support, and the most likely state at each node was extracted and mapped onto the tree for subsequent analysis. I then tested the fit of the following models of body mass evolution in relation to the evolution of adaptive zones: 1) one Brownian Motion model (BM) fits for the whole tree, 2) one adaptive peak model (OU) fits for the whole tree, 3) one unique BM model for each niche regime, each with its own variance, 4) one unique OU for each regime, each with its own optimal mean but the same alpha and variance, 5) same as 4 but with unique variance (σ^2^) parameters for each regime, 6) same as 4 but with unique alpha parameters for each regime, 7) unique OU models for each regime, with alpha, variance and theta estimated for each regime (implemented in the *OUwie* package[42] in R). I did not rescale the tree for this analysis, such that units are in the original time scale, and the root state was estimated from the model, which in the *OUwie* implementation is assumed to be the stationary distribution of the model. Models were compared using AICc and AICc weights. I averaged the model parameters and associated standard errors based on the AICc weights[44]. Finally, to assess the potential effects of uncertainty in the phylogeny and ancestral state estimates, I used Bayesian stochastic character mapping [45] in *phytools* to simulate the evolution of the adaptive zones along each of the 100 trees from the posterior distribution 100 times and fit the models on each of 100 randomly selected trees with the stochastic character maps. To summarize these results, I present the change in AICc from the best model for each model across the 100 *OUwie* runs.

To characterize the multidimensional ‘ecospace’ occupied by lemurs and their sister clade, I transformed the niche dataset on body mass (ln transformed), diet, and activity pattern into a dissimilarity matrix based on the Gower coefficient using the *daisy* function in the R package *cluster* [46]. I controlled for the phylogenetic autocorrelation of trait dissimilarity by taking the residuals of the mantel regression of functional on phylogenetic distances using the *multi.mantel* function in *phytools*. I then projected the residual trait dissimilarity matrix into two-dimensions using nonmetric multidimensional scaling (*metaMDS* function in the R package *vegan* [47]). The two MDS axes together explained ~91% of the variation in the functional distance matrix (stress=0.094).

**Results**

***BAMM results under alternative phylogenetic hypotheses and time-varying compared to time-constant models:***

**Lineage diversification:**

The posterior probability of zero diversification rate shifts was higher than the probability of one, two or three rate shifts for the MCC tree under the time-varying and time-constant speciation models and for each alternate phylogeny, including with alternative placements of the extinct genus *Megaladapis*, only extant lemurs, a phylogeny based on a conservative (~50 taxon) taxonomy, and a phylogeny inferred based on mitochondrial genomes of 23 strepsirrhines including five extinct lemurs (Table S3). These results indicate no shift in diversification dynamics between lemurs and lorisiforms, contrary to the expectation if lemurs evolved via adaptive radiation. When examining the posterior probability of branch-specific rate shifts given the branch-specific prior probability of a rate shift given the branch length (higher prior probability of a shift on longer branches than short branches), there is evidence of a rate shift on the lineages of the lemur genus *Microcebus* (Fig. S1). *Microcebus* seems to have undergone a shift to higher diversification rates than the background rate.

**Fig. S1.** Phylogenetic tree rescaled according to the Bayes factor of a branch-specific shift in diversification. The branch-specific posterior probability of a rate shift is directly compared to the branch-specific prior probability of a rate shift given the rate of speciation and the length of the branch, and the results suggest that lineages in the genus *Microcebus* had branch-specific Bayes factors > 6, indicating strong support for rate shifts on the branches in this genus. Short grey branches have Bayes factors < 3, while long black branches have Bayes factors > 3.


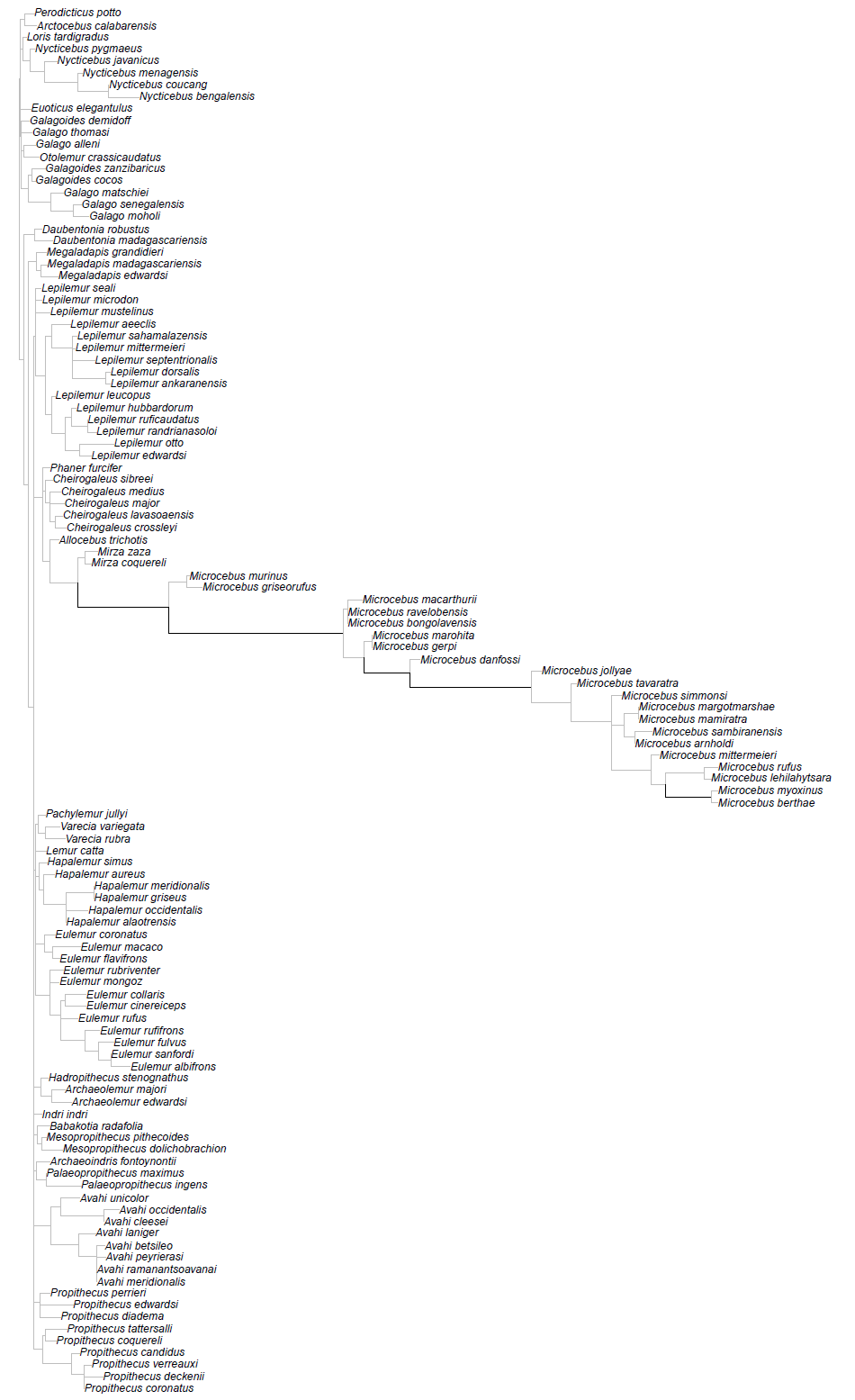


The macroevolutionary models implemented in BAMM include time-varying and time-constant speciation rates. With the MCC phylogeny, the posterior probabilities of the time-varying and time-constant models were nearly identical, suggesting they cannot be distinguished with this phylogeny (Fig. S2). Thus a constant-rate speciation model fits the phylogeny as well as a time-varying model.

**Fig. S2.** Density plot of posterior distribution of likelihoods from time-varying and time-constant BAMM runs. The distributions are almost identical, indicating the two models have nearly identical probabilities.


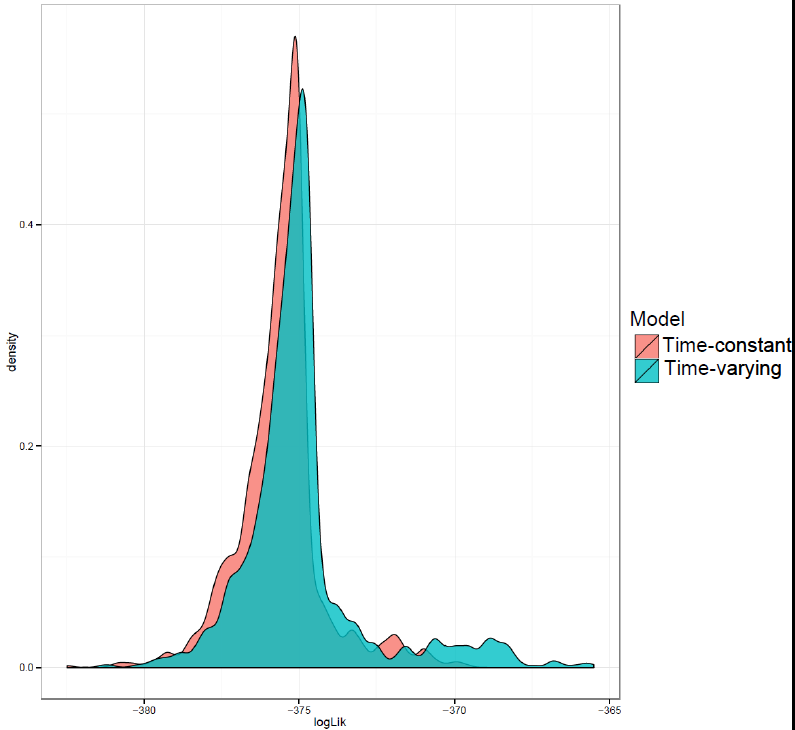


**Table S3.** Full results for model comparisons of diversification dynamics, using the R package RPANDA. b = birth rate (per-lineage rate of speciation, # of lineages My^-1^), d= death rate (per-lineage rate of extinction, # of lineages My^-1^), time-varying parameter = parameter determining the rate of change of speciation and/or extinction as a function of time, net div = net diversification (speciation - extinction), logLik = log likelihood, AICc = second-order Akaike Information Criterion, ΔAICc = change in AICc from the best model, AICc w = AICc weight, or the relative likelihood of eah model given the candidate set of models. These AICc weights and model parameters were used to produce the model-averaged parameters in Table 1 of the main text. Results are presented for A) the whole strepsirrhine tree, B) the lemur subtree, and C) the lorisiform subtree.

1. Whole strepsirrhine tree

| **Model** | **b** | **d** | **Time-varying**  **parameter** | **Net Div** | **logLik** | **AICc** | **ΔAICc** | **AICc w** |
| --- | --- | --- | --- | --- | --- | --- | --- | --- |
| Pure birth constant speciation | 0.108 | 0.000 | - | 0.108 | -380.690 | 763.424 | 4.118 | 0.035 |
| Pure birth exponential speciation | 0.137 | 0.000 | -0.023 |  | -377.600 | 759.307 | 0.000 | 0.274 |
| Pure birth linear speciation | 0.131 | 0.000 | -0.002 |  | -377.793 | 759.692 | 0.385 | 0.226 |
| Constant birth-death | 0.157 | 0.085 | - | 0.072 | -377.795 | 759.697 | 0.390 | 0.225 |
| Birth-death with exponential speciation & constant extinction | 0.146 | 0.038 | -0.012 | 0.109 | -377.533 | 761.280 | 1.974 | 0.102 |
| Birth-death with exponential extinction & constant speciation | 0.148 | 0.057 | 0.017 | 0.091 | -377.529 | 761.272 | 1.966 | 0.103 |
| Birth-death with exponential extinction & exponential speciation | 0.149 | 0.063 | 0.004, 0.021 | 0.085 | -377.529 | 763.417 | 4.111 | 0.035 |

1. Lemur subtree

| **Model** | **b** | **d** | **Time-varying**  **parameter** | **Net Div** | **logLik** | **AICc** | **ΔAICc** | **AICc w** |
| --- | --- | --- | --- | --- | --- | --- | --- | --- |
| Pure birth constant speciation | 0.118 | 0.000 | - | 0.118 | -310.188 | 622.418 | 3.941 | 0.037 |
| Pure birth exponential speciation | 0.152 | 0.000 | -0.028 |  | -307.175 | 618.477 | 0.000 | 0.267 |
| Pure birth linear speciation | 0.145 | 0.000 | -0.003 |  | -307.232 | 618.590 | 0.114 | 0.253 |
| Constant birth-death | 0.170 | 0.093 | - | 0.078 | -307.716 | 619.559 | 1.082 | 0.156 |
| Birth-death with exponential speciation & constant extinction | 0.152 | -0.002 | -0.027 | 0.154 | -307.177 | 620.609 | 2.132 | 0.092 |
| Birth-death with exponential extinction & constant speciation | 0.156 | 0.049 | 0.030 | 0.108 | -307.203 | 620.661 | 2.184 | 0.090 |
| Birth-death with exponential extinction & exponential speciation | 0.152 | 0.000 | -0.028, 0.003 | 0.152 | -307.176 | 622.783 | 4.306 | 0.031 |
| Birth-death with exponential speciation related to temperature, constant extinction | 0.165 | 0.127 | -0.077 | 0.038 | -378.143 | 762.501 | 144.024 | 0.000 |
| Birth-death with exponential speciation related to past carnivore diversity, constant extinction | 0.079 | 0.107 | 0.093 | -0.028 | -307.406 | 621.068 | 2.591 | 0.073 |

1. Lorisiform subtree

| **Model** | **b** | **d** | **Time-varying**  **parameter** | **Net Div** | **logLik** | **AICc** | **ΔAICc** | **AICc w** |
| --- | --- | --- | --- | --- | --- | --- | --- | --- |
| Pure birth constant speciation | 0.078 | 0.000 | - | 0.078 | -60.444 | 123.137 | 0.000 | 0.498 |
| Pure birth exponential speciation | 0.081 | 0.000 | -0.003 |  | -60.439 | 125.678 | 2.540 | 0.140 |
| Pure birth linear speciation | 0.081 | 0.000 | 0.000 |  | -60.439 | 125.678 | 2.541 | 0.140 |
| Constant birth-death | 0.081 | 0.005 | - | 0.076 | -60.441 | 125.682 | 2.545 | 0.139 |
| Birth-death with exponential speciation & constant extinction | 0.081 | E-6 | -0.003 | 0.081 | -60.439 | 128.592 | 5.455 | 0.033 |
| Birth-death with exponential extinction & constant speciation | 0.086 | 0.000 | 0.715 | 0.086 | -60.140 | 127.995 | 4.857 | 0.044 |
| Birth-death with exponential extinction & exponential speciation | 0.069 | 0.001 | 0.018, 0.181 | 0.068 | -60.383 | 131.844 | 8.707 | 0.006 |

**Fig. S3.** Comparison of tree characteristics among trees simulated under different models of evolution. Dashed horizontal lines in each plot represent the value observed from the strepsirrhine MCC tree. The boxes enclose the first through third quartiles, the black bar represents the median, and whiskers extend to 1.5 times the interquartile range. Points are beyond 1.5 times the interquartile range. A) lntips = natural log of number of tips, B) skewness = the statistical skew of branch lengths, C) gamma = the gamma statistic which measures the temporal distribution of nodes in the tree, compared to a constant-rate prediction, D) kurtosis = the statistical kurtosis of branch lengths, E) beta = the beta statistic which measures the amount of imbalance in the tree compared to a constant-rate prediction.


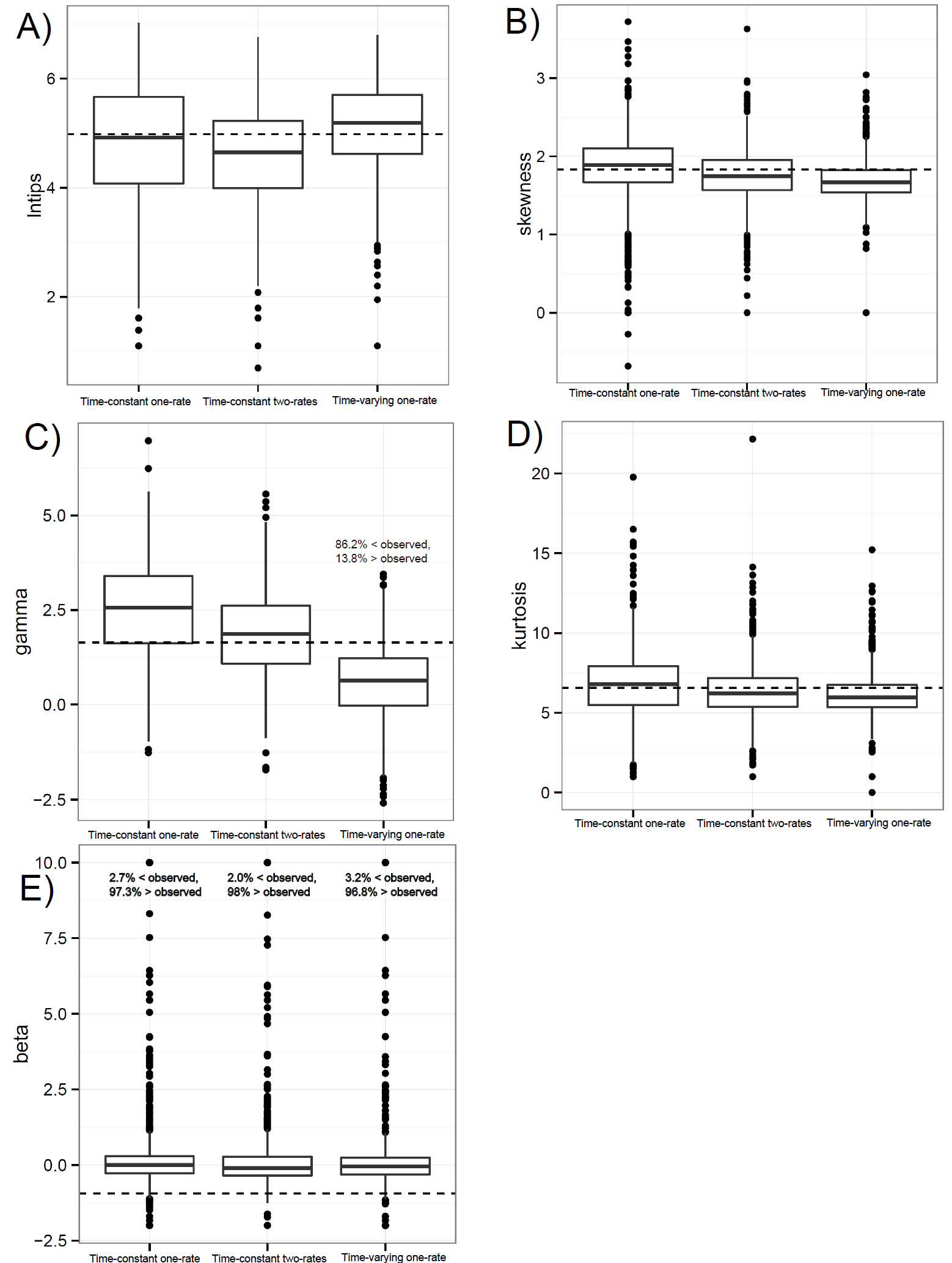


**Table S4.** The posterior probabilities of zero, one, two, or three rate shifts on four alternate strepsirrhine phylogenies, and for the MCC tree, under time-varying and time-constant speciation rate models. For all topologies, the posterior probability of zero shifts is higher than for > 0 shifts.

| # shifts | MCC tree, time-varying, prior = 1 | MCC tree, time-varying, prior = 10 | MCC tree, time-constant | Alternate *Megaladapis* tree, time-varying | Extant-only tree, time-varying | Conservative taxonomy tree, time-varying | Kistler tree |
| --- | --- | --- | --- | --- | --- | --- | --- |
| 0 | 0.76 | 0.56 | 0.56 | 0.55 | 0.53 | 0.66 | 0.63 |
| 1 | 0.18 | 0.25 | 0.30 | 0.32 | 0.33 | 0.27 | 0.25 |
| 2 | 0.045 | 0.10 | 0.11 | 0.11 | 0.11 | 0.07 | 0.08 |
| 3 | 0.011 | 0.05 | 0.02 | 0.02 | 0.03 | 0.009 | 0.03 |

**Comparison of tip rates of net diversification inferred using the MCC tree and the tree with alternate position of *Megaladapis***

The mean and median net diversification rates at the tips of the phylogenies were extracted from the BAMM analysis of the MCC tree and the MCC tree with the alternate position of *Megaladapis* as sister to Lemuridae[8][5] using the *getTipRates* function in *BAMMtools*. The means and medians did not differ markedly, and means were used in subsequent analyses. I compared the mean tip rates between the analyses on the two trees using summary statistics and by conducting linear regressions on the raw tip rate data as well as the phylogenetic independent contrasts (PICs) of the tip rates, calculated using the *ape* package in R[21]. Regressions of PICs were calculated with the y-intercept set to 0, following[48]. PICs were calculated separately for the rates inferred from each tree. The regression of raw tip rates and PICs suggest the rates are strongly correlated between the two topologies (lm of raw data: r=0.86, p<0.001, R^2^=0.63; lm of PICs: r=0.46, p<0.001, R^2^=0.15). Thus, the inferences on diversification dynamics drawn from the MCC tree hold across the two tree topologies. Further, the speciation and extinction rates inferred across the four topologies used here were similar (Table S5), except that the extinction rates were slightly higher in the extant-only analysis, possibly reflecting the ability of BAMM to capture extinction from extant phylogenies. Also, the speciation rates were near constant and lower for the conservative taxonomy phylogeny, as would be expected given that the number of species was ~ half that of the full tree (Table S5).

**Table S5.** Comparison of rates of speciation and extinction under alternate topologies. The rates for each node were extracted using the *getCladeRates* function in *BAMMtools*, specifying the lemur and lorisiform nodes. While BAMM allows heterogeneous rates among lineages and rates are branch-specific, there was no evidence of a rate shift between lemurs and lorisiforms. Therefore the rates for each clade are similar because a single diversification regime fits the whole strepsirrhine tree.

| **Topology** | **Clade** | **speciation rate** | **5% quantile** | **95% quantile** | **extinction rate** | **5% quantile** | **95% quantile** |
| --- | --- | --- | --- | --- | --- | --- | --- |
| MCC tree | Lemurs | 0.15 | 0.11 | 0.20 | 0.07 | 0.02 | 0.14 |
|  | Lorisiforms | 0.14 | 0.10 | 0.20 | 0.07 | 0.01 | 0.21 |
| alternate *Megaladapis* | Lemurs | 0.15 | 0.11 | 0.19 | 0.07 | 0.02 | 0.13 |
|  | Lorisiforms | 0.14 | 0.10 | 0.19 | 0.07 | 0.01 | 0.14 |
| Extant-only | Lemurs | 0.16 | 0.12 | 0.22 | 0.09 | 0.03 | 0.16 |
|  | Lorisiforms | 0.16 | 0.11 | 0.22 | 0.09 | 0.02 | 0.16 |
| Conservative taxonomy | Lemurs | 0.07 | 0.06 | 0.10 | 0.02 | 0.001 | 0.05 |
|  | Lorisiforms | 0.08 | 0.06 | 0.10 | 0.02 | 0.001 | 0.06 |

**Diversification rate heterogeneity from MEDUSA**

The MEDUSA algorithm found no evidence of a rate shift between lemurs and lorisiforms on any of the phylogenies, including 588 trees from the posterior distribution, when the ΔAICc threshold for model improvement with more complex models was set to ≥ 7 (MCC tree, net diversification rate=0.065, 95% CI = 0.05-0.08, extinction/speciation rates=0.63, 95% CI=0.51-0.72). This threshold was determined based on the lowest ΔAICc values that correctly rejected more complex models for 1000 trees simulated under a single constant-rate diversification regime with a frequency of ~97%. Note that the default ΔAICc threshold determined by MEDUSA for a tree with 120 tips was 5.12. Using the ΔAICc threshold of 5.12, MEDUSA found evidence for one rate shift in 8.7% of simulated constant-rate trees, and with ΔAICc threshold of 6, MEDUSA found one rate shift in 5.7% of trees.

**Diversification dynamics estimated using RPANDA**

RPANDA incorporates a diverse, flexible set of diversification models including constant speciation and/or extinction rates as well as time-varying models in which speciation and extinction can be linear or exponential functions of time. Further, the effects of environmental predictor variables on diversification rates can be tested. I compared the fit of pure-birth time-constant and time-varying models as well as time-constant and time-varying birth-death models to three trees: 1) the full strepsirrhine tree, 2) the lemur tree, 3) the lorisiform tree. I used multimodel inference and model averaging on these results to derive the diversification rate estimates presented in the main text (Table 1).

The best-fitting models of diversification differed between the lemur subtree and the lorisiform subtree, and the overall signal in the strepsirrhine tree was most likely driven by the signal in the lemur tree (Table S3). For lemurs, the best-fitting model was the pure birth model with exponential change in speciation rate over time (increasing), as it was for the strepsirrhine tree (Table S3a,b). For lorisiforms, however, the best-fitting model was the pure birth model with constant speciation through time (Table S3c). These results reflect subtle heterogeneity in the diversification dynamics of these two clades.

I ran the RPANDA models over a subset of 100 trees from the Bayesian posterior distribution of trees and found support (ΔAICc > 2) for the exponential time-varying pure-birth model compared to the constant-rate pure-birth model in 80% of trees for lemurs and 78% for lorisiforms, while for lemurs there was no support for the time-varying birth-death model over the constant-rate birth-death model (0 trees had ΔAICc > 2 in support of the time-varying birth-death model) and only 1% of lorisiform trees supported the time-varying birth-death model. Over 100 trees, the lemur model-averaged speciation rate was double that of lorisiforms (lemur mean = 0.16 lineages/my, SD = 0.07, lorisiform mean = 0.08 lineages/my, SD = 0.03). Further, while the time-varying exponential rate parameter was negative for lemurs suggesting increasing speciation rates through time, the lorisiform time-varying parameter was closer to zero, and more often positive, suggesting decreasing rates through time (lemur model-averaged mean time-varying rate parameter = -0.012, SD = 0.01, loris model-averaged mean time-varying rate parameter = 0.008, SD = 0.005). These results suggest that lemur and lorisiform diversification dynamics were decoupled, and that the homogeneous diversification rates suggested by the BAMM and MEDUSA analyses may be due to low statistical power to detect rate heterogeneity in the tree with 120 tips.

**Results of tests for Eocene-Oligocene extinction:**

To test the hypothesis that crown strepsirrhines, in particular lemurs, experienced an extinction event at approximately 34 Mya, I used the COMeT model implemented in the R package TESS [19]. This analysis is specifically designed to infer speciation and extinction parameters on phylogenetic trees in which prior probabilities are assigned to hypothesized times of mass extinction events (here set to 34 Mya), the probability of extinction (here set to 0.5, a conservative probability estimate with a wide, flat distribution; results were the same whether the prior probability of extinct was set to 0.1, 0.5, or 0.9), and the number of extinction and speciation rate shift events (here set to 1). The results for the MCC strepsirrhine and lemur-only trees from [3], as well as the strepsirrhine and lemur-only trees from analysis of mitochondrial genomes [8] show no evidence for a mass extinction at the Eocene-Oligocene boundary. The Bayes Factor values for the model of elevated extinction rates 34 Mya and speciation rate change was ≤ 2 (Fig S4), while a Bayes factor value of >6 is considered significant evidence for the model with mass extinction [19, 27]. This indicates that although extinction rates were non-zero, there is no evidence of significant increase in extinction rates during lemur diversification.

**Figure S4.** Results of tests for a mass extinction at 34Mya, based on the COMET model. The model was run on four trees – the whole strepsirrhine tree and the lemur-only tree from [3], as well as the whole strepsirrhine tree and the lemur-only tree from [8]. The top row of panels depicts the estimated net diversification rate (speciation-extinction) through time with the 95% highest posterior density shaded. The bottom row of panels depicts the Bayes factor values comparing the marginal likelihood of a model in which mass extinction occurred compared to a model with no mass extinction at time intervals throughout the age of the phylogeny. Horizontal dashed lines show the Bayes factor levels of 2, 6 and 10, with Bayes factor values greater than 6 considered significant evidence for a mass extinction. In no trees was there significant evidence of a mass extinction event.


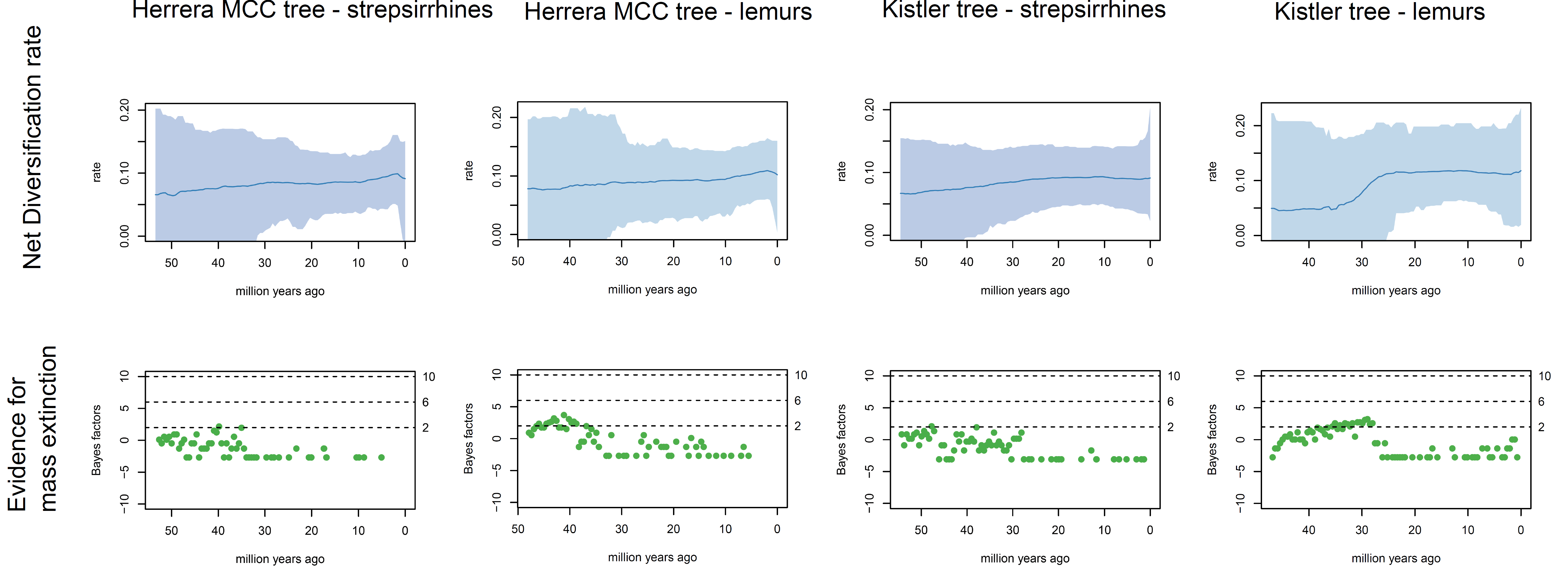


**Posterior predictive model check results:**

To assess the adequacy of the lineage diversification model results, I simulated trees under time-constant one-rate and two-rate models and under a time-varying one-rate model, and then compared tree characteristics between the simulated and observed trees. The results suggest that trees simulated under all three models have similar characteristics to the observed tree (Fig. S3), except that the observed tree is more unbalanced (beta statistic = -0.95) than the trees simulated under the three models (>95% of the trees from each model were more balanced, beta > 0, than the observed trees). This suggests that all three models of macroevolution produce similarly structured trees, but the higher imbalance of the observed tree suggests greater heterogeneity in diversification among lineages than expected given the model parameters fit to the observed tree. Further, trees simulated under one- or two-rate time-constant models had gamma statistics within the range of the observed tree, while ~85% of the trees simulated under the time-varying (increasing) speciation rate had lower gamma values than the observed tree (1.93). This last result was especially surprising because the gamma statistic should detect temporal heterogeneity in node concentration, and with trees simulated to have increasing speciation rates, the nodes should be concentrated towards the tips, rather than towards the root as in decreasing speciation rates. Instead, the time-constant birth-death models produced gamma statistics more similar to the observed tree than the time-varying models. Current models of diversification may have low power to detect subtle heterogeneity among lineages, as observed in the strepsirrhine tree.

**Phenotypic evolution:**

The posterior probability of one diversification rate shift was higher than the probability of zero, two or three rate shifts for the MCC tree under the time-varying and time-constant speciation models and for the tree with the alternate placement of the genus *Megaladapis*, but not for the extant-only or the conservative taxonomy trees (Table S6). These results indicate that the phenotypic evolutionary dynamics inferred with the subfossil species included in the tree differ from those inferred with only extant taxa, suggesting the evolutionary trajectories of the subfossil lemurs were unique compared to living species. When examining the posterior probability of branch-specific rate shifts given the branch-specific prior probability of a rate shift given the branch length (higher prior probability of a shift on longer branches than short branches), there is evidence of a rate shift on the lineages of the lemur genera *Daubentonia* and *Hapalemur* (Figure S5). There was significant divergence in body mass of the living *Daubentonia madagascariensis* from the larger extinct *D. robustus*, and there was a shift towards smaller body mass in the genus *Hapalemur* compared to other lemurids (Fig. S5).

The macroevolutionary models implemented in BAMM include time-varying and time-constant phenotypic evolutionary rates. With the MCC phylogeny, the posterior probabilities of the time-varying and time-constant models were nearly identical, suggesting they cannot be distinguished with this phylogeny (Fig. S6). Thus a constant-rate phenotypic evolutionary rate model fits the phylogeny as well as a time-varying model. In contrast, using the maximum likelihood algorithms implemented in the *Geiger* package function *fitContinuous*, there was clear and decisive evidence supporting the early burst model for body mass evolution over other models (Table S7). The early burst model consistently had the lowest AICc values of all models compared, and the delta AICc for the early burst model was 0 for all 100 lemur trees, indicating it was the best model in each of 100 trees.

**Fig. S5**. Phylogenetic tree with branch lengths rescaled according to the Bayes factor of a branch-specific shift in trait evolutionary rates rate. The longer black branches have higher probability of a rate shift (Bayes factors > 3), while short grey branches have a low probability of a shift (Bayes factor < 3). These results support a rate shift on the branch leading to *Daubentonia* because the living species is significantly divergent in body size from its extinct sister species. There are also rate shifts on the lineage leading to *Lemur* / *Hapalemur* and *Archaeoindris*, illustrating the independent shifts in body mass evolution across the tree.


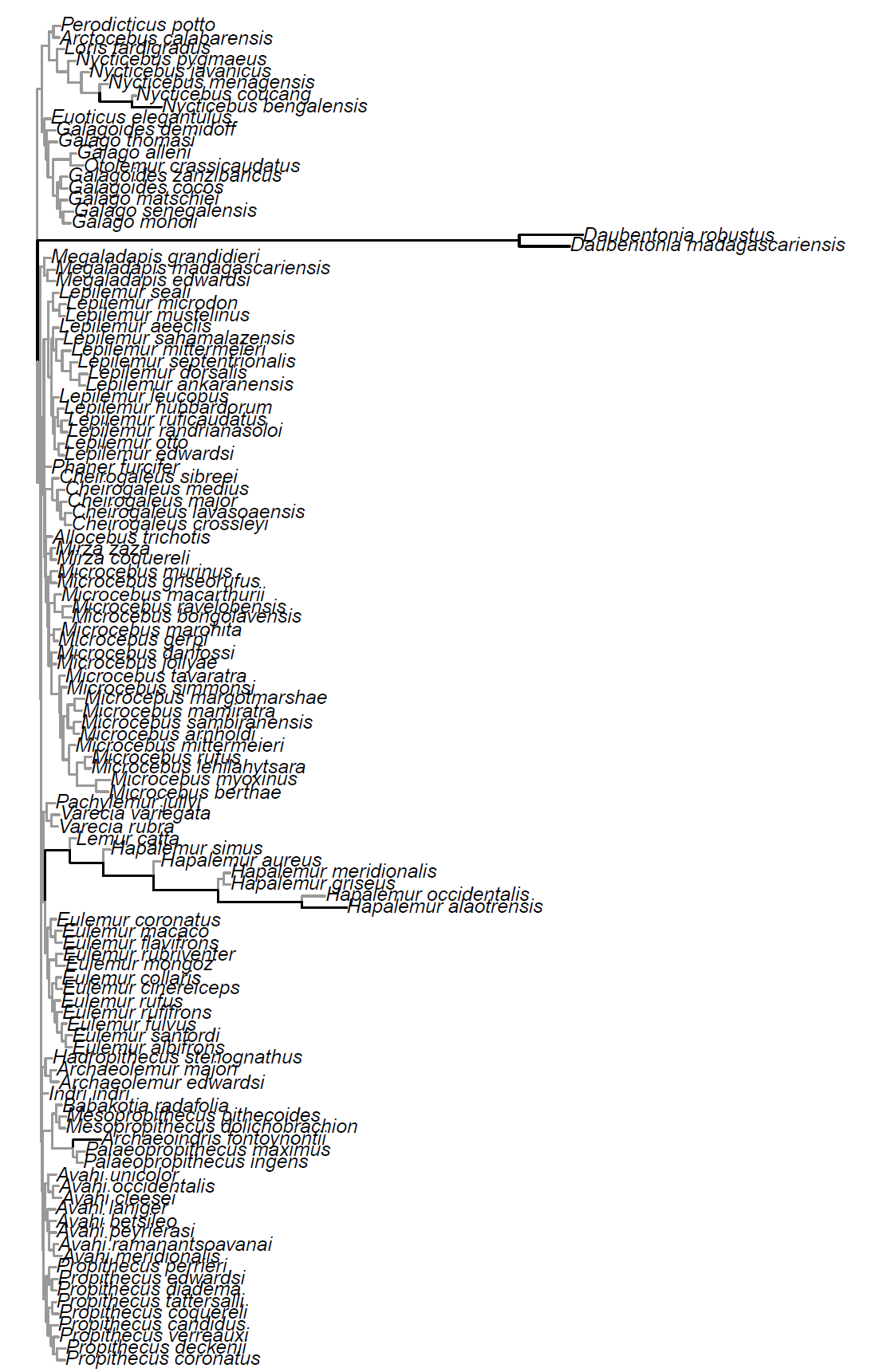


**Fig. S6.** Density plot of posterior distribution of likelihoods from time-varying and time-constant BAMM analyses of phenotypic (natural log body mass) evolutionary rates. The distributions are almost identical, indicating the two models have nearly identical probabilities.


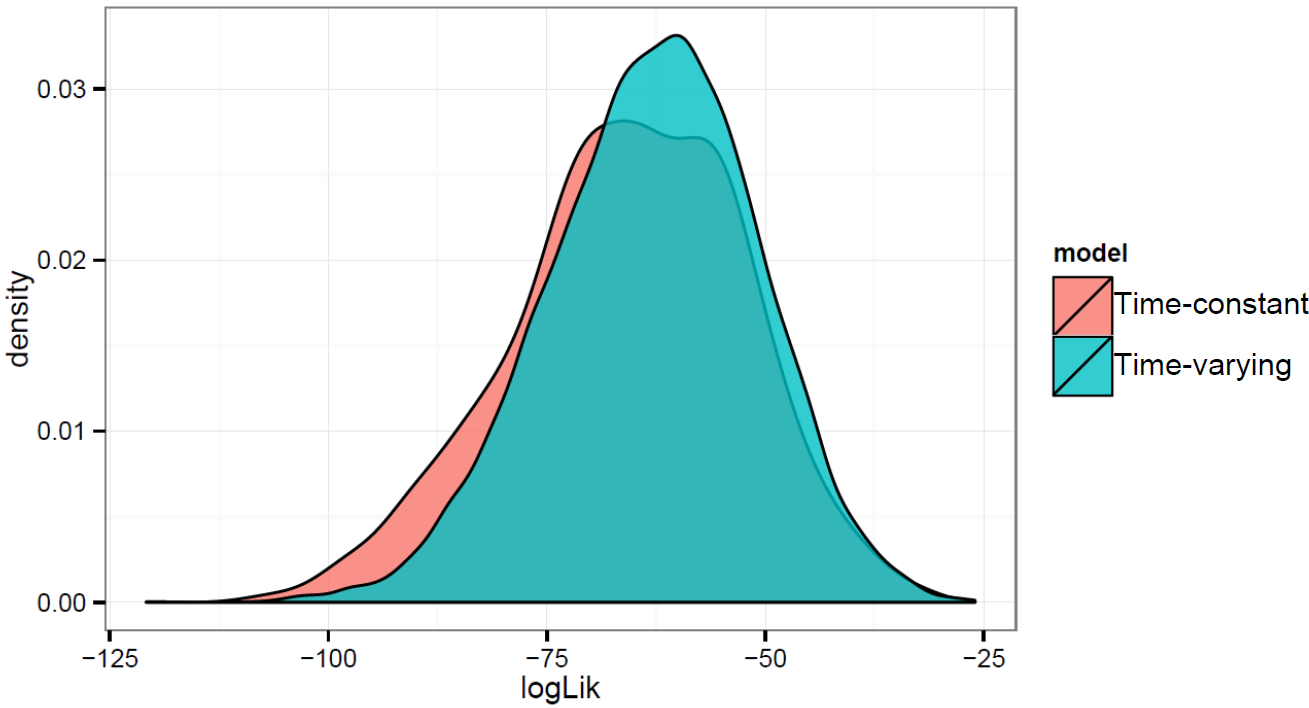


**Table S6.** The posterior probabilities of zero, one, two, or three phenotypic evolutionary rate shifts on four alternate strepsirrhine phylogenies, and for the MCC tree, under time-varying and time-constant speciation rate models. For the topologies with full extant and extinct species, the posterior probability of one shift is higher than for zero shifts. For extant-only and the conservative taxonomy phylogenies, the probability of zero or one shift are similar.

| # shifts | MCC tree, time-varying | MCC tree, time-constant | Alternate *Megaladapis* tree, time-varying | Extant-only tree, time-varying | Conservative taxonomy tree, time-varying |
| --- | --- | --- | --- | --- | --- |
| 0 | 0.16 | 0.16 | 0.15 | 0.44 | 0.30 |
| 1 | 0.41 | 0.41 | 0.41 | 0.32 | 0.38 |
| 2 | 0.26 | 0.26 | 0.27 | 0.16 | 0.21 |
| 3 | 0.11 | 0.11 | 0.11 | 0.06 | 0.08 |
| 4 | 0.04 | 0.04 | 0.004 | 0.02 | 0.02 |

**Table S7.** The means and standard deviations of delta AICc values for each of eight macroevolutionary models of trait evolution across 100 trees for all strepsirrhines (top two rows) and for lemurs only (bottom two rows). The early burst model (EB, bold) had the lowest delta AICc scores for all model comparisons, indicating it was the better model in almost all comparisons for the strepsirrhine tree, and in all comparisons for the lemur tree. The next best fitting model, Brownian motion with a trend, had a mean delta AICc of 3-4, indicating it was significantly worse with a delta AICc threshold of > 2 considered significant [44]. BM = Brownian motion, EB = early burst, OU = Ornstein-Uhlenbeck, white noise = no phylogenetic effect.

|  | model | BM | Drift | **EB** | kappa | lambda | OU | *Trend* | white noise |
| --- | --- | --- | --- | --- | --- | --- | --- | --- | --- |
| All Strepsirrhines | mean | 25.37 | 27.31 | **2.40** | 25.78 | 25.19 | 27.35 | *3.26* | 330.84 |
|  | sd | 13.35 | 13.36 | **15.15** | 6.49 | 7.84 | 12.23 | *14.38* | 18.20 |
| Lemurs | mean | 35.09 | 37.03 | **0.00** | 37.22 | 37.16 | 37.22 | *4.33* | 326.86 |
|  | sd | 5.29 | 5.29 | **0.00** | 5.29 | 5.40 | 5.29 | *1.72* | 5.56 |

**Testing the adaptive phenotypic divergence hypothesis**

I used two approaches to test for a significant adaptive relationship between body mass and the diet and activity pattern niche of lemurs and lorisiforms: a PGLS approach and a generalized OU model approach.

PGLS results:

I fit regression models including different evolutionary rate models to test for an effect of niche categories on body mass (Fig. S7). I found that all regression models of niche categories on body mass evolution (Brownian motion, Ornstein-Uhlenbeck, Pagel’s lambda, and Blomberg’s g) explained the data significantly better than the null models (log likelihood ratio tests of each model versus the null model, ratio >29 for all tests, p <0.0001 for all models). The OU alpha parameter (strength of pull towards the optimum) was low (0.0015), indicating a weak constraint around optimal body mass. Pagel’s lambda was 0.9998, indicating body mass evolved similar to a BM model. The Blomberg’s g parameter was 2.84, indicating a deceleration in body mass evolution over time. The Blomberg’s g model and the OU model had similar fits to the data (AICc weights = 0.48 and 0.38 respectively), while the Pagel and BM models had lower relative support (AICc weights = 0.13 and 0.01, respectively). The model-averaged coefficients reveal that the effect of diurnal omnivory on body size includes zero (95% confidence intervals of model-averaged mean based on unconditional model-averaged standard errors = -0.80 – 0.95), reflected in the non-significant effects found in each model (p > 0.10). Nocturnal folivores and nocturnal omnivores, however, had lower body masses than the mean (coefficient for nocturnal folivores 95% CI=-1.33 – -3.50, coefficient for nocturnal omnivores 95% CI=-2.27 – -4.88). These results support the early burst prediction that lineages co-evolved body mass with diet and activity niches, and that the rate of body mass evolution decelerated over time.

**Fig. S7.** Box plots illustrating the body mass (natural log transformed) of strepsirrhines in each of four niche categories according to discrete states of diet and activity pattern (n=119 species). These data were used to test for adaptive relationships between niche and body size. The boxes enclose the first through third quartiles, the black bar represents the median, and whiskers extend to 1.5 times the interquartile range. Points are beyond 1.5 times the interquartile range.


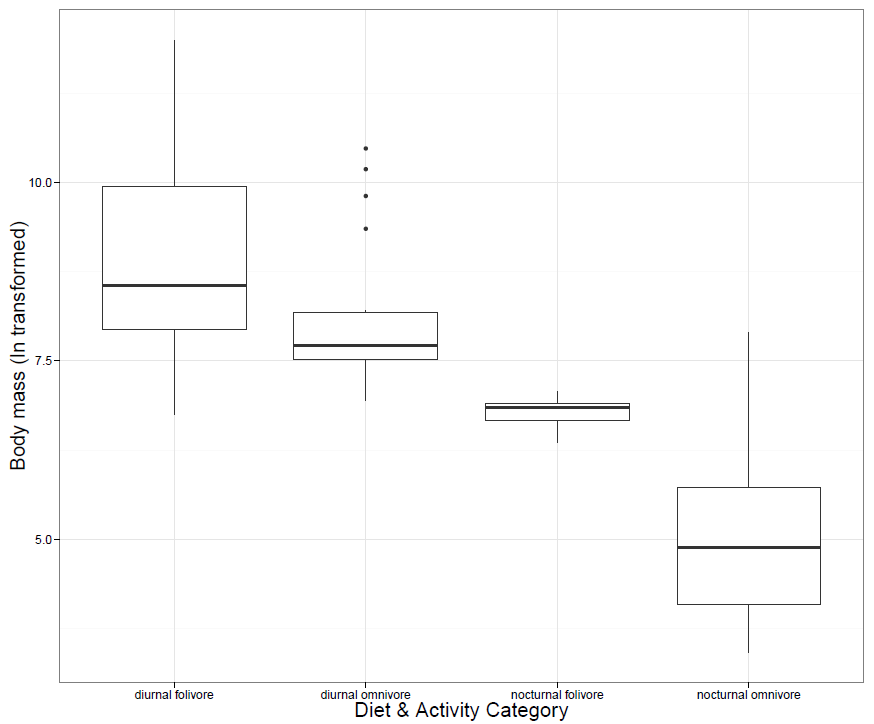


Assessing the adequacy of the models using the R package *arbutus*, I found that the data significantly departed from the expectations of all four models in the test statistics S_var_ and S_HGT_ (Table S8), suggesting that, in all models, the evolution of body mass has greater change on long branches than expected given the model parameters, and that there is greater change on younger nodes than older nodes (Fig. S8). Further, in the best fitting model (Blomberg’s g, which suggests an early burst pattern of body mass evolution), the data significantly departed from the simulated distribution for the C_var_ and D_CDF_ statistic, representing un-modeled heterogeneity in trait evolution. Thus, incorporating rate heterogeneity across the tree is essential for this dataset, as in the BAMM analyses above and the *OUwie* results below.

**Fig. S8.** Assessments of model adequacy by posterior predictive checks. Traits were simulated (n=1000) on trees rescaled to four models of trait evolution: Brownian motion, Pagel’s lambda, Ornstein-Uhlenbeck, and Blomberg’s g, based on phylogenetic generalized least squares regression model results. Black distributions are based on the simulated traits and the orange vertical line is the empirical value. The test statistics were: the mean of the squared contrasts (M_sig_) which indicates if rates are over- or underestimated; the coefficient of variation of absolute contrasts (C_var_) which assesses rate heterogeneity across the tree; the slope of the absolute contrasts against expected variance (S_var_), where a negative slope indicates more evolution per unit time on short than long branches, and a positive relationship may reflect more evolution on long branches than expected; the slope of the linear model fitted to the absolute contrasts against the weighted average value for each node based on evolutionary distance (S_ASR_); the slope of linear model of the absolute contrasts and the node height (node-height test, S_HGT_) which is a test to detect early bursts of trait evolution akin to adaptive radiation theory where positive relationships indicate that greater evolutionary changes occurred at younger nodes than older nodes, and a negative relationship represents greater change in older nodes than younger nodes; the D statistic of the Kolmolgorov-Smirnov test (D_CDF_) to test if the distribution of the contrasts deviates from a normal distribution of mean 0 and standard deviation equal to the root of the mean of squared contrasts, which represents departures of contrasts from normality as seen in jump-diffusion evolution where bursts of rapid evolution occurred heterogeneously across the tree.


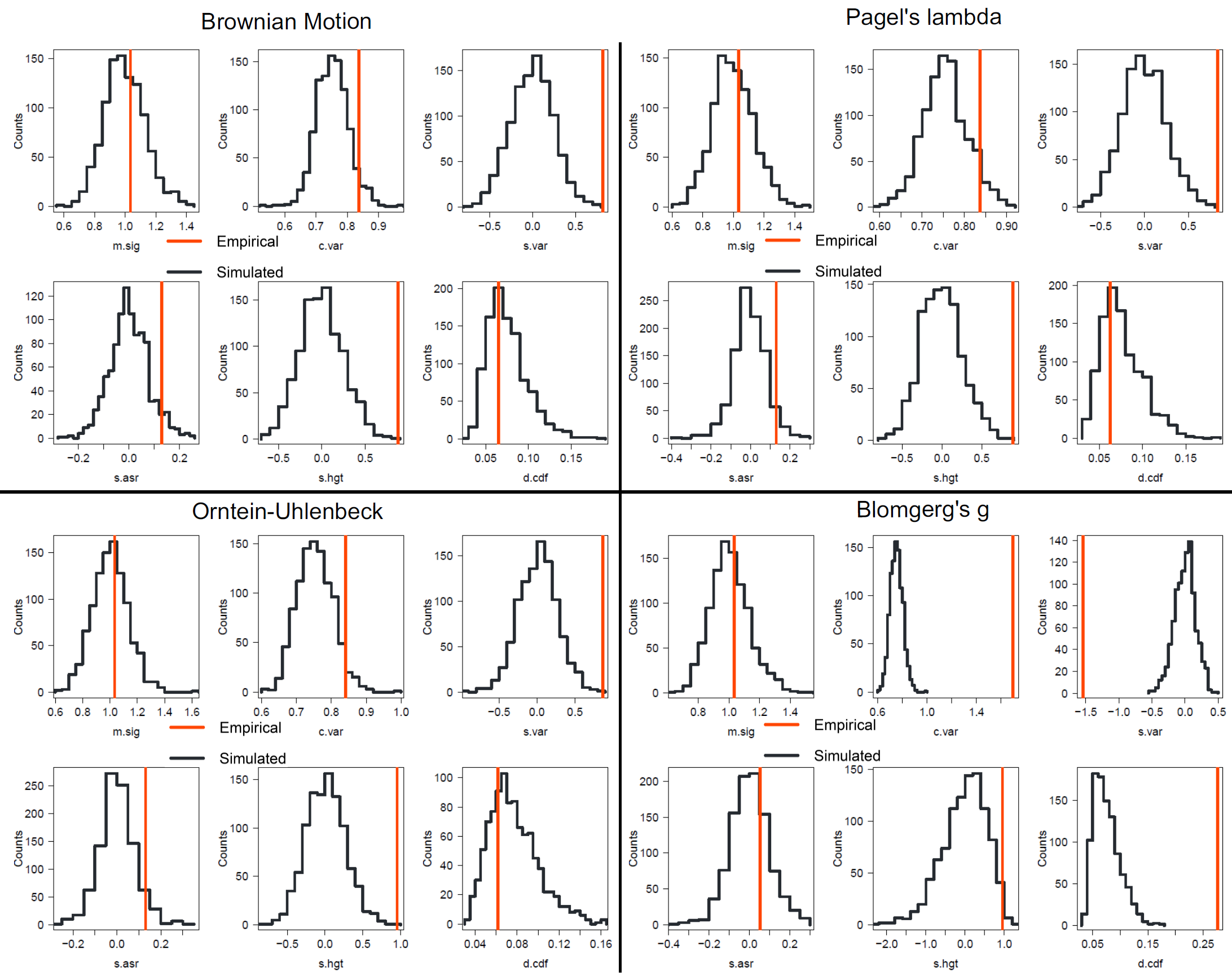


**Table S8.** Assessing model adequacy of trait-niche evolutionary relationships. *P* values are given for tests of significant deviation of observed from expected test statistics under evolutionary model expectations from phylogenetic generalized least-squared regressions fitted for four models of trait evolution (Brownian motion, BM, Pagel’s lambda, Ornstein-Uhlenbeck, OU, and Blomberg’s g). The test statistics were: the mean of the squared contrasts (M_sig_) which indicates if rates are over- or underestimated; the coefficient of variation of absolute contrasts (C_var_) which assesses rate heterogeneity across the tree; the slope of the absolute contrasts against expected variance (S_var_), where a negative slope indicates more evolution per unit time on short than long branches, and a positive relationship may reflect more evolution on long branches than expected; the slope of the linear model fitted to the absolute contrasts against the weighted average value for each node based on evolutionary distance (S_ASR_); the slope of linear model of the absolute contrasts and the node height (node-height test, S_HGT_) which is a test to detect early bursts of trait evolution akin to adaptive radiation theory where positive relationships indicate that greater evolutionary changes occurred at younger nodes than older nodes, and a negative relationship represents greater change in older nodes than younger nodes; the D statistic of the Kolmolgorov-Smirnov test (D_CDF_) to test if the distribution of the contrasts deviates from a normal distribution of mean 0 and standard deviation equal to the root of the mean of squared contrasts, which represents departures of contrasts from normality as seen in jump-diffusion evolution where bursts of rapid evolution occurred heterogeneously across the tree.

| **Model** | **M_sig_** | **C_var_** | **S_var_** | **S_ASR_** | **S_HGT_** | **D_CDF_** |
| --- | --- | --- | --- | --- | --- | --- |
| Brownian motion | 0.73 | 0.10 | 0.00 | 0.09 | 0.00 | 0.82 |
| Pagel’s lambda | 0.77 | 0.14 | 0.00 | 0.09 | 0.00 | 0.64 |
| Ornstein-Uhlenbeck | 0.75 | 0.07 | 0.00 | 0.10 | 0.00 | 0.75 |
| Blomberg’s g | 0.71 | 0.00 | 0.00 | 0.61 | 0.04 | 0.00 |

**Results of state-dependent generalized OU models with maximum likelihood ancestral character state estimates and over stochastic maps of niches**

The results of *OUwie* analyses using the ancestral state estimates from the maximum likelihood analysis of niche evolution are given in Table S9. The OUMV was the best fitting model, followed by the OUMVA model.

To assess the effects of uncertainty in the phylogeny and estimates of character states at internal nodes, I used stochastic character mapping (*make.simmap* function in *phytools*) to map the characters on the each of 100 trees 100 times, randomly sampled 100 of these 1000 character mappings, and ran the OUwie analyses as described in the supporting information methods on each of the 100 stochastic maps. I summarized the results as the AICc and ΔAICc for each model in each run, and compared these AICc and ΔAICc values among models across the 100 mappings. The results support the state-dependent OU models over a single Browian motion or Ornstein-Uhlenbeck model in all cases (Table S10). The OUMA model had the lowest AICc values and lowest ΔAICc values among models, supporting results based on the single maximum likelihood estimates of ancestral states, in which the OUMVA and OUMV models had the best fit to the data. Note that I did not test the OUMVA models with the stochastic mappings because this is the most parameter-rich model which did not converge on optimal solutions in all SMs.

**Table S9.** Complete results of the state-dependent phenotypic evolution analyses comparing generalized Brownian motion and Ornstein-Uhlenbeck models. The likelihood of the following seven models was assessed using the R package *OUwie* [42, 49]: a single Brownian motion model (BM), a single Ornstein-Uhlenbeck model (OU), a different BM rate for each adaptive zone state (state-dependent BM, BMS), a different OU model for each state in which each adaptive zone has its own optimal body mass (state-dependent OU, OUM), different OU models in which each state has a unique optimal body mass and a unique constraint parameter pulling the trait value towards the optima (state-dependent OU, OUMA), different OU models in which each state has a unique optimal body mass and evolutionary rate parameter (state-dependent OU, OUMV), and different OU models for each state in which each state has a unique optimal body mass, constraint parameter, and evolutionary rate (state-dependent OU, OUMVA. Columns give parameter values for each adaptive zone as follows: DF=diurnal folivore, DO=diurnal omnivore, NF=nocturnal folivore, NO=nocturnal omnivore. UCI=upper 95% confidence interval, LCI=lower 95% confidence interval. A) The estimated values of the evolutionary rate parameter (sigma squared) and associated standard errors (SE) for each adaptive zone. Note that only in the OUMV and OUMVA models can the sigma squared vary among states. B) The estimated values of the optimal body mass (theta) and standard errors for each state with each model. Note that only in the OUM, OUMA, OUMV, and OUMVA models can optima vary among states. With the BM and BMS models, the theta values given are the estimated root states of the BM model. C) The estimated values of the constraint parameter (alpha) for each state with each model. Note that only in the OU models is there an alpha parameter, and only in the OUMA and OUMVA models does alpha vary among states.

| **A)** | **Evolutionary rate parameter (sigma squared)** | | | | |  |  |  |
| --- | --- | --- | --- | --- | --- | --- | --- | --- |
| **Model** | **DF** | **DF SE** | **DO** | **DO SE** | **NF** | **NF SE** | **NO** | **NO SE** |
| BM | 0.0307 | 0.1296 | 0.0307 | 0.1296 | 0.0307 | 0.1296 | 0.0307 | 0.1296 |
| OU | 0.03078 | 0.13 | 0.03078 | 0.13 | 0.03078 | 0.13 | 0.03078 | 0.13 |
| BMS | 0.078 | 0.279 | 0.018 | 0.346 | 0.004 | 0.3 | 0.025 | 0.21 |
| OUM | 0.023 | 0.13 | 0.023 | 0.13 | 0.023 | 0.13 | 0.023 | 0.13 |
| OUMA | 0.021 | 0.13 | 0.021175 | 0.1296 | 0.021175 | 0.1296 | 0.021175 | 0.1296 |
| OUMV | 0.036 | 0.28 | 0.019 | 0.35 | 0.005 | 0.29 | 0.025 | 0.21 |
| OUMVA | 0.03 | 0.25 | 0.02 | 0.34 | 0.005 | - | 0.02 | 0.20 |
|  |  |  |  |  |  |  |  |  |
|  |  |  |  |  |  |  |  |  |
| **B)** | **Body mass optima parameter (theta)** | | | |  |  |  |  |
| **Model** | **DF** | **DF SE** | **DO** | **DO SE** | **NF** | **NF SE** | **NO** | **NO SE** |
| BM | 7.148 | 0.57299 | 7.148 | 0.57299 | 7.148 | 0.57299 | 7.148 | 0.57299 |
| OU | 7.148 | 0.57329 | 7.148 | 0.57329 | 7.148 | 0.57329 | 7.148 | 0.57329 |
| BMS | 4E-9 | 4E-10 | 1E-9 | 1E-10 | 7E-10 | 6E-11 | 3E-8 | 2E-9 |
| OUM | 11.61 | 0.87 | 9.25 | 1.28 | 4.37 | 1.50 | 5.64 | 0.56 |
| OUMA | 952.57 | 140.02 | 9.48 | 0.99 | 7.20 | 0.82 | 5.96 | 0.52 |
| OUMV | 11.65 | 1.03 | 9.42 | 1.36 | 4.65 | 1.44 | 5.71 | 0.60 |
| OUMVA | 39698 | 6669 | 9.42 | 1.04 | 7.34 | 0.77 | 6.01 | 0.56 |
|  |  |  |  |  |  |  |  |  |
|  |  |  |  |  |  |  |  |  |
| **C)** | **Evolutionary constraint parameter (alpha)** | | | | |  |  |  |
| **Model** | **DF** | **DF SE** | **DO** | **DO SE** | **NF** | **NF SE** | **NO** | **NO SE** |
| BM | - | - | - | - | - | - | - | - |
| OU | 2E-09 | - | 2E-09 | - | 2E-09 | - | 2E-09 | - |
| BMS | - | - | - | - | - | - | - | - |
| OUM | 2E-09 | 383.399 | 2E-09 | 383.399 | 2E-09 | 383.399 | 2E-09 | 383.399 |
| OUMA | 2E-09 | 29.58 | 1E-6 | 21.11 | 3E-6 | 20.89 | 2E-6 | 21.06 |
| OUMV | 2E-09 | - | 2E-09 | - | 2E-09 | - | 2E-09 | - |
| OUMVA | 2E-09 | 152.37 | 7E-5 | - | 2E-3 | - | 8E-5 | - |

**Table S10.** Results of model comparisons using OUwie iterated over 100 trees with stochastic character mappings to assess the impact of phylogenetic and ancestral niche uncertainty. The results suggest that the best model (lowest AICc and deltaAICc) was the OUMA model (bold), in which each state has a unique optimum and strength of selection around the optimum. IQR = interquartile range.

|  | model | BM1 | BMS | OU1 | OUM | **OUMA** | OUMV |
| --- | --- | --- | --- | --- | --- | --- | --- |
| delta AICc | mean | 90.83176 | 82.87778 | 92.82926 | 88.13071 | **8.54546** | 82.87778 |
|  | IQR | 13.63524 | 9.698269 | 13.63533 | 12.76415 | **13.00823** | 9.698269 |
| AICc | mean | 189.1281 | 181.1741 | 191.1256 | 186.4271 | **106.8418** | 181.1741 |
|  | IQR | 9.892902 | 16.64042 | 9.892903 | 16.43995 | **18.42691** | 16.64042 |

**Fig. S9**. Strepsirrhine phylogeny illustrating the distribution of body mass (bars next to tips, scale at the bottom, natural log transformed) and adaptive zones of diet and activity pattern (colored circles next to tips). The evolution of adaptive zones was estimated at internal nodes (colored pie charts on nodes representing the proportional probability of each state) and the best estimate at each node was assigned to the descendant lineages (colored branches). This tree was used for inferences of state-dependent trait evolution models.


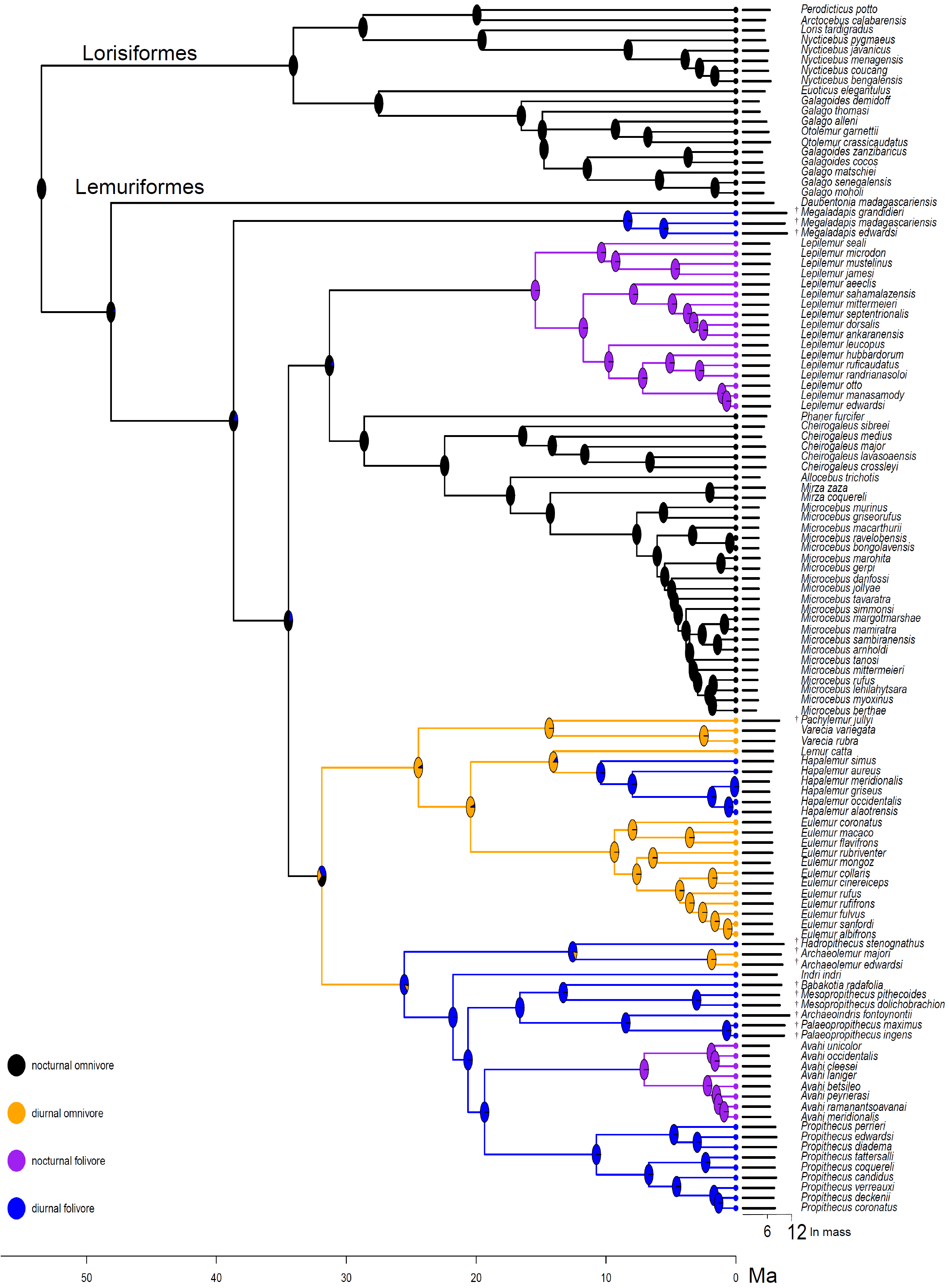


**Supplementary Information Files**

**File S1.** Maximum clade credibility tree from phylogenetic analysis of total evidence dataset inferring divergence times under the fossilized birth-death process. The file is in Newick format and can be saved as a .tre file, which can be opened using tree viewing software such as FigTree, with packages for R, or as a text file with the tree in Newick format.

((((((((((((((Avahi_meridionalis:0.941644,Avahi_ramanantsoavanai:0.941644):0.462545,Avahi_peyrierasi:1.404188):0.151095,Avahi_betsileo:1.555283):0.692846,Avahi_laniger:2.248129):4.946231,((Avahi_cleesei:1.648331,Avahi_occidentalis:1.648331):0.288684,Avahi_unicolor:1.937015):5.257345):11.57829,Indri_indri:18.77265):1.701356,(((((Propithecus_coronatus:1.263577,Propithecus_deckenii:1.263577):0.358923,Propithecus_verreauxi:1.6225):2.838879,Propithecus_candidus:4.461379):2.086907,(Propithecus_coquereli:2.255128,Propithecus_tattersalli:2.255128):4.293159):4.063464,((Propithecus_diadema:2.893823,Propithecus_edwardsi:2.893823):1.766948,Propithecus_perrieri:4.660771):5.95098):9.862259):2.351881,(((Palaeopropithecus_ingens:0.785461,Palaeopropithecus_maximus:0.785461):9.551098,Archaeoindris_fontoynontii:10.336559):7.782525,((Mesopropithecus_dolichobrachion:3.248072,Mesopropithecus_pithecoides:3.248072):11.43192,Babakotia_radafolia:14.68):3.439088):4.706806):4.06392,((Archaeolemur_edwardsi:2.004629,Archaeolemur_majori:2.004629):11.41026,Hadropithecus_stenognathus:13.41488):13.47493):6.358057,(((((((((Eulemur_albifrons:0.622193,Eulemur_sanfordi:0.622193):0.980267,Eulemur_fulvus:1.60246):0.948441,Eulemur_rufifrons:2.550901):1.011279,Eulemur_rufus:3.56218):0.809821,(Eulemur_cinereiceps:1.834229,Eulemur_collaris:1.834229):2.537772):3.39312,(Eulemur_mongoz:6.493713,Eulemur_rubriventer:6.493713):1.271408):1.796564,((Eulemur_flavifrons:3.599981,Eulemur_macaco:3.599981):4.523215,Eulemur_coronatus:8.123196):1.438489):11.59914,(((((Hapalemur_alaotrensis:0.584997,Hapalemur_occidentalis:0.584997):1.407296,(Hapalemur_griseus:0.109589,Hapalemur_meridionalis:0.109589):1.882704):6.327005,Hapalemur_aureus:8.319298):2.560086,Hapalemur_simus:10.87938):3.736577,Lemur_catta:14.61596):6.544866):4.223573,((Varecia_rubra:2.494545,Varecia_variegata:2.494545):12.45531,Pachylemur_jullyi:14.94985):10.43454):7.863467):2.704021,(((((((((((((((((Microcebus_berthae:1.912855,Microcebus_myoxinus:1.912855):0.274378,(Microcebus_lehilahytsara:1.85168,Microcebus_rufus:1.85168):0.335553):0.939254,Microcebus_mittermeieri:3.126487):0.353023,Microcebus_tanosi:3.479509):0.325091,((Microcebus_arnholdi:1.49376,Microcebus_sambiranensis:1.49376):1.276557,(Microcebus_mamiratra:0.929594,Microcebus_margotmarshae:0.929594):1.840724):1.034283):0.30796,Microcebus_simmonsi:4.112559):0.629108,Microcebus_tavaratra:4.741668):0.326233,Microcebus_jollyae:5.0679):0.190739,Microcebus_danfossi:5.25864):0.574725,(Microcebus_gerpi:1.220119,Microcebus_marohita:1.220119):4.613245):0.686446,((Microcebus_bongolavensis:0.516262,Microcebus_ravelobensis:0.516262):3.094884,Microcebus_macarthurii:3.611146):2.908664):1.712404,(Microcebus_griseorufus:5.996406,Microcebus_murinus:5.996406):2.235808):7.041265,(Mirza_coquereli:2.166752,Mirza_zaza:2.166752):13.10673):3.160738,Allocebus_trichotis:18.43422):5.257292,((((Cheirogaleus_crossleyi:7.041186,Cheirogaleus_lavasoaensis:7.041186):5.231443,Cheirogaleus_major:12.27263):2.624872,Cheirogaleus_medius:14.8975):2.390103,Cheirogaleus_sibreei:17.2876):6.403905):6.223163,Phaner_furcifer:29.91467):2.713433,((((((Lepilemur_edwardsi:0.731071,Lepilemur_manasamody:0.731071):0.382438,Lepilemur_otto:1.113508):6.356502,((Lepilemur_randrianasoloi:2.914231,Lepilemur_ruficaudatus:2.914231):2.38385,Lepilemur_hubbardorum:5.298081):2.17193):2.73864,Lepilemur_leucopus:10.20865):2.029089,(((((Lepilemur_ankaranensis:2.597243,Lepilemur_dorsalis:2.597243):0.766956,Lepilemur_septentrionalis:3.364199):0.468164,Lepilemur_mittermeieri:3.832363):1.247201,Lepilemur_sahamalazensis:5.079564):3.161298,Lepilemur_aeeclis:8.240862):3.996877):3.818404,(((Lepilemur_jamesi:4.812755,Lepilemur_mustelinus:4.812755):4.793359,Lepilemur_microdon:9.606114):1.100804,Lepilemur_seali:10.70692):5.349225):16.57196):3.323783):4.181511,((Megaladapis_edwardsi:6.111134,Megaladapis_madagascariensis:6.111134):2.942214,Megaladapis_grandidieri:9.053349):31.08005):9.448068,(Daubentonia_madagascariensis:3.540792,Daubentonia_robustus:3.540792):46.04067):4.829625,(((((Galago_moholi:1.631194,Galago_senegalensis:1.631194):13.374243,Otolemur_crassicaudatus:15.005437):2.00748,Galagoides_demidoff:17.01292):10.65479,Euoticus_elegantulus:27.667706):6.906197,((((Nycticebus_bengalensis:1.788191,Nycticebus_coucang:1.788191):7.142447,Nycticebus_pygmaeus:8.930637):11.420524,Loris_tardigradus:20.35116):9.60736,(Arctocebus_calabarensis:20.82852,Perodicticus_potto:20.82852):9.130003):4.615381):19.837193);

**File S2.** Alternate topology used to investigate the sensitivity of results to the phylogenetic position of *Megaladapis*.

(((((((((((((Avahi_meridionalis:0.941644,Avahi_ramanantsoavanai:0.941644):0.462545,Avahi_peyrierasi:1.404188):0.151095,Avahi_betsileo:1.555283):0.692846,Avahi_laniger:2.248129):4.946231,((Avahi_cleesei:1.648331,Avahi_occidentalis:1.648331):0.288684,Avahi_unicolor:1.937015):5.257345):11.57829,Indri_indri:18.77265):1.701356,(((((Propithecus_coronatus:1.263577,Propithecus_deckenii:1.263577):0.358923,Propithecus_verreauxi:1.6225):2.838879,Propithecus_candidus:4.461379):2.086907,(Propithecus_coquereli:2.255128,Propithecus_tattersalli:2.255128):4.293159):4.063464,((Propithecus_diadema:2.893823,Propithecus_edwardsi:2.893823):1.766948,Propithecus_perrieri:4.660771):5.95098):9.862259):2.351881,(((Palaeopropithecus_ingens:0.785461,Palaeopropithecus_maximus:0.785461):9.551098,Archaeoindris_fontoynontii:10.336559):7.782525,((Mesopropithecus_dolichobrachion:3.248072,Mesopropithecus_pithecoides:3.248072):11.43192,Babakotia_radafolia:14.68):3.439088):4.706806):4.06392,((Archaeolemur_edwardsi:2.004629,Archaeolemur_majori:2.004629):11.41026,Hadropithecus_stenognathus:13.41488):13.47493):6.358057,((((((((((Eulemur_albifrons:0.622193,Eulemur_sanfordi:0.622193):0.980267,Eulemur_fulvus:1.60246):0.948441,Eulemur_rufifrons:2.550901):1.011279,Eulemur_rufus:3.56218):0.809821,(Eulemur_cinereiceps:1.834229,Eulemur_collaris:1.834229):2.537772):3.39312,(Eulemur_mongoz:6.493713,Eulemur_rubriventer:6.493713):1.271408):1.796564,((Eulemur_flavifrons:3.599981,Eulemur_macaco:3.599981):4.523215,Eulemur_coronatus:8.123196):1.438489):11.59914,(((((Hapalemur_alaotrensis:0.584997,Hapalemur_occidentalis:0.584997):1.407296,(Hapalemur_griseus:0.109589,Hapalemur_meridionalis:0.109589):1.882704):6.327005,Hapalemur_aureus:8.319298):2.560086,Hapalemur_simus:10.87938):3.736577,Lemur_catta:14.61596):6.544866):4.223573,((Varecia_rubra:2.494545,Varecia_variegata:2.494545):12.45531,Pachylemur_jullyi:14.94985):10.43454):2,((Megaladapis_edwardsi:6.111134,Megaladapis_madagascariensis:6.111134):2.942214,Megaladapis_grandidieri:9.053349):18.33105):5.863467):2.704021,(((((((((((((((((Microcebus_berthae:1.912855,Microcebus_myoxinus:1.912855):0.274378,(Microcebus_lehilahytsara:1.85168,Microcebus_rufus:1.85168):0.335553):0.939254,Microcebus_mittermeieri:3.126487):0.353023,Microcebus_tanosi:3.479509):0.325091,((Microcebus_arnholdi:1.49376,Microcebus_sambiranensis:1.49376):1.276557,(Microcebus_mamiratra:0.929594,Microcebus_margotmarshae:0.929594):1.840724):1.034283):0.30796,Microcebus_simmonsi:4.112559):0.629108,Microcebus_tavaratra:4.741668):0.326233,Microcebus_jollyae:5.0679):0.190739,Microcebus_danfossi:5.25864):0.574725,(Microcebus_gerpi:1.220119,Microcebus_marohita:1.220119):4.613245):0.686446,((Microcebus_bongolavensis:0.516262,Microcebus_ravelobensis:0.516262):3.094884,Microcebus_macarthurii:3.611146):2.908664):1.712404,(Microcebus_griseorufus:5.996406,Microcebus_murinus:5.996406):2.235808):7.041265,(Mirza_coquereli:2.166752,Mirza_zaza:2.166752):13.10673):3.160738,Allocebus_trichotis:18.43422):5.257292,((((Cheirogaleus_crossleyi:7.041186,Cheirogaleus_lavasoaensis:7.041186):5.231443,Cheirogaleus_major:12.27263):2.624872,Cheirogaleus_medius:14.8975):2.390103,Cheirogaleus_sibreei:17.2876):6.403905):6.223163,Phaner_furcifer:29.91467):2.713433,((((((Lepilemur_edwardsi:0.731071,Lepilemur_manasamody:0.731071):0.382438,Lepilemur_otto:1.113508):6.356502,((Lepilemur_randrianasoloi:2.914231,Lepilemur_ruficaudatus:2.914231):2.38385,Lepilemur_hubbardorum:5.298081):2.17193):2.73864,Lepilemur_leucopus:10.20865):2.029089,(((((Lepilemur_ankaranensis:2.597243,Lepilemur_dorsalis:2.597243):0.766956,Lepilemur_septentrionalis:3.364199):0.468164,Lepilemur_mittermeieri:3.832363):1.247201,Lepilemur_sahamalazensis:5.079564):3.161298,Lepilemur_aeeclis:8.240862):3.996877):3.818404,(((Lepilemur_jamesi:4.812755,Lepilemur_mustelinus:4.812755):4.793359,Lepilemur_microdon:9.606114):1.100804,Lepilemur_seali:10.70692):5.349225):16.57196):3.323783):13.629579,(Daubentonia_madagascariensis:3.540792,Daubentonia_robustus:3.540792):46.04067):4.829625,(((((Galago_moholi:1.631194,Galago_senegalensis:1.631194):13.374243,Otolemur_crassicaudatus:15.005437):2.00748,Galagoides_demidoff:17.01292):10.65479,Euoticus_elegantulus:27.667706):6.906197,((((Nycticebus_bengalensis:1.788191,Nycticebus_coucang:1.788191):7.142447,Nycticebus_pygmaeus:8.930637):11.420524,Loris_tardigradus:20.35116):9.60736,(Arctocebus_calabarensis:20.82852,Perodicticus_potto:20.82852):9.130003):4.615381):19.837193);

File S3. Alternate topology of extant strepsirrhines only, used to investigate the sensitivity of results to the omission of the extinct subfossil lemurs.

(((((((((((Propithecus_coronatus:1.323714,Propithecus_deckenii:1.323714):0.3591151,Propithecus_verreauxi:1.682829):2.878801,Propithecus_candidus:4.561631):2.14273,(Propithecus_coquereli:2.326008,Propithecus_tattersalli:2.326008):4.378353):4.033076,((Propithecus_diadema:2.973346,Propithecus_edwardsi:2.973346):1.794566,Propithecus_perrieri:4.767912):5.969525):8.610843,(((((Avahi_meridionalis:0.907478,Avahi_ramanantsoavanai:0.907478):0.4362694,Avahi_peyrierasi:1.343747):0.1580154,Avahi_betsileo:1.501763):0.6823424,Avahi_laniger:2.184105):4.872181,((Avahi_cleesei:1.60422,Avahi_occidentalis:1.60422):0.2816227,Avahi_unicolor:1.885843):5.170443):12.29199):2.436924,Indri_indri:21.7852):10.098885,(((((((((Eulemur_albifrons:0.6243069,Eulemur_sanfordi:0.6243069):0.9778339,Eulemur_fulvus:1.602141):0.9482927,Eulemur_rufifrons:2.550433):0.9964465,Eulemur_rufus:3.54688):0.7693613,(Eulemur_cinereiceps:1.787726,Eulemur_collaris:1.787726):2.528516):3.310196,(Eulemur_mongoz:6.385281,Eulemur_rubriventer:6.385281):1.241156):1.72074,((Eulemur_flavifrons:3.551795,Eulemur_macaco:3.551795):4.405333,Eulemur_coronatus:7.957128):1.390049):11.08107,(((((Hapalemur_alaotrensis:0.5493957,Hapalemur_occidentalis:0.5493957):1.295634,(Hapalemur_griseus:0.09835965,Hapalemur_meridionalis:0.09835965):1.74667):6.127844,Hapalemur_aureus:7.972873):2.435581,Hapalemur_simus:10.40845):3.645341,Lemur_catta:14.0538):6.37445):4.022895,(Varecia_rubra:2.459535,Varecia_variegata:2.459535):21.9916):7.432947):2.571182,((((((((((((((((Microcebus_berthae:1.80529,Microcebus_myoxinus:1.80529):0.2663717,(Microcebus_lehilahytsara:1.755794,Microcebus_rufus:1.755794):0.3158679):0.8660426,Microcebus_mittermeieri:2.937704):0.623018,((Microcebus_arnholdi:1.409669,Microcebus_sambiranensis:1.409669):1.182927,(Microcebus_mamiratra:0.8786976,Microcebus_margotmarshae:0.8786976):1.713898):0.9681262):0.274725,Microcebus_simmonsi:3.835447):0.6012598,Microcebus_tavaratra:4.436707):0.3065567,Microcebus_jollyae:4.743264):0.2026254,Microcebus_danfossi:4.945889):0.5285908,(Microcebus_gerpi:1.149041,Microcebus_marohita:1.149041):4.325439):0.5847526,((Microcebus_bongolavensis:0.4616303,Microcebus_ravelobensis:0.4616303):2.868032,Microcebus_macarthurii:3.329662):2.72957):1.569119,(Microcebus_griseorufus:5.575392,Microcebus_murinus:5.575392):2.05296):6.657658,(Mirza_coquereli:1.999641,Mirza_zaza:1.999641):12.28637):3.067236,Allocebus_trichotis:17.35325):5.071134,((((Cheirogaleus_crossleyi:6.611177,Cheirogaleus_lavasoaensis:6.611177):5.0215,Cheirogaleus_major:11.63268):2.504014,Cheirogaleus_medius:14.13669):2.276961,Cheirogaleus_sibreei:16.41365):6.010728):6.201051,Phaner_furcifer:28.62543):2.670621,(((((Lepilemur_edwardsi:1.0715591,Lepilemur_otto:1.071559):6.100235,((Lepilemur_randrianasoloi:2.790855,Lepilemur_ruficaudatus:2.790855):2.27814,Lepilemur_hubbardorum:5.068995):2.1028):2.608534,Lepilemur_leucopus:9.780329):1.965831,(((((Lepilemur_ankaranensis:2.50237,Lepilemur_dorsalis:2.50237):0.7363988,Lepilemur_septentrionalis:3.238769):0.4758641,Lepilemur_mittermeieri:3.714633):1.174493,Lepilemur_sahamalazensis:4.889126):2.989041,Lepilemur_aeeclis:7.878167):3.867993):3.696776,((Lepilemur_mustelinus:9.254806,Lepilemur_microdon:9.254806):1.090952,Lepilemur_seali:10.34576):5.097178):15.85312):3.159219):13.666092,Daubentonia_madagascariensis:48.121361):5.343476,((((((((Galago_moholi:1.602478,Galago_senegalensis:1.602478):4.274901,Galago_matschiei:5.877379):5.566416,(Galagoides_cocos:3.675427,Galagoides_zanzibaricus:3.675427):7.768367):3.331193,(Otolemur_crassicaudatus:9.271237,Galago_alleni:9.271237):5.503751):0.1306054,Galago_thomasi:14.90559):1.613652,Galagoides_demidoff:16.51925):10.98078,Euoticus_elegantulus:27.500021):6.579779,((((((Nycticebus_bengalensis:1.617878,Nycticebus_coucang:1.617878):1.180186,Nycticebus_menagensis:2.798064):1.103412,Nycticebus_javanicus:3.901476):4.384445,Nycticebus_pygmaeus:8.285921):11.26217,Loris_tardigradus:19.54809):9.165244,(Arctocebus_calabarensis:19.93828,Perodicticus_potto:19.93828):8.775056):5.366467):19.385037);

**File S4.** Alternate topology used to investigate the sensitivity of results to the omission of species named since 2005, giving a conservative (‘lumping’) taxonomy.

((((((((((((Propithecus_verreauxi:4.56163,Propithecus_candidus:4.561631):2.14273,Propithecus_tattersalli:6.704361):4.033076,Propithecus_diadema:10.737437):8.610843,(Avahi_laniger:7.056286,(Avahi_occidentalis:1.8858427,Avahi_unicolor:1.885843):5.170443):12.29199):1.27589,(((Palaeopropithecus_ingens:0.7025341,Palaeopropithecus_maximus:0.7025341):7.768762,Archaeoindris_fontoynontii:8.471296):8.158945,((Mesopropithecus_dolichobrachion:3.016583,Mesopropithecus_pithecoides:3.016583):10.27482,Babakotia_radafolia:13.2914):3.338838):3.993928):1.161034,Indri_indri:21.7852):3.747126,((Archaeolemur_edwardsi:1.857565,Archaeolemur_majori:1.857565):10.70476,Hadropithecus_stenognathus:12.56233):12.97):6.351759,((((Eulemur_fulvus:7.6264375,(Eulemur_mongoz:6.385281,Eulemur_rubriventer:6.385281):1.241156):1.72074,Eulemur_macaco:9.347177):11.08107,(((Hapalemur_griseus:7.97287365,Hapalemur_aureus:7.972873):2.435581,Hapalemur_simus:10.40845):3.645341,Lemur_catta:14.0538):6.37445):4.022895,(Varecia_variegata:14.369685,Pachylemur_jullyi:14.36969):10.08145):7.432947):2.571182,(((((((((((Microcebus_berthae:1.80529,Microcebus_myoxinus:1.80529):0.2663717,Microcebus_rufus:2.0716619):1.4890606,Microcebus_sambiranensis:3.5607222):0.8759848,Microcebus_tavaratra:4.436707):1.6225255,Microcebus_ravelobensis:6.0592323):1.569119,(Microcebus_griseorufus:5.575392,Microcebus_murinus:5.575392):2.05296):6.657658,Mirza_coquereli:14.286011):3.067236,Allocebus_trichotis:17.35325):5.071134,(Cheirogaleus_major:14.136694,Cheirogaleus_medius:14.13669):8.287689):6.201051,Phaner_furcifer:28.62543):2.670621,((((Lepilemur_edwardsi:7.1717941,Lepilemur_ruficaudatus:7.171795):2.608534,Lepilemur_leucopus:9.780329):1.965831,Lepilemur_septentrionalis:11.7461601):3.696776,(Lepilemur_mustelinus:9.254806,Lepilemur_microdon:9.254806):6.18813):15.85312):3.159219):4.223729,((Megaladapis_edwardsi:5.547562,Megaladapis_madagascariensis:5.547562):2.774558,Megaladapis_grandidieri:8.32212):30.35688):9.442363,(Daubentonia_madagascariensis:3.378941,Daubentonia_robustus:3.378941):44.74242):5.343476,(((((Galago_moholi:1.602478,Galago_senegalensis:1.602478):13.17251,Otolemur_crassicaudatus:14.774988):1.7442574,Galagoides_demidoff:16.51925):10.98078,Euoticus_elegantulus:27.500021):6.579779,((((Nycticebus_bengalensis:1.617878,Nycticebus_coucang:1.617878):6.668043,Nycticebus_pygmaeus:8.285921):11.26217,Loris_tardigradus:19.54809):9.165244,(Arctocebus_calabarensis:19.93828,Perodicticus_potto:19.93828):8.775056):5.366467):19.385037);

**File S5.** Phylogeny of strepsirrhines from Kistler et al. 2015.

((((((((((Propithecus_diadema:0.9185156236,Propithecus_edwardsi:0.9185156236):5.213359273,Propithecus_coquereli:6.131874896):5.593148605,Avahi_laniger:11.7250235):3.681154674,Indri_indri:15.40617818):3.776036872,(Palaeopropithecus_ingens:0.5530955529,Palaeopropithecus_maximus:0.5530955529):18.62911949):2.588280792,Hadropithecus_stenognathus:21.77049584):4.308794474,((Lepilemur_hubbardorum:10.48784422,Lepilemur_mustelinus:10.48784422):11.75423629,Mirza_coquereli:22.24208052):3.837209797):1.856331377,((((Eulemur_rubriventer:4.549721707,Eulemur_macaco:4.549721707):7.797407276,Lemur_catta:12.34712898):4.224694744,(Pachylemur_jullyi:10.34468044,Varecia_variegata:10.34468044):6.227143283):8.885617951,Megaladapis_edwardsi:25.45744168):2.478180012):19.15483506,Daubentonia_madagascariensis:47.09045675):7.471787213,(((Otolemur_crassicaudatus:16.86823991,Galago_senegalensis:16.86823991):15.86081469,Perodicticus_potto:32.7290546):5.045142118,(Loris_tardigradus:27.22302206,Nycticebus_coucang:27.22302206):10.55117465):16.78804725);

**References**

[1] Mittermeier, R.A., Louis, E.E., Richardson, M., Schwitzer, C., Langrand, O., Rylands, A.B., Hawkins, F., Rajaobelina, S., Ratsimbazafy, J., Rasoloarison, R., et al. 2010 *Lemurs of Madagascar, 3rd Ed.* Washinton D.C., Conservation International; 762 p.

[2] Schwitzer, C., Mittermeier, R., Johnson, S.E., Donati, G., Irwin, M.T., Peacock, H., Ratsimbazafy, J., Razafindramanana, J., Louis Jr, E.E., Chikhi, L., et al. 2014 Averting lemur extinctions amid Madagascar's political crisis. *Science* **343**, 842-843.

[3] Herrera, J.P. & Dávalos, L. 2016 Phylogeny and divergence times of lemurs inferred with recent and ancient fossils in the tree. *Syst. Biol.* (doi:10.1093/sysbio/syw035).

[4] Rabosky, D.L. 2014 Automatic detection of key innovations, rate shifts, and diversity-dependence on phylogenetic trees. *PloS One* **9**, e89543.

[5] Höhna, S., May, M.R. & Moore, B.R. 2015 TESS: an R package for efficiently simulating phylogenetic trees and performing Bayesian inference of lineage diversification rates. *Bioinformatics*, btv651.

[6] Morlon, H., Condamine, F. & Manceau, M. 2013 RPANDA-package. (CRAN.

[7] Alfaro, M.E., Santini, F., Brock, C., Alamillo, H., Dornburg, A., Rabosky, D.L., Carnevale, G. & Harmon, L.J. 2009 Nine exceptional radiations plus high turnover explain species diversity in jawed vertebrates. *Proceedings of the National Academy of Sciences* **106**, 13410-13414.

[8] Kistler, L., Ratan, A., Godfrey, L.R., Crowley, B.E., Hughes, C.E., Lei, R., Cui, Y., Wood, M.L., Muldoon, K.M. & Andriamialison, H. 2015 Comparative and population mitogenomic analyses of Madagascar's extinct, giant ‘subfossil’lemurs. *J. Hum. Evol.* **79**, 45-54.

[9] Mittermeier, R.A. & Nash, S.D. 2006 *Lemurs of Madagascar*, Conservation International.

[10] Freckleton, R. 2009 The seven deadly sins of comparative analysis. *J. Evol. Biol.* **22**, 1367-1375.

[11] Price, S.A. & Hopkins, S.S. 2015 The macroevolutionary relationship between diet and body mass across mammals. *Biol. J. Linn. Soc.* **115**, 173-184.

[12] Godfrey, L.R., Winchester, J.M., King, S.J., Boyer, D.M. & Jernvall, J. 2012 Dental topography indicates ecological contraction of lemur communities. *Am. J. Phys. Anthropol.* **148**, 215-227.

[13] LaFleur, M., Sauther, M., Cuozzo, F., Yamashita, N., Youssouf, I.A.J. & Bender, R. 2014 Cathemerality in wild ring-tailed lemurs (*Lemur catta*) in the spiny forest of Tsimanampetsotsa National Park: camera trap data and preliminary behavioral observations. *Primates* **55**, 207-217.

[14] Parga, J.A. 2011 Nocturnal ranging by a diurnal primate: are ring-tailed lemurs (*Lemur catta*) cathemeral? *Primates* **52**, 201-205.

[15] Maddison, W.P. & FitzJohn, R.G. 2015 The unsolved challenge to phylogenetic correlation tests for categorical characters. *Syst. Biol.* **64**, 127-136.

[16] Curtis, D.J. 2006 Cathemerality in lemurs. In *Lemurs: Ecology and Adaptation* (eds. L. Gould & M.L. Sauther), pp. 133-157. New York, Springer.

[17] Santini, L., Rojas, D. & Donati, G. 2015 Evolving through day and night: origin and diversification of activity pattern in modern primates. *Behav. Ecol.*, arv012. (doi:10.1093/beheco/arv012).

[18] Morlon, H. 2014 Phylogenetic approaches for studying diversification. *Ecol. Lett.* **17**, 508-525.

[19] May, M.R., Höhna, S. & Moore, B.R. 2016 A Bayesian approach for detecting the impact of mass‐extinction events on molecular phylogenies when rates of lineage diversification may vary. *Methods in Ecology and Evolution*.

[20] May, M.R. & Moore, B.R. 2016 How Well Can We Detect Lineage-Specific Diversification-Rate Shifts? A Simulation Study of Sequential AIC Methods. *Syst. Biol.*, syw026.

[21] Paradis, E., Claude, J. & Strimmer, K. 2004 APE: analysis of phylogenetics and evolution in R language. *Bioinformatics* **20**, 289-290.

[22] Condamine, F.L., Rolland, J. & Morlon, H. 2013 Macroevolutionary perspectives to environmental change. *Ecol. Lett.* **16**, 72-85.

[23] Morlon, H., Potts, M.D. & Plotkin, J.B. 2010 Inferring the dynamics of diversification: a coalescent approach. *PLoS Biol.* **8**, e1000493.

[24] Pyron, R. & Burbrink, F. 2012 Trait‐dependent diversification and the impact of palaeontological data on evolutionary hypothesis testing in New World ratsnakes (tribe Lampropeltini). *J. Evol. Biol.* **25**, 497-508.

[25] Rabosky, D.L., Grundler, M., Anderson, C., Shi, J.J., Brown, J.W., Huang, H. & Larson, J.G. 2014 BAMMtools: an R package for the analysis of evolutionary dynamics on phylogenetic trees. *Methods in Ecology and Evolution* **5**, 701-707.

[26] R_Core_Team. 2014 R: A Language and Environment for Statistical Computing. In *R Foundation for Statistical Computing, Vienna, Austria* (<http://www.R-project.org/>.

[27] Kass, R.E. & Raftery, A.E. 1995 Bayes factors. *Journal of the American Statistical Association* **90**, 773-795.

[28] Plummer, M., Best, N., Cowles, K. & Vines, K. 2006 CODA: Convergence diagnosis and output analysis for MCMC. *R news* **6**, 7-11.

[29] Pennell, M.W., Eastman, J.M., Slater, G.J., Brown, J.W., Uyeda, J.C., FitzJohn, R.G., Alfaro, M.E. & Harmon, L.J. 2014 geiger v2. 0: an expanded suite of methods for fitting macroevolutionary models to phylogenetic trees. *Bioinformatics*, btu181.

[30] Paradis, E. 2011 *Analysis of Phylogenetics and Evolution with R*, Springer Science & Business Media.

[31] Slater, G.J., Harmon, L.J. & Alfaro, M.E. 2012 Integrating fossils with molecular phylogenies improves inference of trait evolution. *Evolution* **66**, 3931-3944.

[32] Zachos, J.C., Dickens, G.R. & Zeebe, R.E. 2008 An early Cenozoic perspective on greenhouse warming and carbon-cycle dynamics. *Nature* **451**, 279-283.

[33] Nyakatura, K. & Bininda-Emonds, O.R. 2012 Updating the evolutionary history of Carnivora (Mammalia): a new species-level supertree complete with divergence time estimates. *BMC Biol.* **10**, 12.

[34] Gelman, A., Meng, X.-L. & Stern, H. 1996 Posterior predictive assessment of model fitness via realized discrepancies. *Statistica sinica* **6**, 733-760.

[35] Harvey, P.H., Pagel, M.D. & Rees, J.A. 1991 Mammalian metabolism and life histories. *Am. Nat.*, 556-566.

[36] Harmon, L.J., Weir, J.T., Brock, C.D., Glor, R.E. & Challenger, W. 2008 GEIGER: investigating evolutionary radiations. *Bioinformatics* **24**, 129-131.

[37] Blum, M.G. & François, O. 2006 Which random processes describe the tree of life? A large-scale study of phylogenetic tree imbalance. *Syst. Biol.* **55**, 685-691.

[38] Bortolussi, N., Durand, E., Blum, M. & François, O. 2006 apTreeshape: statistical analysis of phylogenetic tree shape. *Bioinformatics* **22**, 363-364.

[39] Komsta, L. & Novomestky, F. 2015 Moments, cumulants, skewness, kurtosis and related tests. *CRAN*.

[40] Pinheiro, J., Bates, D., DebRoy, S. & Sarkar, D. 2011 nlme: linear and nonlinear mixed effects models. R package version 3.1-97. In *R Foundation for Statistical Computing, Vienna* (CRAN.

[41] Pennell, M., FitzJohn, R.G., Cornwell, W.K. & Harmon, L.J. 2014 Model adequacy and the macroevolution of angiosperm functional traits. *bioRxiv*, 004002.

[42] Beaulieu, J.M., Jhwueng, D.C., Boettiger, C. & O’Meara, B.C. 2012 Modeling stabilizing selection: expanding the Ornstein–Uhlenbeck model of adaptive evolution. *Evolution* **66**, 2369-2383.

[43] Revell, L.J. 2012 phytools: an R package for phylogenetic comparative biology (and other things). *Methods in Ecology and Evolution* **3**, 217-223.

[44] Burnham, K.P. & Anderson, D.R. 2002 *Model selection and multimodel inference: a practical information-theoretic approach*. NY, Springer.

[45] Huelsenbeck, J.P., Nielsen, R. & Bollback, J.P. 2003 Stochastic mapping of morphological characters. *Syst. Biol.* **52**, 131-158.

[46] Maechler, M., Rousseeuw, P., Struyf, A., Hubert, M., Hornik, K., Studer, M. & Roudier, P. 2015 Cluster analysis extended Rousseeuw et al. (CRAN.

[47] Oksanen, J., Blanchet, F.G., Kindt, R., Legendre, P., Minchin, P.R., O’Hara, R., Simpson, G.L., Solymos, P., Stevens, M. & Wagner, H. 2013 Package ‘vegan’. *R Package ver* **254**, 20-28.

[48] Felsenstein, J. 1985 Phylogenies and the comparative method. *Am. Nat.* **125**, 1-15.

[49] Beaulieu, J.M., O'Meara, B. & Beaulieu, M.J. 2014 OUwie: Analysis of Evolutionary Rates in an OU Framework. R package version 1.46. (<http://CRAN.R-project.org/package=OUwie>.
